# Supplementary material for: Engaging high-risk groups in early lung cancer diagnosis: a qualitative study of symptom presentation and intervention preferences among the UK’s most deprived communities
Source: BMJ Open. 2019 May 22;9(5):e025902. doi: 10.1136/bmjopen-2018-025902 (PMC6538016; doi:10.1136/bmjopen-2018-025902)
Supplement: Supplementary file 1 [file bmjopen-2018-025902supp001.pdf]

Supplementary File 1. Consolidated criteria for reporting qualitative studies (COREQ): 32-item checklist with page numbers to indicate section of the article [34]

Checklist item	Questions to consider	Page number in article
**Domain 1: Research team and reflexivity**		
Personal Characteristics		
1. Interviewer/facilitator	Which author/s conducted the interview or focus group?	4
2. Credentials	What were the researcher's credentials? *E.g. PhD, MD*	4
3. Occupation	What was their occupation at the time of the study?	4
4. Gender	Was the researcher male or female?	4
5. Experience and training	What experience or training did the researcher have?	4
Relationship with participants		
6. Relationship established	Was a relationship established prior to study commencement?	3
7. Participant knowledge of the interviewer	What did the participants know about the researcher? e.*g. personal goals, reasons for doing the research*	3
8. Interviewer characteristics	What characteristics were reported about the interviewer/facilitator? e.g. *Bias, assumptions, reasons and interests in the research topic*	4
**Domain 2: study design**		
Theoretical framework		
9. Methodological orientation and Theory	What methodological orientation was stated to underpin the study? *e.g. grounded theory, discourse analysis, ethnography, phenomenology, content analysis*	4
Participant selection		
10. Sampling	How were participants selected? *e.g. purposive, convenience, consecutive, snowball*	2-3
11. Method of approach	How were participants approached? e.*g. face-to-face, telephone, mail, email*	2-3
12. Sample size	How many participants were in the study?	2-5, 8
13. Non-participation	How many people refused to participate or dropped out? Reasons?	4
Setting		
14. Setting of data collection	Where was the data collected? e.*g. home, clinic, workplace*	4
15. Presence of non-participants	Was anyone else present besides the participants and researchers?	4
16. Description of sample	What are the important characteristics of the sample? *e.g. demographic data, date*	4,5,8
Data collection		
17. Interview guide	Were questions, prompts, guides provided by the authors? Was it pilot tested?	Appendix 2-4

18. Repeat interviews	Were repeat interviews carried out? If yes, how many?	N/A
19. Audio/visual recording	Did the research use audio or visual recording to collect the data?	4
20. Field notes	Were field notes made during and/or after the interview or focus group?	4
21. Duration	What was the duration of the interviews or focus group?	4
22. Data saturation	Was data saturation discussed?	3
23. Transcripts returned	Were transcripts returned to participants for comment and/or correction?	4
**Domain 3: analysis and findings**		
Data analysis		
24. Number of data coders	How many data coders coded the data?	4
25. Description of the coding tree	Did authors provide a description of the coding tree?	4
26. Derivation of themes	Were themes identified in advance or derived from the data?	4
27. Software	What software, if applicable, was used to manage the data?	4
28. Participant checking	Did participants provide feedback on the findings?	4
Reporting		
29. Quotations presented	Were participant quotations presented to illustrate the themes / findings? Was each quotation identified? e.*g. participant number*	6,7,9
30. Data and findings consistent	Was there consistency between the data presented and the findings?	4-8
31. Clarity of major themes	Were major themes clearly presented in the findings?	4-8
32. Clarity of minor themes	Is there a description of diverse cases or discussion of minor themes?	4-8
