# Supplementary material for: Engaging high-risk groups in early lung cancer diagnosis: a qualitative study of symptom presentation and intervention preferences among the UK’s most deprived communities
Source: BMJ Open. 2019 May 22;9(5):e025902. doi: 10.1136/bmjopen-2018-025902 (PMC6538016; doi:10.1136/bmjopen-2018-025902)
Supplement: Supplementary file 2 [file bmjopen-2018-025902supp002.pdf]

Supplementary File 2: Interview topic guide

Interview prologue

- Introduce the researcher, ensure the participant is comfortable.
- Explain purpose of the interview:
  - *“We are interested in lung health.*
  - *We would like to know how people cope with lung symptoms and how they decide if they should go to the doctor or not.*
  - *Some of the questions I ask during the interview will be about smoking. I’m not here to tell you to stop smoking; I’m just interested if you have any experiences of smoking.*
  - *I’m not a clinician so I can’t give you any advice on symptoms, but I can tell you where you can go to get help”*

- Check understanding of interview purpose, role of researcher, and what will happen in the interview. Give opportunity for questions.
- Partners or family members will be welcome to join the interviews to explore relational influences on lung symptom awareness and help seeking, and the interview topic guide will be adapted accordingly.
- After establishing what is understood about the study, and answering any questions, explain that the interview will be recorded. Obtain consent for the interview and for the recording. If not already done, set up and switch on the recording equipment while the participant signs the consent form.

Note to interviewer: the grey boxes signify the key topics to be explored during the interview. The questions listed below are examples of suggested prompts for each topic. You are not required to read these verbatim unless specified.

Overall aim of the qualitative interview:
-To explore how people with a history of smoking and respiratory conditions interpret and act on new or changing lung symptoms (how people cope with lung symptoms and how they decide to go to the doctor with symptoms)
-To explore the influences of perceived risk, fear, shame, stigma, family and friends on lung symptom presentation

1. Experiences of lung condition

Establish what lung condition the participant has, how the lung condition affects them, the types of symptoms they experience and how they are usually managed.

Rationale: introduce the participant to the format of a qualitative interview and make the participant feels comfortable. Establish details of their lung condition.

Potential prompts:

- Can you tell me about your lung condition?
- Tell me about your experiences with X lung condition.
    - How long have you had X condition?
    - What sort of symptoms do you experience?
    - How long do the symptoms usually last?
- What do you think causes these symptoms?
- How do you usually manage your symptoms?
- How are your symptoms usually managed/ treated by your healthcare professional?
    - How effective do you think this is?

2. Symptom attribution task

Use as a tool for prompting an in-depth understanding of symptom attributions and confidence to interpret new or changing symptoms.
Include discussion around previous symptom experiences including: what action was taken, if and who they sought medical help from. Explore how their lung condition and smoking history might influence symptom attributions (i.e. do these mask symptoms?) and symptom presentation.

Rationale: to explore lung symptom attribution; confidence in recognising and articulating symptoms; previous symptom experience; planning when/how to act on symptoms; influence of smoking history on perceptions of lung symptoms

Ask participant to order symptom cards from symptoms that they would seek medical help quickest for and those they would seek help slowest for (arrow and boxes below will be stretched to A4 size, see page 10). Ask participant to write numbers on the symptom cards. 1= first, 11=last. If they change the order of the symptom, ensure the previous number is crossed out and the new number written on card. Write PID on the back of each symptom card. Take photos of the task.

Say the following phrase verbatim: “We know that people decide go to the doctor at different times with symptoms. I’m going to show you some pieces of card with symptoms on. I’d like you to rank them from the ones that you would go to the doctor with first through to the last on this sheet of paper.”

Cut individual symptom cards (see page 12) for the following symptoms. Please rotate the order:

- Coughing up blood
- A cough that doesn’t go away
- A long standing cough that gets worse
- Pain in your chest or shoulder
- Persistent breathlessness
- Persistent chest infections
- Persistent tiredness or lack of energy
- Loss of appetite or unexplained weight loss
- Ache or pain when breathing or coughing
- A hoarse voice
- Changes in the appearance of your fingers, such as becoming more curved or their ends becoming larger

See the **glossary of terms at the end of the topic guide** with standardised definitions and additional explanation of symptoms. If the participant does not understand what each symptom means, ask them what they think it means, then refer to the glossary of terms.

First to go to the doctor

↑

Last to go to the doctor

If the participant asks the interviewer what these symptoms are, first **ask the participant what they think they are**. If they ask again either suggest to move on and discuss what they could be after the interview (if appropriate) or say the symptoms have been taken from the NHS website for lung cancer.

Note to interviewer: Aim to the complete the ordering of symptoms in the task first, and then ask questions in this section. However, if any of the following (2a, 2b, 2c, 2d) if bought up spontaneously by the participant whilst they are doing the card sorting exercise then explore these issues at that point. Then return to the card sorting exercise, followed by questions in the following section (2a, 2b, 2c, 2d).

Topics and prompts for symptom attribution task
Once the symptoms have been ordered, ask:

- Can you tell me why you put x first?
- Can you tell me why you put x last?

<u>2a. Symptom experience</u>

To understand if the participant has experienced the symptom previously- what did they attribute the symptom to? What did they do? If they have not experienced the symptom before, what would they hypothetically do if they were to experience symptom in the future?

- Which of these symptoms have you had before?
  - What did you think the symptom(s) was/were?
  - Which symptom(s) did you go to the doctor with?
    - Why did you decide to go to the doctor with these symptoms?
    - What did the doctor say?
  - Which symptoms did you decide to not go to the doctor with?
    - Why did you decide not to go to the doctor with these symptoms?
- For the other symptoms we have not talked about, if you developed any of them what would you do?
  - How long do you think it would take you to go to the doctor with these symptoms?
  - Can you tell me why it would take you this amount of time to go to your doctor with this symptom?
  - What you think these symptoms could be?
- Are there any symptoms you wouldn't go to the doctor with?
  - Can you tell me why you wouldn't go to the doctor with these symptoms?

2b. Detecting change

Explore how the participant has/would notice new or changing symptoms and their confidence to detect new and changing symptoms. Ask this section generally, but if the participant is struggling then ask them about confidence to detect new and changing symptoms in the context of previous symptoms experiences. i.e. earlier you mentioned you felt breathless for a long time, how confident did you feel to know that this was a new or changing symptom?

- How would you normally tell if any lung symptoms have changed/ are new/ or unusual for you?
    - What would you do if you think you noticed a change in a symptom?
    - How confident would you feel in recognising a change in your usual symptom(s)?
    - How confident would you feel recognising new symptoms?
    - How would you notice a change in any of these symptoms?

2c. Barriers

Explore the influences of barriers and social influences on lung symptom presentation behavior

- Can you think of anything that would influence your decision to go to the doctor with any of these symptoms?
    - Probe barriers/enablers to going to the GP with a symptom:
        - Transport
        - Long wait times/ appointment policies
        - Worry about wasting the doctors time i.e. Some people have told us that they don’t go to the doctor as they worry about wasting the doctors time. What do you think about this?
        - The influence of partner/ social influences (who suggested you go/ don’t go to the doctor?)
        - How confident would you feel talking to the doctor about these symptoms?
            - How do you feel when you are talking to the doctor?

2d. Smoking

Explore the influence of smoking habit on the ability to notice new or changing symptoms. If/how smoking habit acts as a barrier to seeking medical help. If vaping comes up say something like- that is really interesting, do you mind if I ask you some questions later about vaping if we have time? If they vape then still explore the influence of past smoking on symptom perceptions.

- Would you expect to have any of the symptoms we have talked about because you smoke or used to smoke?
    - Thinking about smoking, how do you think your [past] smoking would affect how you notice if a symptom has changed or is new/ unusual for you?
    - Does your doctor ever discuss smoking with you?
        - How does this make you feel?

▪

3. Lung cancer

If appropriate, explore lung cancer knowledge, beliefs about lung cancer and perceived risk here. If bought up spontaneously by the participant earlier in the interview, explore lung cancer then (if appropriate) Confidence to detect a lung cancer symptom. The influences of smoking and perceived risk, shame and associated with lung cancer.

Rationale: to explore lung cancer symptom awareness; to explore perceived risk of lung ca emotional consequences such as fear, fatalism blame; perceived causes and effectiveness of e detection and treatment; the influence of smol history on the formation of beliefs about lung c

If it is OK with you, I’m going to ask you some questions about lung cancer. If you feel uncomfortable with any of my questions, please let me know and we can move on.

Potential prompts:

- Earlier you mentioned some of the symptoms that could be lung cancer. Can you think of any other symptoms that you think might be lung cancer?
- What else do you know about lung cancer?
- Can you tell me a little more about what you think about lung cancer as a disease?
    - Where do you think that feeling comes from?
    - What do you think other people’s views are on lung cancer?
- What do you think causes lung cancer? S
    - If yes and appropriate: How does that make you feel as a [past] smoker?
    - If appropriate: do you ever worry about lung cancer because you [used to] smoke?
    - How does that make you feel?
    - Is there anything in particular that makes you think your risk is high or low?
- How confident would you feel in recognising a symptom that could be lung cancer?
    - How does your [past] smoking affect your confidence in recognising a change in your body that could be lung cancer?
- What would you do if you had a symptom that you thought was lung cancer?

4. Social influences

Explore influences of social networks on help seeking behavior and basis of cancer beliefs.

Rationale: to explore social norms, influences and stigma around help seeking; people who they know who have had lung cancer and the effects on beliefs and perceptions; how social networks might influence help-seeking

- If you had a symptom you thought might be lung cancer, would you tell anyone about it?
    - Who would you tell?
    - Why would you tell that person?
    - What do you think they would say to you if you told them about a symptom?
    - Would they encourage/discourage you to visit your doctor?
- Has anyone ever noticed a symptom of yours and suggest that you go to the doctor?
    - Probe: who/ what happened.
- We know that lung cancer isn't as common as other types of cancer like breast cancer. Do you know anyone who has ever been diagnosed with lung cancer?
- Can you tell me about the effects that [person] having lung cancer has had on you?
    - How has this affected your views about lung cancer?

5. Closing questions

Final questions to end on a positive note

Rationale: to end the interview on a positive note

- If you were to offer anyone some advice on lung symptoms, what would it be?
- If the NHS were to make some changes to the services they offer to help people with lung symptoms get seen quicker, what would you suggest they change?
- Can you tell me about a positive experience of going to the doctor?

I know we've talked about a lot of things today, but would you like to tell me about anything else that we haven't talked about?

Debrief

- Summarise the interview and address any questions or concerns.
- Check whether it is ok to contact them if there is anything that needs to be clarified after listening back to the conversation.
- Ensure that they know how to contact us for further help/information/to add further information
- Thank them for their time and give them the gift voucher.
- If the participant discloses symptoms during the interview suggest they seek medical help from their GP. Offer lung cancer leaflet and site specific helpline numbers.
- Have stop smoking service details available if they request it
- Offer to provide a summary of study findings

Helpline numbers

Wales

- Tenovus Cancer Care support line on 0808 808 1010. The support line is open 8am-8pm, 7 days a week. Calls are free from a BT landline.

England and Scotland

- British Lung Foundation helpline on 03000 030555. This helpline is open 9am-5pm Monday to Friday. Calls cost the same as a local call.
- Roy Castle Lung Cancer Foundation helpline on 0333 323 7200. This helpline is open 9am-5pm Monday to Friday. Calls are free from a BT landline.

Symptoms in the symptom task:

Coughing up blood

A cough that doesn’t go away

A long standing cough that gets worse

Pain in your chest or shoulder

Persistent breathlessness

Persistent chest infections

Persistent tiredness or lack of energy

Loss of appetite or unexplained weight loss

Ache or pain when breathing or coughing

A hoarse voice

Changes in the appearance of your fingers, such as becoming more curved or their ends becoming larger

__Glossary of terms__

Coughing up blood

If you notice that there is some blood coming up when you cough

A cough that doesn't go away

If you have a nagging cough that just doesn't seem to go away

A long standing cough that gets worse

You have had a cough for a long time but you think that it might have got worse

Pain in your chest or shoulder

A sharp or achy pain in either your chest or shoulder, or both

Persistent breathlessness

You feel like you can't catch your breath or become out of breath when doing tasks you used to be fine with

Persistent chest infections

If you have had a few chest infections in a row and they don't seem to be getting better or keep coming back

Persistent tiredness or lack of energy

If you have been feeling tired for a while or just feel like you don't have any energy

Loss of appetite or unexplained weight loss

If you have been loosing weight without trying to and can't explain why or you just haven't been feeling up to eating the amount you normally would for a while

Ache or pain when breathing or coughing

If you have a sharp pain or achy feeling when you cough or breathe

A hoarse voice

A croaky or gravelly voice

Changes in the appearance of your fingers, such as becoming more curved or their ends becoming larger

If you notice that the ends of your fingers have changed shape

Persistent definition

Something that you notice you have had for a while and won't go away.

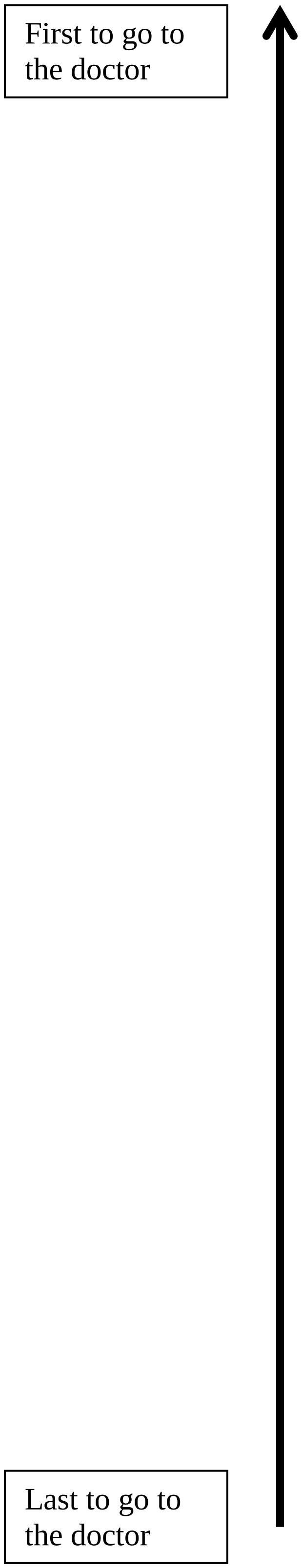
First to go to the doctor
Last to go to the doctor
