# Supplementary material for: Engaging high-risk groups in early lung cancer diagnosis: a qualitative study of symptom presentation and intervention preferences among the UK’s most deprived communities
Source: BMJ Open. 2019 May 22;9(5):e025902. doi: 10.1136/bmjopen-2018-025902 (PMC6538016; doi:10.1136/bmjopen-2018-025902)
Supplement: Supplementary file 3 [file bmjopen-2018-025902supp003.pdf]

Supplementary File 3: HPCP focus group topic guide

Focus group aim: To explore the needs and preferences from members of the public and local stakeholders (healthcare professionals/ community partners) for an intervention to support earlier lung cancer detection and diagnosis, targeted at high risk, harder to reach groups (over 40's, who are current/former smokers, living in areas of deprivation with serious lung comorbidity i.e. COPD).

Introduction

- Explain the aims of the focus group discussion. Emphasise that the focus group is not a test; we are interested in participants' preferences for an intervention for "lung health" to prompt earlier lung cancer symptom presentation, and how we could access people to take part in an intervention. All comments are welcomed: positive and negative.
    - 'We know that people who are high risk for lung cancer (people over the age of 40, living in deprived areas, who currently smoke or used to smoke and have a lung condition like COPD) are often diagnosed at a late stage, where treatment options are limited and a cure is less likely. This might be because they delay seeking medical advice with symptoms suggestive of lung cancer.

      We are considering if we should develop something that will give people who are high risk for lung cancer information about symptoms and when/ where they should go and seek medical help from.

      As healthcare professionals and people who work in deprived communities, we would like to know what you think about if we should do this. If you think this is a good idea, we would also like to know how best we can access this target group and invite them to take part in an intervention about lung health. Your ideas and suggestions are really valuable to us because of your wealth of experience, so all comments are welcomed (both positive and negative)'.
- Explain the voluntary nature of the study and that the focus group will be recorded with permission. If not already done, set up and switch on the recording equipment while participants sign the consent form.
- Before starting the focus group, remind participants about confidentiality and ask participants not to talk over each other. Go around the circle and ask participants to introduce themselves for the transcription.

> ***Rationale:*** to explore whether the findings resonate with patients and members of the public in their local communitv

Part1. Phase 1 interview findings

Discussion of Phase 1 interview findings

Provide a short overview of key findings from Phase 1 interviews regarding barriers and enablers to lung cancer awareness and early symptom presentation in the target group. Ask the group to share their thoughts on the interview findings, and whether they resonate with them.

'We have been interviewing people across the UK who are high risk for lung cancer- people over the age of 40 who have a lung condition like COPD, who currently smoke or used to smoke and live in deprived areas. We wanted to understand how people think about symptoms of lung cancer, how and when people decide to go to the doctor with lung symptoms and the barriers to going to the doctor with these symptoms.

We found that people are completely fixated on detecting chest infections and look out for symptoms of a chest infection most days. They were really good at knowing when they had a chest infection and going to the doctor quickly to get antibiotics because they know it can be treated.

We also found that people tend to deny or ignore health problems that might affect them in the future, like lung cancer so may not go to the doctor with some serious symptoms. We think this is because they think that although lung cancer is inevitable, they also think that it cannot be cured so don't go and see the doctor

We want to develop something that can help people get these important symptoms of lung cancer seen to quicker by a medical professional, but we are not really sure how to do this. We would like to know what you think.'

- How can we do this?
- What do you think of these findings?
- To what extent do these findings resonate with you?

> ***Rationale:*** to seek views on how to access the target group for an intervention and explore preferences for an intervention to support earlier presentation, including mode of delivery, target group, content and stop smoking information

Preferences on intervention content

- What sorts of things do you think people would like to know / what skills would they like to learn?
    - What do you think would be most useful to people?
    - What information would be the highest priority/most important for them?
    - Should we avoid any information?
    - Do you think it is best to focus on symptoms or health beliefs?

Preferences on intervention format

- How can we support people to seek medical help quickly with lung cancer symptoms? [what format]
    - Probe: a group one-off educational session to promote "lung health" in the local community; a leaflet/DVD; a lung health check; event in the community; posters in the local community
    - Do you think people want education or to learn 'skills for health'?
        - What sort of skills do you think people would like to know?
- Who do you think would be best to lead or facilitate an intervention about lung health/ encourage people to seek medical help quickly with lung cancer symptoms?
    - Probe: patient representative, lay advisor, community partner, healthcare professional (GP, practice nurse, smoking cessation counsellor, respiratory nurse specialist…?)
- When would be best to support people to manage their lung health?
    - What do you think about using the point at which they are diagnosed with a lung condition as a 'teachable moment' to do an intervention about lung cancer?
    - What about during a regular check-up appointment with the nurse i.e. six monthly COPD clinic reviews?
- What do you think about a brief intervention with health care professionals in addition to a public facing intervention?
    - What would be useful to you as healthcare professionals?
- The intention is that this intervention would be implemented across the UK. How do you think everything we have talked about today might need to differ based on where someone lives (i.e. different countries) or the person in the intervention?
    - How could we incorporate an intervention into the different models of care across regions/countries?
- How do you think this intervention could fit with other current or planned health promotion activities?

Accessing intervention participants

- Where could we approach our target group in an intervention about lung cancer/lung health?
- Probe: through community pharmacies, primary care, community nurses, existing groups for people with lung conditions?
    - Probe: on utilising family / social networks to encourage participation of MoP?
- Are you aware of any existing groups for people who fall into our target group?
    - What do you think about "piggybacking" onto these existing community groups or existing care plans with the nurse?
    - How do you think people would react to this?

Smoking cessation

- 'We know that people really don't like it when the doctor tells them to stop smoking and some people felt like they were treated differently by the doctors because they smoked. Sometimes this put people off going to the doctor with lung symptoms. On the other hand some people feel that it is only fair to be told to stop smoking. When we design something about lung cancer, we don't know if we should include something to help people to stop smoking or not.'
- What do you think about including stop smoking information in the intervention?
    - How do you think smokers might react to including stop-smoking information?
    - Can you think of ways we might be able to include stop-smoking information without putting smokers off?
    - What do you think about using signposting to stop smoking services instead of providing information about smoking cessation during the session?
- What do you think about using the intervention to cover things that are not related to lung health i.e. mental health or other factors that could be related to lung health?
        - How should we approach this?

Debrief

'Thank you for taking part in this study. We hope to use the findings from this focus group to develop an intervention based on the findings from the interviews and your recommendations to encourage earlier lung symptom presentation in those who are high risk for lung cancer. Anything you said will be treated as confidential. The voice-recoding will be stored securely. Any quotes used in published research will not have your name or anything that could identify you. Do you have any questions? [answer any questions] Here are my contact details if you have any further questions.'
