# Supplementary material for: Engaging high-risk groups in early lung cancer diagnosis: a qualitative study of symptom presentation and intervention preferences among the UK’s most deprived communities
Source: BMJ Open. 2019 May 22;9(5):e025902. doi: 10.1136/bmjopen-2018-025902 (PMC6538016; doi:10.1136/bmjopen-2018-025902)
Supplement: Supplementary file 4 [file bmjopen-2018-025902supp004.pdf]

Supplementary File 4: Members of the public focus group topic guide

Focus group aim: To explore the needs and preferences from members of the public and local stakeholders for an intervention to support earlier lung cancer detection and diagnosis. The intervention will be targeted at high risk, harder to reach groups (over 40's, who are current/former smokers, living in areas of deprivation with serious lung comorbidity i.e. COPD).

Introduction

- Explain the aims of the focus group. Emphasise that the focus group is not a test; we are interested in participants' preferences for an intervention for "lung health" to prompt earlier lung symptom presentation, and how we could access people to take part in an intervention. All comments are welcomed: positive and negative.
    - '*We know that some people sometimes might take a bit longer to go to the doctor with important lung symptoms. I am thinking about developing something that will highlight important lung symptoms, and encourage people to go to speak to someone who is medically trained about their symptoms. I would like to know what you think about best ways to do this. I would also like to know how you think we can find people for this. Your ideas and suggestions are really valuable to us, so all comments are welcomed (both positive and negative)*'.
- Explain the voluntary nature of the study and that the focus group will be recorded with permission. If not already done, set up and switch on the recording equipment while participants sign the consent form.
- Before starting the focus group, remind participants about confidentiality and ask participants not to talk over each other. Go around the circle and ask participants to introduce themselves for the transcription.

Rationale: to explore whether the findings resonate with patients and members of the public in their local community

Discussion of Phase 1 interview findings

Provide a short overview of key findings from Phase 1 interviews regarding barriers and enablers to lung cancer awareness and early symptom presentation in the target group. Ask the group to share their thoughts on the interview findings, and whether they resonate with them.

'We did some interviews across the UK with people who smoke or used to smoke and have a lung condition like COPD. We found that people look out for symptoms of a chest infection most days. They were really good at knowing when they had a chest infection and going to the doctor to get antibiotics. We also found that people try not to think about health problems that might affect them in the future, so may not go to the doctor with some symptoms that could be serious.

People really didn't like it when the doctor told them to stop smoking and some people felt like they were treated differently by the doctors because they smoked. Sometimes this put people off going to the doctor with lung symptoms.

We also found that people often had a favourite doctor at their GP practice and would wait up to three weeks for an appointment even if they thought a symptom was important. People don't like

going to doctors that they don't know because they felt that they did listen as much as their favourite doctor. But sometimes it was necessary to go to a doctor they don't know to get an appointment the same day.

We want to develop something that can help people get important lung symptoms seen to quicker by a medical professional, but we are not really sure how to do this or who should do this. We would like to know what you think.'

- What do you think of these findings?
- In what ways do you feel the same as what we found?
- In what ways do you feel different to what we found?

> ***Rationale:*** to seek views on how to access the target group for an intervention and explore preferences for an intervention to support earlier presentation, including mode of delivery, target group, content and stop smoking information

Part 2. Needs and preferences for an intervention

Preferences on intervention format

- In what ways could we support people to manage their lung health?
    - Probe: a group one-off educational session to promote "lung health" in the local community; a leaflet/DVD; a lung health check; event in the community; posters in the local community
- Who do you think would be best to lead an intervention about lung health?
    - Probe: lung cancer survivor, lay advisor, community partner, healthcare professional
- If a health care professional was to lead the intervention, who would be best to lead a lung health intervention?
- Who is your favourite healthcare professional?
- When would be best to support people to manage their lung health?
    - What about when someone is diagnosed with a lung condition?
    - What about in one of your regular check-up appointments with the nurse i.e. six-monthly COPD clinic review?

Preferences on intervention content

- **What would you like to know? OR what skills would you like to learn?**
    - What would be most useful or important for you?
    - Can you think of anything that we should avoid or anything that is not as important?
- What do you think about including information to help people to stop smoking?
    - How would you react to stop-smoking information?
    - Can you think of ways we might be able to include stop-smoking information without putting smokers off?
    - What do you think about telling people where they can get help to stop smoking if they want to, instead of giving people information about stop smoking?

Accessing intervention participants

- Where could we approach people who smoke/used to smoke and have lung symptoms?
    - Probe: through community pharmacies, primary care, community nurses, existing groups for people with lung conditions/ utilising family and social networks / snowballing approaches?
- Are you aware of any community groups for these types of people?
    - How do you think people from these groups would react to being approached for a lung health intervention?

Debrief

'Thank you for taking part in this study. We hope to use the findings from this focus group to develop something to encourage people to go to the doctor with important lung symptoms. Anything you said will be treated as confidential. The voice-recoding will be stored securely. Any quotes used in published research will not have your name or anything that could identify you. Do you have any questions? [*answer any questions*] Here are my contact details if you have any further questions.'
