# Supplementary material for: Engaging high-risk groups in early lung cancer diagnosis: a qualitative study of symptom presentation and intervention preferences among the UK’s most deprived communities
Source: BMJ Open. 2019 May 22;9(5):e025902. doi: 10.1136/bmjopen-2018-025902 (PMC6538016; doi:10.1136/bmjopen-2018-025902)
Supplement: Author's manuscript [file bmjopen-2018-025902.draft_revisions.pdf]

BMJ Open

BMJ Open is committed to open peer review. As part of this commitment we make the peer review history of every article we publish publicly available.

When an article is published we post the peer reviewers' comments and the authors' responses online. We also post the versions of the paper that were used during peer review. These are the versions that the peer review comments apply to.

The versions of the paper that follow are the versions that were submitted during the peer review process. They are not the versions of record or the final published versions. They should not be cited or distributed as the published version of this manuscript.

BMJ Open is an open access journal and the full, final, typeset and author-corrected version of record of the manuscript is available on our site with no access controls, subscription charges or pay-per-view fees (http://bmjopen.bmj.com).

If you have any questions on BMJ Open's open peer review process please email info.bmjopen@bmj.com

BMJ Open

Engaging high-risk groups in early lung cancer detection: a qualitative study of symptom presentation and intervention preferences amongst the UK's most deprived communities

Journal:	*BMJ Open*
Manuscript ID	bmjopen-2018-025902
Article Type:	Research
Date Submitted by the Author:	09-Aug-2018
Complete List of Authors:	McCutchan, GM; Cardiff University, Division of Population Medicine<br>Hiscock, Julia; Bangor University, North Wales Centre for Primary Care Research<br>Hood, Kerenza<br>Murchie, Peter; University of Aberdeen, Division of Applied Health Science<br>Neal, RD; University of Leeds<br>Newton, Gareth; Cardiff University, Division of Population Medicine, Patient and Public Involvement<br>Thomas, Sara; Public Health Wales, Cwm Taf Public Health Team<br>Thomas, Ann Maria; Cardiff University, Division of Population Medicine, Patient and Public Involvement<br>Brain, Kate; Cardiff University, Division of Population Medicine, School of Medicine
Keywords:	PRIMARY CARE, PUBLIC HEALTH, QUALITATIVE RESEARCH, RESPIRATORY MEDICINE (see Thoracic Medicine), lung cancer, socioeconomic status

SCHOLARONE™
Manuscripts

Title page

Title: Engaging high-risk groups in early lung cancer detection: a qualitative study of symptom presentation and intervention preferences amongst the UK's most deprived communities

Authors: Dr Grace McCutchan[1$], Dr Julia Hiscock[2], Prof Kerry Hood[3], Dr Peter Murchie[4], Prof Richard Neal[5], Mr Gareth Newton[1], Mrs Sara Thomas[6], Mrs Ann-Maria Thomas[1], Dr Kate Brain[1]

Affiliations:

[1]Division of Population Medicine, School of Medicine, Cardiff University, Cardiff, UK

[2]North Wales Centre for Primary Care Research, Bangor University, Wrexham, UK

[3]Centre for Trials Research, School of Medicine, Cardiff University, Cardiff, UK

[4]Division of Applied Health Sciences, Section of Academic Primary Care, University of Aberdeen, Aberdeen, UK

[5]Leeds Institute of Health Sciences, Faculty of Medicine and Health, University of Leeds, Leeds, UK

[6]Cwm Taf Public Health Team, Public Health Wales, Kier Hardy University Health Park, Merthyr Tydfil, UK.

[$]**Corresponding author:** Grace McCutchan. Postal address: Division of Population Medicine, Cardiff University, 1st Floor, Neuadd Meirionnydd, Heath Park, Cardiff, CF14 4YS. Email: mccutchanGM@cardiff.ac.uk. Telephone: +44 (0)2920 687639.

Word count: 4738

For peer review only

Abstract

Objectives

People at high-risk for lung cancer - current/former smokers, aged 40+ years, with serious lung comorbidity (i.e. Chronic Obstructive Pulmonary Disease) and living in highly deprived areas - are more likely to prolong symptom presentation, leading to advanced stage diagnosis. This qualitative study aimed to understand the influences on early presentation with lung cancer symptoms in high-risk individuals and intervention preferences.

Methods

Semi-structured qualitative interviews with 37 high-risk individuals, identified through seven GP practices socioeconomically deprived areas of England, Scotland and Wales (most deprived 20%). A symptom attribution task was used to explore lung symptom perception and help seeking, underpinned by Leventhal's Common Sense model. Four focus groups with high-risk individuals and local stakeholders (healthcare professionals and community partners) to explore preferences for an intervention to promote early lung cancer symptom presentation. Data were synthesised using Framework analysis.

Results

Individual and area level indicators of deprivation confirm that our sample were highly deprived. Fixation on managing 'treatable' short-term conditions (chest infections), led to denial and avoidance of 'inevitable and incurable' longer-term conditions (lung cancer). Feeling judged and unworthy of medical help because of their perceived social standing or lifestyle deterred medical help seeking, particularly when difficult life circumstances and traumatic events lead to current/former tobacco and alcohol addiction. Focus group participants suggested multi-faceted interventions in community venues, with information about lung cancer symptoms and the benefits of early diagnosis, led by a trained and non-judgemental facilitator.

Conclusions

This study was novel in engaging a high risk, highly deprived sample to provide in-depth understanding of the broader contextual influences on self-management of lung cancer symptoms. Perceived lack of health service entitlement and complex lives facilitated avoidance of recognising and presenting with lung cancer symptoms. Community-based interventions are required to empower this population to seek medical help.

For peer review only - http://bmjopen.bmj.com/site/about/guidelines.xhtml

Key words: Lung cancer, deprived, qualitative, early diagnosis, medical help seeking, barriers.

Strengths and limitations of the study

- This was the first study to use in-depth qualitative methods to explore how to engage individuals who are highest risk for lung cancer in early lung cancer detection.
- A major strength of this study was the proactive and rigorous sampling procedures used, to ensure that all interview participants recruited were at highest risk for lung cancer.
- Assessment of individual and area level indicators of deprivation confirm that interview participants were highly deprived; all participants resided in the most deprived 20% areas of three nations of the UK, and most participants were unemployed/seeking benefits, and/or rented social housing.
- To overcome the methodological limitations associated with studying anticipated or retrospectively recalled cancer symptom presentation, we recruited participants with no previous diagnosis of lung cancer and did not mention lung cancer in the interview study materials or during completion of the symptom attribution task.
- Opportunistic recruitment of focus group participations was a potential limitation of the study as participants may be more favourably disposed to an intervention.

For peer review only - http://bmjopen.bmj.com/site/about/guidelines.xhtml

INTRODUCTION

Lung cancer is the leading cause of cancer mortality worldwide[1]. Outcomes are among the poorest for all cancers, with only 10% of lung cancer patients surviving five or more years in the UK[2]. Diagnosis of lung cancer at an early stage can enable curative surgical resection, meaning over 80% of patients will survive one year or more when diagnosed at Stage I[3]. Delayed medical help seeking for symptoms and the high proportion of lung cancer diagnoses through emergency departments may partly explain why lung cancer is commonly diagnosed at an advanced, incurable stage[4]. Due to low specificity of lung cancer symptoms and similarity to other acute and comorbid respiratory conditions, patients face difficulty in knowing when to seek medical help[5,6].

Multiple symptoms and risk factors for lung cancer including older age, smoking, the presence of a lung comorbidity and socioeconomic deprivation increase the likelihood that a symptomatic individual has lung cancer[7-9]. Lung cancer is more common and mortality higher in areas of high socioeconomic deprivation; it has been estimated that each year, socioeconomic inequalities account for 11,700 excess cases of lung cancer and 9,900 potentially avoidable lung cancer deaths in England[10]. High prevalence of smoking, lung comorbid conditions and industry-type work such as mining, all of which are well documented risk factors for lung cancer, contribute to high lung cancer incidence and mortality in deprived communities[11, 12].

The presence of lung comorbidity such as chronic obstructive pulmonary disease (COPD) and history of smoking have been associated with a lower likelihood of presenting with lung cancer symptoms early[13,14]. In the lead up to lung cancer diagnosis, vague symptoms may go unnoticed, or be misattributed to smoking, aging or other comorbid conditions such as heart disease or emphysema, thereby prolonging help seeking[5,13,15-26]. In addition, stigma attached to lung cancer[23,27-30] and fear of lung cancer diagnosis can deter medical help seeking for symptoms, particularly among smokers[23-25,29,31-33], leading to advanced stage disease at diagnosis[19]. To date, research has mainly been conducted with lung cancer patients from a range of socioeconomic groups, retrospectively exploring the barriers to symptom presentation.

The current study used a combination of interviews and focus groups to gain in-depth understanding of the influences on lung cancer symptom presentation behaviour and intervention preferences in high risk, highly deprived groups. We targeted socioeconomically deprived areas across three nations of the UK to approach potential participants, and used rigorous sampling procedures to ensure that our sample were at the highest risk for lung cancer. The focus groups, conducted in

For peer review only - http://bmjopen.bmj.com/site/about/guidelines.xhtml

highly deprived areas, explored intervention preferences with stakeholders who live or work in deprived communities. To our knowledge, this was the first study to explore the influences on early lung cancer detection and intervention preferences in high risk groups living in the most deprived quintiles in the UK.

METHODS

The Consolidated Criteria for Reporting Qualitative Research (COREQ)[34] criteria were used to guide reporting (Supplementary File 1).

Participant recruitment and sampling

Interviews. Interview participants were recruited through seven general medical practices (GP) in South Wales (Cwm Taf, n=3), England (Liverpool, n=1) and Scotland (Aberdeen, n=3). GP practices with the highest proportion of their patients that reside in the lowest quintile of deprivation were contacted. Practice managers were asked to screen GP practice databases purposively for eligible study participants: men and women over the age of 40, who were current or former smokers, with a lung condition (COPD including chronic bronchitis and emphysema, interstitial lung disease or occupational lung disease). Participants were sampled purposively according to smoking status due to an initially high response rate from former smokers. GP practices in Cwm Taf were asked to recruit current and former smokers, with no parameter on number of years since quit attempt. One GP practice in Aberdeen was asked to recruit current smokers and recent quitters (within ten years). Two GP practices in Aberdeen and one GP practice in Liverpool recruited current smokers only.

To ensure that participants from highly deprived areas were invited to take part in the study, individual postcodes were screened by the research team. Eligible patients from the initial database screen were assigned a pseudo-anonymised participant identifier (PID). PID and postcode were checked against Index of Multiple Deprivation score, and those that resided in the most deprived quintile were eligible for the study. The final list of potentially eligible participants was checked by the GP for ability to provide informed consent, uncontrolled drug or alcohol misuse or violence (i.e. considered by the GP to be a risk to the interviewer or themselves) and general health status (i.e. very seriously ill). Participants were excluded if they were diagnosed with lung cancer, were terminally ill or did not have capacity to consent.

For peer review only - http://bmjopen.bmj.com/site/about/guidelines.xhtml

Focus groups. Participants for the focus groups with members of the public were recruited opportunistically through primary care or local community groups. Participant recruitment through primary care employed the same methods as those used to invite the interview participants. Pseudonymised participant identifiers were checked to ensure that those who took part in the focus groups had not already participated in the interviews. Additional participants were recruited opportunistically through local community respiratory support groups and non-health related groups in the local community centre. Local community group organisers in areas of high deprivation were contacted and asked for help to recruit members of the public in our target group. Local health service planning groups and health board staff facilitated recruitment for the healthcare professional and community partner (HPCP) focus groups.

Study procedures

Written consent and permission to audio-record were obtained prior to the interviews and focus groups.

Interviews. Eligible participants were invited by letter with more detailed study information attached, with a reminder at two weeks to non-respondents. Those consenting were contacted by the interviewer (GM or JH) to arrange a suitable time and date for the interview, to outline the study and answer any questions.

Interviews were conducted using a semi-structured topic guide based on the Common Sense Model to guide discussion around illness perceptions and coping strategies[35]. The interview aimed to explore experiences of their lung condition, symptom attribution, symptom experience and help seeking behaviour, the influence of smoking history on new or changing symptoms, and lung cancer awareness and beliefs.

A symptom sorting task was used to provide participants with a concrete visual task to increase engagement with the interview in the context of potential low literacy. The task formed a basis for discussion about symptom attribution and experience, where participants were asked to order 11 symptoms from those they would go to the doctor with first, through to the last. The 11 symptoms were selected from the NICE guidance for referral of suspected lung cancer. The symptoms were re-worded to simplify the language in line with wording found on the NHS Choices website for lung cancer symptoms and any reference to time scale of symptoms was removed (Supplementary file 2).

For peer review only - http://bmjopen.bmj.com/site/about/guidelines.xhtml

For example, a cough that lasts for 3 weeks or more was amended to 'persistent cough', and haemoptysis was amended to 'coughing up blood'. The presentation order of the symptoms was rotated between interviews.

To explore potential lung cancer symptom attribution outside of a cancer context, there was no mention of cancer in the study information packs or when participants completed the symptom sorting task. If appropriate, participants were asked questions to explore lung cancer awareness and beliefs at the end of the interview or when participants discussed lung cancer unprompted.

Demographic data were collected using a short questionnaire, including three additional measures of socioeconomic group: age, gender, smoking status (quantity and duration), home ownership, occupation and educational attainment. Interviews were conducted until data saturation (no new themes emerging[36]).

Focus groups*.* Healthcare professionals (i.e. GP, nurse, community pharmacist) and community partners (i.e. community worker) who work in areas of deprivation, and with people with smoking history and/or lung conditions were sent information about the study and invited to take part in the focus group. A mutually convenient time, date and location for the focus groups was agreed. The focus groups were conducted using a semi-structured topic guide to explore preferences for an intervention to promote earlier lung cancer diagnosis. Separate topic guides were used for the public and professional groups. Topics for discussion were: preferred format of the intervention, recommendations for intervention content, preferred location and facilitator for intervention delivery, and recommendations for the inclusion of smoking cessation advice.

Setting

Most interviews (n=34) took place face-to-face in participant's own homes, with three taking place in a café, local community centre or over the telephone, and lasted between 46 and 146 minutes (mean 83 minutes). Family members were present for three interviews but did not participate in the study. Focus groups took place in primary care settings (n=2) or local community centres (n=2). Members of the public who took part in the interviews or focus groups were given a £10 shopping voucher to thank them for their time. Healthcare professionals and community partners were not reimbursed for their time.

For peer review only - http://bmjopen.bmj.com/site/about/guidelines.xhtml

Interviews and focus groups in England were conducted by JH (PhD), a trained and experienced female qualitative Research Fellow and Medical Sociologist. The Welsh and Scottish interviews and focus groups were conducted by GM (PhD), a female Health Psychologist and trained qualitative Research Associate.

Data analysis

Interviews and focus groups were audio-recorded and transcribed verbatim. Anonymised transcripts were analysed in detail using the Framework method[37]. The data were analysed in five stages: familiarisation, identification of a thematic framework, indexing, charting, and interpretation. A separate index was created on Microsoft Excel for the interview and focus group data; however, wherever possible, overlap was coded using the same indexing terms, for example 'barriers to symptom presentation' was commonly discussed in both the interview and focus groups. The index was developed by two researchers (GM and JH). Themes were generated independently and consolidated through discussion in nine interpretation workshops over a nine month period by GM and JH. The different perspectives of the researchers as noted above was a benefit during analysis and interpretation. Field notes were recorded for each interview and focus group, and incorporated into discussion during the analysis workshops. Interpretive themes were generated by JH and GM, and developed with all authors in monthly management meetings. Transcripts and study findings were not checked by participants; however, all participants were mailed a summary of the study findings.

Ethical approval

The study received ethical approval from Southampton Central- Hampshire A Research Ethics Committee (16/SC/0589).

Patient and public involvement. Patient and public representatives (AMT and GN) were involved in the design of the study and interpretation of study findings in monthly management group meetings. All study materials and topic guides were developed with lay input (AMT and GN) and written to a reading age of 10 years due to potentially low literacy.

For peer review only - http://bmjopen.bmj.com/site/about/guidelines.xhtml

RESULTS

Interviews

Seventy-eight returned the study reply slip and declined to participate in the study; reasons for refusal unknown. The majority of our sample were female, current smokers, and with a mean age of 65 years (Table 1). Most had a diagnosis of COPD. All 37 participants resided in the lowest quintile of deprivation for their respective country, of whom 15 were in the most deprived decile. Most participants had left school before age 15 with no formal qualifications, lived in social housing, and claimed disability benefit or job seekers allowance.

Table 1. Qualitative interview sample characteristics

Sample characteristics	Total n=37
**Gender**	
Male	16
Female	21
**Age, years**	
Mean (range)	64.7 (48-84)
**Smoking status**	
Current smoker	18
Occasional smoker	3
Former smoker, recent quitter (within five years)	5
Former smoker (quit over five years ago)	11
**Deprivation decile**[+]	
Welsh Index of Multiple Deprivation (WIMD)	
Decile 1 (most deprived 10%)	5
Decile 2 (most deprived 10-20%)	10
Scottish Index of Multiple Deprivation (SIMD)	
Decile 1 (most deprived 10%)	4
Decile 2 (most deprived 10-20%)	12
English Index of Multiple Deprivation (IMD)	
Decile 1 (most deprived 10%)	6
**Self-reported lung condition**	
COPD	26
Chronic bronchitis	2
Chronic emphysema	2
Occupational lung disease	1
Unsure of diagnosis	4
Missing	2
**Educational attainment**	
Left school at/before age 15	29
Completed CSEs, O-Levels or equivalent	5
Completed A levels or equivalent	1
Completed further education but not degree	1
Missing	1

For peer review only - http://bmjopen.bmj.com/site/about/guidelines.xhtml

For peer review only

**Employment**	
Employed full-time	2
Employed part-time	1
Casual work	1
Job seekers or disability benefit	17
Retired	16
**Home/living arrangement**	
Own flat/house	14
Rent from local authority/housing association	21
Rent privately	1
Missing	1

$^{+}$Calculated using participant postcode and local authority data

Key themes were: strategies involved in symptom detection and help seeking behaviour, fixation on maintaining short term health, denial and avoidance of longer term health, the desire to be a model patient, and the importance of the relationship with their healthcare professional. See Figure 1 for illustrative quotes.

Symptom detection strategies and help seeking

Lung cancer symptoms were viewed as “part and parcel” (male, 68, England, current smoker) of their lung condition, other pre-existing comorbidities or smoking habit, and were consequently normalised and perceived not to require medical help. Changes to vague or respiratory-type lung cancer symptoms were only taken seriously when remarked on by friends and family or when they impacted on daily life.

Symptoms that could indicate a chest infection were constantly monitored. Participants used sophisticated strategies such as noticing changes in the colour and consistency of their phlegm or subtle audible changes in their cough to actively detect chest infections. Such strategies were used to facilitate early detection and treatment for chest infections through their primary care provider or with rescue packs (emergency packs of steroids and antibiotics that can be kept at home), due to lung condition.

Constant monitoring of phlegm for control of lung condition meant that participants could and would notice haemoptysis, but few reported actively looking for haemoptysis. Disparity between actual and anticipated medical help seeking was found for haemoptysis. Most participants had not previously experienced haemoptysis, but would anticipate seeking medical help immediately due to

For peer review only - http://bmjopen.bmj.com/site/about/guidelines.xhtml

the potentially serious nature of blood. However, some participants who had previously or were currently experiencing haemoptysis reported normalisation, leading to delays in medical help seeking or no help seeking.

Focus on maintaining short term health

When symptoms were easy to detect, they were attributed to what was perceived as a treatable cause and represented an immediate health threat i.e. a chest infection due to lung condition, participants sought medical help quickly. Participants could often request an appointment the same day as permitted by their GP surgery policies. Prompt help seeking was due to fear of not being able to breathe and the potentially life-threatening nature of chest infections, and is likely to reflect the need to maintain good health in the short term.

The focus on maintaining short term health may reflect low general expectations of health, where some participants were surprised to live past 60 years of age. In addition, due to fear of potentially hearing bad news, some participants expressed a preference to not ask questions during a consultation or yearly review with the nurse. Furthermore, our highly deprived sample prioritised day-by-day living over longer term planning, thereby focusing on health in the short term.

Denial or avoidance of long term health outcomes

Most participants were sceptical of the link between lung cancer and smoking. Conversely, participants thought that lung cancer was inevitable due to their current or former lifestyle, including smoking history, working conditions and the reported incidence of lung cancer in their community. Beliefs about inevitability were often coupled with highly negative fearful and fatalistic beliefs about lung cancer, with no cure and eventual death. Such claims were evidenced by a high proportion of friends and family who were diagnosed with lung cancer and often died. A few participants discussed that a cure for lung cancer involved luck or was 'some miracle' (male, 56, Wales, occasional smoker), reflecting a perceived lack of control over early detection and treatment. Consequently, actual or anticipated medical help seeking for lung cancer symptoms was motivated by pain, or to seek a diagnosis and prognosis to notify family members. However, some participants would anticipate refusal of treatment or even contemplate suicide.

For peer review only - http://bmjopen.bmj.com/site/about/guidelines.xhtml

For peer review only

We found differences in how participants with and without dependent family responded to symptoms of lung cancer. Female participants with dependent children or grandchildren were all motivated to visit the doctor with symptoms suggestive of lung cancer, in order to receive a prognosis to enable childcare arrangements after death. Women with dependent children who held more positive beliefs about lung cancer treatment would seek help for treatment to 'stay healthy' and prolong life. Participants with no dependent family would be more likely to ignore lung cancer symptoms, or seek medical help if in pain but refuse treatment.

The model patient

Participants discussed a sense of lack of entitlement to health services due to smoking habit, where respiratory-type symptoms of lung cancer were perceived as self-inflicted. For some, this was reinforced by an actual or expected 'smoking lecture' each time they sought medical help; the lecture made participants feel ostracised, particularly when smoking was used as a coping mechanism and contributed to not feeling worthy of seeking medical help. Some participants perceived that they may be treated differently by health professionals because they live in an area of deprivation, and discussed a potential power imbalance during consultations.

Conversely, participants were highly critical of people who were perceived to waste, exploit and overuse NHS resources. They cited drug addicts, illegitimate benefits claimers, older people wanting social interaction, and people with coughs and colds as over users of the health service. Such beliefs may reflect a downward comparison to other more stigmatised service users to legitimise their own help seeking. In order to be considered a model and non-problem patient, participants legitimise their own help seeking only when absolutely necessary - and often after trying their 'own cures' i.e. cough medicine from the pharmacist - to not burden the doctors. Infrequent attenders or 'good service users' felt a sense of superiority for being a model patient.

Relationship with the healthcare professional

Some participants disclosed traumatic events in their lives including physical and sexual abuse, leading to tobacco dependence and alcohol addiction. In addition, more than half of the sample described symptoms of depression and anxiety. Therefore, the relationship with their healthcare professional was important when considering whether to present with lung symptoms; to feel

For peer review only - http://bmjopen.bmj.com/site/about/guidelines.xhtml

understood by the healthcare professional, with their personal history taken into account in the context of health behaviour such as smoking.

Those who felt comfortable, safe and not judged by their chosen healthcare professional felt encouraged to present with symptoms. Some participants were prepared to wait up to three weeks for an appointment with their preferred healthcare professional to discuss worrisome and potentially serious symptoms that could indicate lung cancer. Many participants reported problems with maintaining continuity of care, highlighting problems with the stretched National Health Service.

[insert Figure 1 here, currently on an additional file]

Focus groups

Two public focus groups were conducted in Wales and England, respectively. Most participants were female and former smokers, and all participants were diagnosed with a lung condition. Two professional focus groups were conducted in Wales. Most participants were female, and were medical professionals (Table 2).

Table 2. Focus group characteristics

Members of the public	N participants	Healthcare professionals and community partners	N participants
**Group 1, England**	**total n=7**	**Group 3, Wales**	**total n=5**
Gender		*Gender*	
Female	6	Female	2
Male	1	Male	3
Smoking status		*Occupation*	
Current smoker	3	Community nurse	1
Former smoker	3	Support group facilitator	1
Never smoker	1	Community partner	1
Self-reported lung condition		Third sector representative	1
COPD	7	Public health representative	1
Recruited through Primary Care and community groups		Recruited through the Health Board	
**Group 2, Wales**	**total n=9**	**Group 4, Wales**	**total n=7**
Gender		*Gender*	
Female	5	Female	6
Male	4	Male	1
Smoking status		*Occupation*	
Current smoker	3	Practice manager	1

For peer review only - http://bmjopen.bmj.com/site/about/guidelines.xhtml

For peer review only

Former smoker	4	Pharmacist	1
Never smoker	2	GP	2
Self-reported lung condition		Practice nurse	2
COPD	9	Medical student	1
Recruited through community groups		Recruited through the Health Board/ Primary Care	

Key themes discussed were: barriers to early lung cancer detection, and preferences regarding the format and content of an intervention for the early detection of lung cancer. See Figure 2 for illustrative quotes.

Barriers to lung cancer symptom presentation

The focus groups confirmed our interview findings, where fear of wasting the doctor's time with trivial symptoms and fear of being judged or lectured about smoking deterred medical help seeking for lung cancer symptoms. In addition, the health professional group supported our findings that patients with lung conditions tend to fixate on chest infections. However, we found disparity between the patient-reported experience of the GP's approach to smoking and the healthcare professional reported approach to smoking cessation. Healthcare professionals in Wales discussed new guidance that discourages health professionals from 'lecturing' patients, suggesting the patient reported experience may be based on previous healthcare interactions, and they consequently anticipate a lecture.

Potential format of an intervention to support earlier lung cancer detection

All groups discussed a preference for community based interventions, away from a traditional healthcare setting, for example a community event, talk in a community venue or health check bus, similar to breast screening mobile units. The anonymous and relaxed nature of such an intervention meant that intervention participants would feel they were not wasting GP time; rather it would act as a signal that their attendance at the event was desired. Participants compared this to a visit to the doctor, where they felt they were wasting the GP's time because they were not invited to attend. It was considered important that the intervention facilitator was knowledgeable or trained, non-judgemental, easy to talk to and approachable. Participants suggested a nurse, pharmacist, trained patient representative or community worker.

For peer review only - http://bmjopen.bmj.com/site/about/guidelines.xhtml

Intervention content

The public groups requested more information about the symptoms of lung cancer. However, the healthcare professional groups felt that current lung cancer symptom information was too broad, leading to dismissal and potentially denial of lung cancer information because people with smoking history or comorbid lung conditions experience most of the symptoms daily. To overcome this problem, the healthcare professionals groups discussed the need for more specific symptom information, emphasising changes to normal symptoms and coupled with information about risk factors for lung cancer.

To modify negative beliefs about lung cancer, the health professionals groups suggested using positive stories to communicate messages about the importance of lung cancer early diagnosis and highlight the potential for survival outcomes with early stage detection.

The inclusion of smoking cessation information in a lung cancer intervention was considered important by all groups. However, the manner in which smoking cessation could be approached was discussed as key to effective promotion of smoking cessation. Participants suggested highlighting the benefits of stopping smoking in a gentle and relaxed manner to encourage choice to quit.

[insert Figure 2 here, currently on an additional file]

DISCUSSION

Our study was the first to explore the influences on lung cancer symptom presentation and intervention preferences in high risk, highly deprived groups across three nations of the UK. We found evidence from the interviews and focus groups that individuals who are high risk for lung cancer fixate on maintaining health in the short term. Prioritising the daily management of their lung condition led to avoidance of longer term health problems such as lung cancer, to gain a sense of control over health in the context of difficult personal circumstances. Health beliefs were found to underpin behaviour in relation to medical help seeking, where perceptions of 'inevitable but curable' chest infections led to immediate help seeking. However, 'inevitable but incurable' lung cancer led to inaction when faced with potentially serious symptoms and anticipated refusal of treatment. Interview participants felt that the relationship with the healthcare professional was key when considering medical help seeking. The importance of the interaction between provider and patient was mirrored in the focus groups, where participants felt that a non-judgemental intervention

For peer review only - http://bmjopen.bmj.com/site/about/guidelines.xhtml

facilitator was important. Multi-faceted community-based interventions, away from the traditional healthcare setting, were preferred by participants.

A major strength of this study was the rigorous sampling procedures. We screened postcodes to ensure participants resided in the lowest quintile of deprivation, and measured multiple additional indicators of deprivation. Individual and area level indicators confirm that our sample was highly deprived, for instance most were unemployed and seeking benefits, and rented social housing. In addition, we recruited participants with no previous diagnosis of lung cancer, without mention of lung cancer until discussed by participants during the interview, or at the end of the interview. These recruitment and interview procedures meant we were able to explore actual and anticipated lung cancer symptom presentation in those who were symptomatic or asymptomatic. This strategy was employed to overcome the methodological limitations associated with studying either retrospective or anticipated symptom presentation in isolation[15]. Although we carefully sampled participants and collected additional demographic measures to validate our sampling frame, we were unable to conduct a focus group in Scotland due to low response, which is a potential limitation of the study. In addition, focus group participants were recruited opportunistically, with the potential that participants were more favourably disposed to an intervention.

Previous empirical studies report prolonged lung cancer symptom presentation due to misattribution[5,13,15-26,33,38,39] and in our study, we found evidence that participants normalised their lung cancer symptoms to smoking habit, and lung and other comorbid conditions. In contrast to previous studies that report haemoptysis as a facilitator to prompt medical help seeking[13,25,27,40-42], participants with experience of haemoptysis reported prolonged medical help seeking or described avoidant coping, and normalisation when blood was noticed. Denial and normalisation of haemoptysis may be specific to socioeconomically deprived groups. Our highly deprived sample reported daily struggles with complex physical and mental health needs, and with the challenges associated with living on no or limited income. Previous studies in socioeconomically deprived communities report that in the context of competing life demands, health was dealt with reactively and with low priority[43,44].

In addition, fear of being ineligible for treatment due to lifestyle, has the potential to deter medical help seeking in deprived groups, conceptualised as Candidacy[44]. The underlying concept of health service Candidacy[44] may explain why participants described feeling unworthy of seeking medical help. In addition to challenging life circumstances, interview and focus group participants reported

For peer review only - http://bmjopen.bmj.com/site/about/guidelines.xhtml

fear of being judged and ignored by health professionals due to their smoking habit or social standing, contributing to perceptions of unworthiness. Furthermore, participants reported the desire to be a model patient and to not waste valuable GP time, which influenced medical help seeking. Although the desire to be a 'good citizen' has previously been reported[24,25], to our knowledge, the current study was the first to explore perceptions of appropriate consultation behaviour in a highly deprived sample. Our emerging findings related to Candidacy, combined with the desire to exhibit 'good' consultation behaviour, may contribute to normalisation of symptoms previously regarded as serious and therefore discourage help seeking. Consequently, high risk, highly deprived participants are likely to focus on health in the short term, and ignore longer-term health issues which may lead to advanced stage lung cancer diagnosis.

We found that participants held seemingly contradictory views of their lung cancer susceptibility, reporting scepticism about the causal role of smoking in lung cancer alongside perceived inevitability of lung cancer. Beliefs about the link between smoking and lung cancer may reflect societal stigma towards smoking, where participants downplay the negative effects of smoking, possibly to legitimise medical help seeking for symptoms considered related to smoking. Perceived inevitability of lung cancer is likely to reflect the high proportion of people that our sample of deprived participants knew with lung cancer, as a consequence of high lung cancer incidence in areas of deprivation. Perceived inevitability for lung cancer has previously been reported to minimise normalisation of lung cancer symptoms and prompt help seeking[45]. However, we report normalisation and ignoring of haemoptysis, possibly due to a combination of high fear and fatalism of lung cancer, difficult life circumstances and low perceived health service Candidacy. Furthermore, our findings raise questions of perceived social justice as influences on medical help seeking. High-risk individuals who believe that they cannot legitimately seek medical help because of their former or current lifestyle may therefore be resigned to the prospect of developing lung cancer.

Practice and policy implications

With a comorbid lung condition and smoking history, those who are high risk for lung cancer will, in the main, be symptomatic. Therefore, as suggested by the focus groups, it is important to highlight the significance of changing and multiple symptoms in an intervention, to avoid normalisation. High risk individuals should be empowered to seek timely medical help and made to feel welcome, not judged or blamed for their current or former lifestyle. For instance, as suggested in the focus groups, interventions targeted at deprived groups could be conducted outside of a traditional healthcare

For peer review only

setting and out to the community with non-judgemental facilitators. Our findings highlight the importance of an intervention where participants would be invited to attend, as opposed to the patient attending the GP surgery. An invitation may help to eliminate any potential perceptions of wasting GP time and make their attendance feel valued.

Over half of the current sample described mental health problems and/or difficult current or former life circumstances. Intervention developers and HCPs in highly deprived communities should be aware of these wider social and contextual factors, and receive training to safeguard themselves. In addition, facilitators and HCPs should know how and where to appropriately signpost intervention participants who disclose difficult circumstances. Finally, the current UK health system potentially encourages patients with a lung condition to focus on short term management of their condition. GP prescribing of antibiotics and the use of rescue packs may inadvertently reinforce patients to detect and act on symptoms of a chest infection. There is potential that this current standard of care could be adapted to educate and encourage patients with a lung condition to detect symptoms of lung cancer, thereby shifting the focus to longer term health. More research is required to understand how to motivate highly deprived groups to consider health in the long term, while recognising the wider social determinants of health[46].

Conclusion

The challenges of living in an area of deprivation with social exclusion issues, combined with fear of judgement by health professionals, contribute to denial and ignoring of lung cancer symptoms. Multi-faceted community based interventions are required to highlight lung cancer symptoms, the importance of early diagnosis and empower people who are high risk for lung cancer to seek timely medical help.

For peer review only

For peer review only - http://bmjopen.bmj.com/site/about/guidelines.xhtml

Additional information

Ethics approval and consent to participate

The study received ethical approval from Southampton Central- Hampshire A Research Ethics Committee (16/SC/0589). Written informed consent was obtained from all participants who took part in the study.

Consent for publication

Not applicable

Data sharing

Qualitative data is not publicly available for this study to maintain participant confidentiality.

Competing interests

The authors confirm that there are no conflicts of interest.

Funding

This work was supported by Cancer Research UK (grant reference number: C16377/A22034).

Author contributorship

All authors designed the study. GM and JH conducted, coded and analysed the interviews and focus group data. All authors contributed to the interpretation of data. GM drafted the manuscript and all authors contributed to the review and editing of the manuscript. All authors read and approved the final manuscript.

Acknowledgements

We would like to thank everyone who helped with recruitment for this study, and all the participants who took part in the study; without these individuals this study would not have been possible. We would like to thank the Advisory Group (Anthony Byrne, Gareth Collier, Adrian Edwards, Dyfed Huws, David Lewis, Maura Matthews and Fiona Walter) and the study Administrator Lucy Watkins, for their ongoing support and advice throughout the study.

For peer review only - http://bmjopen.bmj.com/site/about/guidelines.xhtml

References

1. Fitzmaurice C, Allen C, Barber RM, *et al.* Global, Regional, and National Cancer Incidence, Mortality, Years of Life Lost, Years Lived With Disability, and Disability-Adjusted Life-years for 32 Cancer Groups, 1990 to 2015: A Systematic Analysis for the Global Burden of Disease Study. *JAMA oncol* 2017;**3**(4):524-48. doi: 10.1001/jamaoncol.2016.5688.

2. CRUK. Lung Cancer Survival Statistics. *Cancer Research UK*. 2018. Avaliable at: http://www.cancerresearchuk.org/health-professional/cancer-statistics/statistics-by-cancer-type/lung-cancer#heading-Two [accessed 04.06.2018]

3. McPhail S, Johnson S, Greenberg D, *et al.* Stage at diagnosis and early mortality from cancer in England. *BJC* 2015;**112**(1):108-15. doi: 10.1038/bjc.2015.49.

4. Barrett J, Hamilton W. Pathways to the diagnosis of lung cancer in the UK: a cohort study. *BMC Med Prac*. 2008;**9**(31). doi: 10.1186/1471-2296-9-31.

5. Mitchell ED, Rubin G, Macleod U. Understanding diagnosis of lung cancer in primary care: qualitative synthesis of significant event audit reports. *BJGP* 2013;**63**(606):37-46. doi: 10.3399/bjgp13X660760.

6. Lyratzopoulos G, Neal RD, Barbiere J, *et al*. Variation in number of general practitioner consultations before hospital referral for cancer: findings from the 2010 National Cancer Patient Experience Survey in England. *Lancet Oncol* 2012;**13**(4):353-65. doi: 10.1016/S1470-2045(12)70041-4.

7. Iyen-Omofoman B, Tata L, Baldwin DR, *et al*. Using socio-demographic and early clinical features in general practice to identify people with lung cancer earlier. *Thorax* 2012;**68**:451-9.

8. Hippsley-Cox J, Coupland C. Identifying patients with suspected lung cancer in primary care: derivation and validation of an algorithm. *BJGP* 2011;**61**(592):715-23. doi: 10.3399/bjgp11X606627.

9. NICE. NICE guidelines for lung cancer: diagnosis and management. *NICE*. 2015. Avaliable at: https://www.nice.org.uk/guidance/cg121 [accessed 04.06.2018].

10. National Cancer Registration and Analysis Service. Cancer by deprivation in England: 1996-2011. 2014. Avaliable at: http://www.ncin.org.uk/about_ncin/cancer_by_deprivation_in_england [accessed 04.06.2018].

11. Sekine Y, Katsura H, Koh E, *et al. Early* detection of COPD is important for lung cancer surveillance. *Eur Resp J* 2012;**39**(5):1230-40. doi: 10.1183/09031936.00126011.

12. Malhotra J, Malvezzi M, Negri E, *et al*. Risk factors for lung cancer worldwide. *Eur Resp J* 2016;**48**:899-902. doi: 10.1183/13993003.00359-2016.

For peer review only - http://bmjopen.bmj.com/site/about/guidelines.xhtml

13. Smith SM, Campbell NC, MacLeod U *et al*. Factors contributing to the time taken to consult with symptoms of lung cancer: a cross-sectional study. *Thorax* 2009;**64**(6):523-31.

14. Friedemann-Smith C, Whitaker K, Winstanley K, Wardle J. Smokers are less likely than non-smokers to seek help for a lung cancer 'alarm' symptom. *Thorax* 2016;**71**(7). doi.org/10.1136/thoraxjnl-2015-208063

15. Brouselle A, Breton M, Benhadj L *et al*. Explaining time elapsed prior to cancer diagnosis: patients' perspectives. *BMC Health Serv Res* 2017;**17**(1):448. doi: 10.1186/s12913-017-2390-1.

16. McLachlan S, Mansell G, Sanders T *et al*. Symptom perceptions and help-seeking behaviour prior to lung and colorectal cancer diagnoses: a qualitative study. *Fam Pract*. 2015;**32**(5):568-77. doi: 10.1093/fampra/cmv048.

17. Caswell G, Seymour J, Crosby V *et al*. Lung cancer diagnosed following an emergency admission: exploring patient and carer perspectives on delay in seeking help. *Sup Care Cancer* 2017;**25**(7):2259-66. doi: 10.1007/s00520-017-3633-8.

18. Brindle L, Pope C, Corner J, *et al*. Eliciting symptoms interpreted as normal by patients with early-stage lung cancer: could GP elicitation of normalised symptoms reduce delay in diagnosis? Cross-sectional interview study. *BMJ Open* 2012;**2**(6):1977. doi.org/10.1136/bmjopen-2012-001977.

19. Lyratzopoulos G, Vedsted P, Singh H. Understanding missed opportunities for more timely diagnosis of cancer in symptomatic patients after presentation. *BJC* 2015;**112**(1):84-91. doi: 10.1038/bjc.2015.47.

20. Emery JD, Walter FM, Gray V *et al.* Diagnosing cancer in the bush: a mixed-methods study of symptom appraisal and help-seeking behaviour in people with cancer from rural Western Australia. *Fam Pract* 2013;**30**(5):541-50. doi: 10.1093/fampra/cms087.

21. Birt L, Hall N, Emery J *et al*. Responding to symptoms suggestive of lung cancer: a qualitative interview study. *BMJ Open* 2014;**11**(1);doi: 10.1136/bmjresp-2014-000067.

22. Chatwin J, Sanders C. The influence of social factors on help-seeking for people with lung cancer. *Eur J Cancer Care*. 2013;**22**(6):709-13. doi: 10.1111/ecc.12078.

23. Carter-Harris L. Lung Cancer Stigma as a Barrier to Medical Help-Seeking Behavior: Practice Implications. *J Am Assoc Nurse Pract* 2015;**27**(5):240-5. doi: 10.1002/2327-6924.12227.

24. Tod A, Craven J, Allmark P. Diagnostic delay in lung cancer: a qualitative study. *J Adv Nurs* 2008;**61**(3):336-43. doi: 10.1111/j.1365-2648.2007.04542.x.

25. Tod A, Joanne R. Overcoming delay in the diagnosis of lung cancer: a qualitative study. *Nurs Standard* 2010;**24**(31):34-43. doi: 10.7748/ns2010.04.24.31.35.c7690.

26. Corner J, Hopkinson J, Roffe L. Experience of health changes and reasons for delay in seeking care: a UK study of the months prior to the diagnosis of lung cancer. *Thorax* 2006;**60**:1382-91.

For peer review only - http://bmjopen.bmj.com/site/about/guidelines.xhtml

27. Braybrook DE, Witty KR, Robertson S. Men and lung cancer: a review of the barriers and facilitators to male engagement in symptom reporting and screening. *J Men's Health* 2011;**8**(2):93-9. https://doi.org/10.1016/j.jomh.2011.03.002.

28. Chapple A, Ziebland S, McPherson A. Stigma, shame, and blame experienced by patients with lung cancer: qualitative study. *BMJ* 2004;328:1470. doi.org/10.1136/bmj.38111.639734.7C.

29. Murray SA, Kutzer Y, Habgood E *et al*. Reducing barriers to consulting a General Practitioner in patients at increased risk of lung cancer: a qualitative evaluation of the CHEST Australia intervention. *Fam Pract* 2017;**34**(6):740-6. doi.org/10.1093/fampra/cmx057.

30. Marlow LA, Waller J, Wardle J. Does lung cancer attract greater stigma than other cancer types? *Lung Cancer* 2015;88(1):104-7. doi: 10.1016/j.lungcan.2015.01.024.

31. Quaife S, McEwen A, James S, Wardle J. Smoking is associated with pessimistic and avoidant beliefs about cancer: results from the International Cancer Benchmarking Partnership. *BJC*. 2015 **112**(11):1799-804. doi: 10.1038/bjc.2015.148.

32. Quaife S, Marlow L, McEwen A, Wardle J. Attitudes towards lung cancer screening in socioeconomically deprived and heavy smoking communities: informing screening communication. *HEX* 2017;**20**(4):563-73. doi: 10.1111/hex.12481.

33. Mor V, Masterson-Allen S, Goldberg R, *et al*. Pre-diagnostic symptom recognition and help seeking among cancer patients. *J Community Health* 1990;**15**(4):253-66.

34. Tong A, Sainsbury P, Craig J. Consolidated criteria for reporting qualitative research (COREQ): a 32-item checklist for interviews and focus groups. *Int J Quality Health Care* 2007;**19**(6):349-57.

35. Leventhal H, Brissette I, Leventhal E. The common-sense model of self-regulation of health and illness. In: The Self-regulation of Health and Illness Behaviour. *Routledge*. 2003:42-65.

36. Francis JJ, Johnston M, Robertson C *et al*. What is an adequate sample size? Operationalising data saturation for theory-based interview studies. *Psych Health* 2010;**25**(10):1229-45. doi: 10.1080/08870440903194015.

37. Ritchie LD, Lewis J. Qualitative Research Practice: A Guide for Social Science Students and Researchers. Sage: London. 2003.

38. Hall SE, Holman CD, Threlfall *et al.* Lung Cancer: An exploration of patient and general practitioner perspectives on the realities of care in rural Western Australia. *Aus J Rural Health* 2008;**16**:355-62. doi: 10.1111/j.1440-1584.2008.01016.x.

39. Anderson R, Paarup B, Vedsted P, *et al.* 'Containment' as an analytical framework for understanding patient delay: a qualitative study of cancer patients' symptom interpretation processes. *Soc Sci Med* 2010;**71**(2):378-85. doi: 10.1016/j.socscimed.2010.03.044.

For peer review only - http://bmjopen.bmj.com/site/about/guidelines.xhtml

40. Chan W, Clark A, Dernedde U *et al*. Symptoms, delay to presentation and survival in lung cancer. *Thorax* 2016;**71**(3):80-1.

41. Corner J, Hopkinson J, Fitzsimmons D, *et al*. Is late diagnosis of lung cancer inevitable? Interview study of patients' recollections of symptoms before diagnosis. *Thorax* 2005;**60**:314-9. doi.org/10.1136/thx.2004.029264.

42. Dobson C, Russell A, Brown S, Rubin G.The role of social context in symptom appraisal and help-seeking among people with lung or colorectal symptoms: A qualitative interview study. *Eur J Cancer Care* 2018;**27**(2):e12815. doi: 10.1111/ecc.12815.

43. Smits S, McCutchan G, Wood F *et al*. Development of a Behavior Change Intervention to Encourage Timely Cancer Symptom Presentation Among People Living in Deprived Communities Using the Behavior Change Wheel. *Annals Beh Med*. 2016;**52**(6):474-488. doi: 10.1007/s12160-016-9849-x.

44. Dixon-Woods M, Cavers D, Agarwal S *et al*. Conducting a critical interpretive synthesis of the literature on access to healthcare by vulnerable groups. *BMC Med Res Method* 2006;**6**(35). doi.org/10.1186/1471-2288-6-35.

45. Macdonald S, Watt G, Macleod U. In search of the cancer candidate: can lay epidemilogy help? *Soc Health Illness* 2013;**35**(4):575-91. doi: 10.1111/j.1467-9566.2012.01513.x.

46. Barton H, Grant M. A health map for the local human habitat. *J Royal Soc Promotion Health.* 2006;**126**(6):252-3.

<u>Figure legends</u>

Figure 1. Illustrative quotes (qualitative interviews)

Figure 2. Illustrative quotes (focus groups)

For peer review only - http://bmjopen.bmj.com/site/about/guidelines.xhtml

Figure 1. Illustrative quotes (qualitative interviews)

Theme	Quote
**Symptom detection strategies and help seeking**	
Friends and family notice symptoms	*"My daughter might [notice changes to symptoms] cos she mentions it now and then…she'll give me a dig and she'll say 'your breathing's annoying me'. Cos it's heavy breathing so. Then again there's something wrong"* (Male, 48, Scotland, former smoker)
Sophisticated symptom detection strategies/ monitoring of chest infections	*"If [phlegm is] white and bubbly it's not a chest infection. It's only when it goes green so you can tell yourself exactly how close you are to getting an infection… There's just two different kinds of green spittle, if it's fluorescent green then you've got an infection, normal antibiotics won't work with me, if it's the lighter green I'm fine with that one… it's handy to look out for, because you can get the right medication at the right time…because if anything happens to me, there's no one for my kids."* (Female, 48, Scotland, current smoker)
Normalisation of haemoptysis	*"Coughing up blood, I do actually get some of that I don't know why, but it could be because of the ulcer thing and that…There again then well I do get like nosebleeds, and then I'm thinking the blood maybe coming inside and coming down, you swallow it see. So then that will come back up won't it"* (Male, 62, Wales, former smoker)
**Focus on maintaining health in the short term**	*"I get worried about having chest infection, I get more worried about today or tomorrow rather than the future. The future that's going ahead for us anyway. Lung cancer's not an issue really"* (male, 50, Scotland, former smoker)
Fear of bad news during a consultation	*"I'm very poor in asking questions cos I don't want to know the results. Simple as that…no I don't ask when they say the oxygen [saturation] is alright I just think well it's alright and it's one thing less I haven't got to worry about"* (Female, 69, Wales, former smoker)
**Denial and avoidance of long term health outcomes**	
Scepticism about the link between smoking and lung cancer	*"You hear occasions where people who don't smoke, who've never smoked. Well how do they get their lung cancer?…I've got [lung cancer] in my head, I'm probably going to get it, if I haven't already got it because of the lifestyle I've had. Where I've worked and everything else, what I've worked with."* (Male, 68, England, current smoker)
Perceived inevitability of lung cancer/ anticipate suicide	*"[Lung cancer] is really, really on the forefront on the mind…I just think 'oh god, please don't let me get cancer'…I think if I was to get cancer, I've sometimes said to myself, I'd commit suicide. I would take a pill or something."* (Female, 81, Scotland, current smoker)
	"[Lung cancer] worries me but I've got proper problems to worry

For peer review only

Avoidance of lung cancer due to social and contextual factors	*about [carer for disabled son, problems with social services and benefits claims, insecurity of current council owned housing and problems with area of residence with 'junkies']. I won't worry about it until it's actually here. If I started worrying about eventualities I'd never get anywhere*" (Female, 48, Scotland, current smoker)
Lung cancer fatalism/ anticipated refusal of treatment	*"Until anything happened and I'm actually told that I've got [lung cancer], there's nothing I can do about it. I'm really a believer of what's in your cards is already written. So I don't look at anything like that…But if they told me it was cancer, I would go ok then, but I wouldn't take any of the treatments... if it's my time, it's my time. It just doesn't, I don't think I've got any more fight in me for all that. I think that would be the last straw for me. So I just live every day as it comes now, I don't really plan much. So I'm just living in the day, you know. Cos whatever happens, happens anyway."* (Female, 49, Scotland, current smoker)
Response to lung cancer symptom/ female with dependent family	*"I don't think they can treat [lung cancer]. You've just got to accept it haven't you…I would go to the doctor [with a symptom], I think I would like to know how long I had. Not for me but for [my son] you know. For him…If it was just me I wouldn't want to know, but because I've got him, [I would] definitely…When I seen the blood I did think to myself, I flushed it away right away…I seen the blood and I thought no, and I thought I've got to, you know, because of [my son]. The only way I would want to know is because of him. If I was by myself I would just say, don't want to know…Can't just think about myself I've got to think about him as well."* (Female, 68, Scotland, current smoker)
**The model patient**<br>Perception of healthcare professionals attitude to smokers	*"You feel as though you're an alien because you smoke, you feel as so they just look at you and say 'urghh', you know*" (Female, 52, Scotland, current smoker)
Critical of people who waste NHS resources	*"I can guarantee if I went this Monday and go next Monday the same people are sitting there. I'm being honest, they're a drain on society on the NHS, but that's the way they live…these people that go there are not really ill, I think they're just seeking attention"* (Male, 78, England, current smoker).
**Relationship with healthcare professional**<br>Disclosure of highly sensitive personal problem	*"Some people are friendly and not stony faced…if [the HCP] can't even start a conversation with the simplest of ice breakers then how can people tell about pooping themselves when they're coughing up*" (Female, 48, Scotland, current smoker)
Good relationship with GP	*"I'm alright with [one GP], you could tell her anything, I've shocked her sometimes*" (Female, 51, England, current smoker)

For peer review only

Figure 2. Illustrative quotes (focus groups)

Theme	Quote
**Barriers to lung cancer symptom presentation**<br>Fixation on chest infections	*"People tend to be fixated on a [chest] infection and they want their next rescue pack ready cos almost as if it's inevitable; it's going to happen in the next month or so."* (Focus group 4)
Difference in perception around healthcare professional approach to patients' smoking	*"I think there is a gulf between what people believe their GP would say to them if they do actually talk about [smoking] as opposed to what that conversation actually is in reality….But certainly as far as the formal training coming out of public health, if they are doing that then there is, that's not a lecture…But that's what people fear is going to be what they're going to be told"* (Focus group 3)
**Potential format of an intervention to support earlier lung cancer detection**	*Participant 2: "So what I'm saying is, you know them mobile buses… in the shopping area, where people go shops, or outside the hospital… So they set them up and people are walking past, and even though they can't be bothered to go to the doctors, and they look and they think I'll just pop in*<br>*Participant 1: Cos you wouldn't hesitate you know, you'd just go in.*<br>*Participant 2: You're just a person, they don't know and they're just seeing what's there, or what's there or what's the problem with you. If there's no problem*<br>*Participant 3: People think you don't want to think you're, feel as if you're wasting the doctor's time"* (Focus group 1)
**Intervention content**<br>More specific symptom advice	*Participant 1: "Yeah I think when you say 'cough' it's a bit broad and it's a bit…You know, you've had a cough for two weeks, off you go.*<br>*Participant 3: It'd be useful if it was a change in your regular cough"* (Focus group 4)
Messages to combat negative beliefs	*"Positive messages, particularly around lung cancer because everybody, you know it's like a death knell isn't it? And actually it's not, it doesn't have to be. You know you're talking here about early diagnosis which is a big deal isn't it"* (Focus group 3)
Smoking cessation	*"You've got to include [smoking cessation information]…I think it's how you deliver the message…not in such a way you feel ashamed for smoking. I've noticed [the nurse] has got a way of telling patients how to stop smoking, she does it in a, not in a 'well you should stop smoking', that kind of way. She'll say 'have you ever thought about giving it up. You know it would improve your chest a bit'. And I've seen [the nurse do it] more in a non-lecturey basis, more of a, 'have you ever thought about it?' Relaxed, warmer manner. So I'm not lecturing you, it's your choice. You know it's bad for you."* (Focus group 4)

For peer review only

For peer review only

Supplementary File 1. **Consolidated criteria for reporting qualitative studies (COREQ): 32-item checklist with page numbers to indicate section of the article [34]**

Checklist item	Questions to consider	Page number in article
**Domain 1: Research team and reflexivity**		
Personal Characteristics		
1. Interviewer/facilitator	Which author/s conducted the interview or focus group?	8
2. Credentials	What were the researcher's credentials? *E.g. PhD, MD*	8
3. Occupation	What was their occupation at the time of the study?	8
4. Gender	Was the researcher male or female?	8
5. Experience and training	What experience or training did the researcher have?	8
Relationship with participants		
6. Relationship established	Was a relationship established prior to study commencement?	6
7. Participant knowledge of the interviewer	What did the participants know about the researcher? e.*g. personal goals, reasons for doing the research*	6
8. Interviewer characteristics	What characteristics were reported about the interviewer/facilitator? e.g. *Bias, assumptions, reasons and interests in the research topic*	8
**Domain 2: study design**		
Theoretical framework		
9. Methodological orientation and Theory	What methodological orientation was stated to underpin the study? *e.g. grounded theory, discourse analysis, ethnography, phenomenology, content analysis*	8
Participant selection		
10. Sampling	How were participants selected? *e.g. purposive, convenience, consecutive, snowball*	5-6
11. Method of approach	How were participants approached? e.*g. face-to-face, telephone, mail, email*	6-7
12. Sample size	How many participants were in the study?	9, 13
13. Non-participation	How many people refused to participate or dropped out? Reasons?	9
Setting		
14. Setting of data collection	Where was the data collected? e.*g. home, clinic, workplace*	7
15. Presence of non-participants	Was anyone else present besides the participants and researchers?	7
16. Description of sample	What are the important characteristics of the sample? *e.g. demographic data, date*	9-10, 13-14
Data collection		
17. Interview guide	Were questions, prompts, guides provided by the authors? Was it pilot tested?	6-7

For peer review only

18. Repeat interviews	Were repeat interviews carried out? If yes, how many?	6
19. Audio/visual recording	Did the research use audio or visual recording to collect the data?	6, 8
20. Field notes	Were field notes made during and/or after the interview or focus group?	8
21. Duration	What was the duration of the interviews or focus group?	7
22. Data saturation	Was data saturation discussed?	7
23. Transcripts returned	Were transcripts returned to participants for comment and/or correction?	
**Domain 3: analysis and findings**		
Data analysis		
24. Number of data coders	How many data coders coded the data?	8
25. Description of the coding tree	Did authors provide a description of the coding tree?	8
26. Derivation of themes	Were themes identified in advance or derived from the data?	8
27. Software	What software, if applicable, was used to manage the data?	8
28. Participant checking	Did participants provide feedback on the findings?	8
Reporting		
29. Quotations presented	Were participant quotations presented to illustrate the themes / findings? Was each quotation identified? e.g. *participant number*	Figure 1, Figure 2
30. Data and findings consistent	Was there consistency between the data presented and the findings?	9-15
31. Clarity of major themes	Were major themes clearly presented in the findings?	10-15
32. Clarity of minor themes	Is there a description of diverse cases or discussion of minor themes?	10-15

For peer review only

Supplementary file 1. Symptom sorting task

**Symptoms included in the symptom sorting task**<br>Participants were asked '*We know that people decide go to the doctor at different times with symptoms. I'm going to show you some pieces of card with symptoms on. I'd like you to rank them from the ones that you would go to the doctor with first through to the last on this sheet of paper*.'
Coughing up blood
Persistent chest infection
Chest or shoulder pain
Ache or pain when coughing or breathing
Persistent breathlessness
A cough that doesn't go away
A long standing cough that gets worse
Persistent tiredness or lack of energy
Loss of appetite or unexplained weight loss
Changes in the appearance of fingers such as the ends becoming curved or larger
Hoarse voice

For peer review only

BMJ Open

Engaging high-risk groups in early lung cancer detection: a qualitative study of symptom presentation and intervention preferences amongst the UK's most deprived communities

Journal:	*BMJ Open*
Manuscript ID	bmjopen-2018-025902.R1
Article Type:	Research
Date Submitted by the Author:	06-Nov-2018
Complete List of Authors:	McCutchan, GM; Cardiff University, Division of Population Medicine<br>Hiscock, Julia; Bangor University, North Wales Centre for Primary Care Research<br>Hood, Kerenza<br>Murchie, Peter; University of Aberdeen, Division of Applied Health Science<br>Neal, Richard; University of Leeds<br>Newton, Gareth; Cardiff University, Division of Population Medicine, Patient and Public Involvement<br>Thomas, Sara; Public Health Wales, Cwm Taf Public Health Team<br>Thomas, Ann Maria; Cardiff University, Division of Population Medicine, Patient and Public Involvement<br>Brain, Kate; Cardiff University, Division of Population Medicine, School of Medicine
**Primary Subject Heading**:	Oncology
Secondary Subject Heading:	Respiratory medicine, Qualitative research, Public health, Health policy, Smoking and tobacco
Keywords:	PRIMARY CARE, PUBLIC HEALTH, QUALITATIVE RESEARCH, RESPIRATORY MEDICINE (see Thoracic Medicine), lung cancer, socioeconomic status

SCHOLARONE™
Manuscripts

Title page

Title: Engaging high-risk groups in early lung cancer detection: a qualitative study of symptom presentation and intervention preferences amongst the UK's most deprived communities

Authors: Dr Grace M McCutchan[1$], Dr Julia Hiscock[2], Prof Kerenza Hood[3], Prof Peter Murchie[4], Prof Richard D Neal[5], Mr Gareth Newton[1], Mrs Sara Thomas[6], Mrs Ann-Maria Thomas[1], Prof Kate Brain[1]

Affiliations:

[1]Division of Population Medicine, School of Medicine, Cardiff University, Cardiff, UK

[2]North Wales Centre for Primary Care Research, Bangor University, Wrexham, UK

[3]Centre for Trials Research, School of Medicine, Cardiff University, Cardiff, UK

[4]Division of Applied Health Sciences, Section of Academic Primary Care, University of Aberdeen, Aberdeen, UK

[5]Leeds Institute of Health Sciences, Faculty of Medicine and Health, University of Leeds, Leeds, UK

[6]Cwm Taf Public Health Team, Public Health Wales, Keir Hardy University Health Park, Merthyr Tydfil, UK.

[$]**Corresponding author:** Grace McCutchan. Postal address: Division of Population Medicine, Cardiff University, 1st Floor, Neuadd Meirionnydd, Heath Park, Cardiff, CF14 4YS. Email: mccutchanGM@cardiff.ac.uk. Telephone: +44 (0)2920 687639.

Word count: 5500

For peer review only - http://bmjopen.bmj.com/site/about/guidelines.xhtml

Abstract

Objectives

People at high-risk for lung cancer - current/former smokers, aged 40+ years, with serious lung comorbidity (i.e. Chronic Obstructive Pulmonary Disease) and living in highly deprived areas - are more likely to delay symptom presentation. This qualitative study aimed to understand the influences on early presentation with lung cancer symptoms in high-risk individuals and intervention preferences.

Methods

Semi-structured qualitative interviews with 37 high-risk individuals (without a cancer diagnosis), identified through seven GP practices in socioeconomically deprived areas of England, Scotland and Wales (most deprived 20%). A symptom attribution task was used to explore lung symptom perception and help-seeking, developed using Leventhal's Common Sense Model. Four focus groups with 18 high-risk individuals and 16 local stakeholders (healthcare professionals and community partners) were conducted to explore preferences for an intervention to promote early lung cancer symptom presentation. Data were synthesised using Framework analysis.

Results

Individual and area level indicators of deprivation confirmed that interview participants were highly deprived. Preoccupation with managing 'treatable' short-term conditions (chest infections), led to denial and avoidance of 'inevitable and incurable' longer-term conditions (lung cancer). Feeling judged and unworthy of medical help because of their perceived social standing or lifestyle deterred medical help seeking, particularly when difficult life circumstances and traumatic events led to tobacco and alcohol addiction. Focus group participants recommended multi-faceted interventions in community venues, with information about lung cancer symptoms and the benefits of early diagnosis, led by a trained and non-judgemental facilitator.

Conclusions

This study was novel in engaging a high-risk population to gain an in-depth understanding of the broader contextual influences on self-management of lung cancer symptoms. Perceived lack of health service entitlement and complex lives facilitated avoidance of recognising and presenting with lung cancer symptoms. Community-based interventions have the potential to empower disadvantaged populations to seek medical help for lung symptoms.

For peer review only

Key words: Lung cancer, deprived, qualitative, early diagnosis, medical help seeking, barriers.

Strengths and limitations of the study

- This was the first study to use in-depth qualitative methods to explore how to engage high risk individuals from socioeconomically deprived areas in early lung cancer detection.
- A major strength of this study was the proactive and rigorous sampling procedures used to ensure that our sample was at high risk for lung cancer.
- Assessment of individual and area level indicators of deprivation confirmed that interview participants were highly deprived; all participants resided in the 20% most deprived areas of the three UK nations, and most participants were unemployed/seeking benefits and/or rented social housing.
- To overcome the methodological limitations associated with studying anticipated or retrospectively recalled cancer symptom presentation, we recruited participants with no previous diagnosis of lung cancer and did not mention lung cancer in the interview study materials or during completion of the symptom attribution task.
- Opportunistic recruitment of focus group participants who may have been more favourably disposed to an intervention was a potential study limitation.

For peer review only

INTRODUCTION

Lung cancer is the leading cause of cancer mortality worldwide [1]. Outcomes are among the poorest for all cancers, with only 13% of lung cancer patients surviving five or more years in the UK [2]. Diagnosis of lung cancer at an early stage can enable curative surgical resection, meaning over 80% of patients will survive one year or more when diagnosed at Stage I[3]. Delayed medical help seeking for symptoms and the high proportion of lung cancer diagnoses through emergency departments may partly explain why lung cancer is commonly diagnosed at an advanced, incurable stage[4]. Due to low specificity of lung cancer symptoms and similarity to other acute and comorbid respiratory conditions, patients face difficulty in knowing when to seek medical help[5,6].

Multiple symptoms and risk factors for lung cancer including older age, smoking, the presence of a lung comorbidity and socioeconomic deprivation increase the likelihood that a patient presenting to their GP with symptoms indicative of lung cancer will receive a cancer diagnosis[7-9]. Lung cancer is more common and mortality higher in areas of high socioeconomic deprivation; it has been estimated that each year, socioeconomic inequalities account for 11,700 excess cases of lung cancer and 9,900 potentially avoidable lung cancer deaths in England[10]. High prevalence of smoking, lung comorbid conditions and asbestos exposure, all of which are well documented risk factors for lung cancer, contribute to high lung cancer incidence and mortality in deprived communities[11, 12].

The presence of lung comorbidity such as chronic obstructive pulmonary disease (COPD) and history of smoking have been associated with a lower likelihood of presenting with lung cancer symptoms early[13,14]. In the lead up to lung cancer diagnosis, vague symptoms may go unnoticed or not considered a legitimate symptom to seek medical attention for, or be misattributed to smoking, aging or other comorbid conditions such as heart disease or COPD, thereby prolonging help seeking[5,13,15-26]. In addition, stigma attached to lung cancer[23,27-30] and fear of lung cancer diagnosis can deter medical help seeking for symptoms, particularly among smokers[23-25,29,31-33], leading to advanced stage disease at diagnosis[19]. To date, research has mainly been conducted with lung cancer patients from a range of socioeconomic groups with varying levels of lung cancer risk, retrospectively exploring the barriers to symptom presentation. Evidence is lacking about how individuals who are at high risk, and without a diagnosis of lung cancer, attribute potential lung cancer symptoms and decide to seek medical help.

Strategies to prompt earlier help seeking for lung cancer symptoms are required. However, evidence is limited regarding optimal methods for promoting earlier detection through interventions targeted

For peer review only - http://bmjopen.bmj.com/site/about/guidelines.xhtml

at high risk, highly deprived groups. Mass media[34] and community based social marketing[35] lung cancer campaigns report limited reach to the most deprived groups. A nurse-led primary care intervention for older adults with a long smoking history or recent cessation reported increased and sustained intentions to seek help with lung cancer symptoms[36]. However, the intervention was not targeted at highly deprived groups. Novel methods to support the high risk groups to engage in early lung cancer detection are required.

The current study used a combination of interviews and focus groups to explore potential barriers to early lung cancer detection and strategies to encourage early help seeking with individuals who are the high risk for lung cancer. Qualitative interviews were used to gain an in-depth understanding of the processes and motivations involved in symptom attribution and medical help seeking for potential lung cancer symptoms in high risk, highly deprived individuals. We targeted socioeconomically deprived areas across three nations of the UK to approach potential participants, and used rigorous sampling procedures to ensure that our sample were at the high risk for lung cancer. To overcome methodological limitations associated with retrospective recall, we recruited participants with no previous diagnosis of lung cancer, and framed the interview around lung health, rather than lung cancer. Findings from the interviews were presented to focus groups participants in order to facilitate discussion about preferences and acceptability of interventions to engage high risk, highly deprived groups in early lung cancer detection. The focus groups were conducted in highly deprived areas with stakeholders who lived or worked in these communities. To our knowledge, this was the first study to explore the influences on early lung cancer detection and intervention preferences targeted at high risk groups living in the most deprived areas of the UK.

METHODS

The Consolidated Criteria for Reporting Qualitative Research (COREQ)[37] criteria were used to guide reporting (Supplementary File 1).

Participant recruitment and sampling

Interviews. Thirty-seven interview participants were recruited through seven primary care general medical practices (GP) in South Wales (Cwm Taf: three practices), England (Liverpool: one practice) and Scotland (Aberdeen: three practices). Using routinely published index of multiple deprivation (IMD) data for England, Scotland and Wales, GP practices with the highest proportion of their patients that reside in the most deprived quintile were contacted. Practice managers were asked to screen GP practice databases purposively for eligible study participants: men and women over the

For peer review only - http://bmjopen.bmj.com/site/about/guidelines.xhtml

age of 40, who were current or former smokers, with a lung condition (COPD including chronic bronchitis and emphysema, interstitial lung disease or occupational lung disease). Participants were initially recruited from GP practices in Cwm Taf, where practice managers were asked to screen databases for current and former smokers, with no parameter set for number of years since quit attempt. Due to an initially high response rate from former smokers in Cwm Taf, subsequent participants in Aberdeen and Liverpool were sampled purposively according to smoking history. One GP practice in Aberdeen was asked to recruit current smokers and recent quitters (within ten years). Two GP practices in Aberdeen and one GP practice in Liverpool recruited current smokers only.

To ensure that participants from highly deprived areas were invited to take part in the study, individual postcodes were screened by the research team. Eligible patients from the initial database screen were assigned a pseudo-anonymised participant identifier (PID). PID and postcode were checked against IMD score, and those that resided in the most deprived IMD quintile were eligible for the study. The final list of potentially eligible participants was checked by the GP for ability to provide informed consent, considered by the GP to be a risk to the interviewer or themselves and general health status (i.e. very seriously ill). Participants were excluded if they were diagnosed with lung cancer, were terminally ill or did not have capacity to consent.

Focus groups. Eighteen participants for the focus groups with members of the public were recruited opportunistically through primary care or local community groups. Participant recruitment through primary care employed the same methods as those used to invite the interview participants. Pseudonymised participant identifiers were checked to ensure that those who took part in the focus groups had not already participated in the interviews. Additional participants were recruited opportunistically through local community respiratory support groups and non-health related groups in the local community centre. Local community group organisers in areas of high deprivation were contacted and asked for help to recruit members of the public in our target group. Local health service planning groups and health board staff facilitated recruitment of 12 participants for the healthcare professional and community partner (HPCP) focus groups.

Study procedures

Written consent and permission to audio-record were obtained on the day of the interviews and focus groups.

For peer review only - http://bmjopen.bmj.com/site/about/guidelines.xhtml

Interviews. Eligible participants were invited by letter with more detailed study information attached, with a reminder at two weeks to non-respondents. Those who returned the study reply slip via a FREEPOST envelope were contacted by the interviewer (GM or JH) to arrange a suitable time and date for the interview, to outline the study and answer any questions.

Interviews were conducted using a semi-structured topic guide to facilitate a discussion about illness perceptions and coping strategies; development was guided by the Common Sense Model[38] (Supplementary File 2). The interview aimed to explore experiences of their lung condition, symptom attribution, symptom experience and help seeking behaviour, the influence of smoking history on new or changing symptoms, and if appropriate, lung cancer awareness and beliefs.

A symptom sorting task was used to provide participants with a concrete visual task to increase engagement with the interview in the context of potential low literacy. The task formed a basis for discussion about symptom attribution and experience, where participants were asked to order 11 symptoms from those they would go to the doctor with first, through to the last. The 11 symptoms were selected from the NICE guidance for referral of suspected lung cancer (https://www.nice.org.uk/guidance/cg121). The symptoms were re-worded to simplify the language in line with wording found on the NHS Choices website for lung cancer symptoms and any reference to time scale of symptoms was removed (Supplementary File 2, p.9). For example, a cough that lasts for 3 weeks or more was amended to ‘persistent cough’, and haemoptysis was amended to ‘coughing up blood’. The presentation order of the symptoms was rotated between interviews.

To explore potential lung cancer symptom attribution outside of a cancer context, there was no mention of cancer in the interview study information packs or when participants completed the symptom sorting task. If appropriate, participants were asked questions to explore lung cancer awareness and beliefs at the end of the interview or when participants discussed lung cancer unprompted.

Demographic data were collected using a short questionnaire, including three additional measures of socioeconomic group: age, gender, smoking status (quantity and duration), home ownership, occupation and educational attainment. Interviews were conducted until data saturation (no new themes emerging[39]).

For peer review only - http://bmjopen.bmj.com/site/about/guidelines.xhtml

Focus groups. High risk members of the public, and healthcare professionals (i.e. GP, nurse, community pharmacist/community partners working in areas of deprivation with people with smoking history and/or lung conditions were sent information about the study and invited to take part in the focus group. Focus group participants were explicitly informed that the study was about the development of an intervention about lung cancer. A mutually convenient time, date and location for the focus groups was agreed. The focus groups were conducted using a semi-structured topic guide to explore preferences for an intervention to promote earlier lung cancer diagnosis. Separate topic guides were used for the public and professional groups (Supplementary file 3 and 4). Participants were given a verbal summary of the key findings from the qualitative interviews, and asked to discuss preferences for a potential lung cancer intervention targeted at high risk, highly deprived individuals. Topics for discussion were: preferred format of an intervention, recommendations for intervention content, preferred location and facilitator for intervention delivery, and recommendations for the inclusion of smoking cessation advice.

Setting

Most interviews (n=34) took place face-to-face in participant's own homes, with three taking place in a café, local community centre or over the telephone, and lasted between 46 and 146 minutes (mean 83 minutes). Family members were present for three interviews but did not participate in the study. Focus groups took place in primary care settings (n=2) or local community centres (n=2). Members of the public who took part in the interviews or focus groups were given a £10 shopping. Healthcare professionals and community partners were not reimbursed for their time.

Interviews and focus groups in England were conducted by JH (PhD), a trained and experienced female qualitative Research Fellow and Medical Sociologist. The Welsh and Scottish interviews and focus groups were conducted by GM (PhD), a female Health Psychologist and trained qualitative Research Associate.

Data analysis

Interviews and focus groups were audio-recorded and transcribed verbatim. Anonymised transcripts were analysed in detail using the Framework method[40]. Framework analysis is a well-respected and commonly used approach to qualitative data analysis. It was considered particularly suitable for this study due to its transparency and the team work involved[41]. Framework enabled the sharing of synthesised data charts among team members to facilitate participation in analysis and interpretation workshops.

For peer review only - http://bmjopen.bmj.com/site/about/guidelines.xhtml

The data were analysed in five stages: familiarisation, identification of a thematic framework, indexing, charting, and interpretation. A separate index was created on Microsoft Excel for the interview and focus group data; however, wherever possible, overlap was coded using the same indexing terms, for example 'barriers to symptom presentation' was commonly discussed in both the interview and focus groups. The index was developed by two researchers (GM and JH). Themes were generated independently and consolidated through discussion in nine interpretation workshops over a nine month period by GM and JH. The different perspectives of the researchers as noted above was a benefit during analysis and interpretation. Field notes were recorded for each interview and focus group, and incorporated into discussion during the analysis workshops. Although not formally incorporated into the analysis plan, the positioning of each symptom in the attribution task was considered during interpretive workshops. Interpretive themes were generated by JH and GM, and developed with all authors in monthly management meetings. Transcripts and study findings were not checked by participants; however, all participants were mailed a summary of the study findings.

Ethical approval

The study received ethical approval from Southampton Central- Hampshire A Research Ethics Committee (16/SC/0589).

Patient and public involvement. Patient and public representatives (AMT and GN) were involved in the design of the study and interpretation of study findings in monthly management group meetings. All study materials and topic guides were developed with lay input (AMT and GN) and written to a reading age of 10 years due to potentially low literacy. Reading age was calculated using the Automated Readability Index (www.readabilityformulas.com).

RESULTS

Interviews

Of the 397 invited to take part in the study, 78 people returned the study reply slip and declined to participate in the study; reasons for refusal were unknown. Thirty-seven participants agreed to take part in the study. The majority of the sample were female, current smokers, and with a mean age of 65 years (Table 1). Most had a diagnosis of COPD. All 37 participants resided in the lowest quintile of deprivation for their respective country, of whom 15 were in the most deprived decile. Most

For peer review only

1 participants had left school before age 15 with no formal qualifications, lived in social housing, and
2 claimed disability benefit or job seekers allowance.

3 **Table 1. Qualitative interview sample characteristics**

**Sample characteristics**	**Total n=37**
**Gender**	
Male	16
Female	21
**Age, years**	
Mean (range)	64.7 (48-84)
**Smoking status**	
Current smoker	18
Occasional smoker	3
Former smoker, recent quitter (within five years)	5
Former smoker (quit over five years ago)	11
**Deprivation decile**	
Welsh Index of Multiple Deprivation (WIMD)	
Decile 1 (most deprived 10%)	5
Decile 2 (most deprived 11-20%)	10
Scottish Index of Multiple Deprivation (SIMD)	
Decile 1 (most deprived 10%)	4
Decile 2 (most deprived 11-20%)	12
English Index of Multiple Deprivation (IMD)	
Decile 1 (most deprived 10%)	6
**Self-reported lung condition**	
COPD	26
Chronic bronchitis	2
Chronic emphysema	2
Occupational lung disease	1
Unsure of diagnosis	4
Missing	2
**Educational attainment**	
Left school at/before age 15	29
Completed CSEs, O-Levels or equivalent	5
Completed A levels or equivalent	1
Completed further education but not degree	1
Missing	1
**Employment**	
Employed full-time	2
Employed part-time	1
Casual work	1
Job seekers or disability benefit	17
Retired	16
**Home/living arrangement**	
Own flat/house	14
Rent from local authority/housing association	21
Rent privately	1
Missing	1

4

For peer review only

Key themes were: strategies involved in symptom detection and help seeking behaviour, maintaining short term health, denial and avoidance of longer term health, the desire to be a model patient, and the importance of the relationship with their healthcare professional. See Table 2 for illustrative quotes.

Symptom detection strategies and help seeking

Symptoms discussed during the task were viewed as "part and parcel" (male, 68, England, current smoker) of their lung condition, other pre-existing comorbidities or smoking habit, and were consequently normalised and perceived not to require medical help. Changes to vague or respiratory-type lung cancer symptoms were only taken seriously when remarked on by friends and family or when they impacted on daily life.

Symptoms that could indicate a chest infection were reportedly constantly monitored. Participants discussed using sophisticated strategies such as noticing changes in the colour and consistency of their phlegm or subtle audible changes in their cough to actively detect chest infections. Such strategies were considered important to facilitate early detection and treatment for chest infections through their primary care provider or with rescue packs (emergency packs of steroids and antibiotics that can be kept at home), due to lung condition.

Constant monitoring of phlegm for control of lung condition meant that participants could and would notice haemoptysis, but few reported actively looking for haemoptysis on a regular basis. Disparity between actual and anticipated medical help seeking was reported for haemoptysis. Most participants had not previously experienced haemoptysis, but would anticipate seeking medical help immediately due to the potentially serious nature of blood. However, some participants who had previously or were currently experiencing haemoptysis attributed the presence of blood to non-cancer causes such as their stomach ulcer or a previous flu jab. One participant ascribed their cough to lung cancer. Some of the participants with experience of haemoptysis did not seek medical help.

Focus on maintaining short term health

Participants reported seeking medical help quickly when symptoms were easy to detect, were attributed to what was perceived as a treatable cause and represented an immediate health threat i.e. a chest infection due to lung condition. Participants could often request an appointment the

For peer review only - http://bmjopen.bmj.com/site/about/guidelines.xhtml

same day as permitted by their GP surgery policies. Prompt help seeking was reportedly due to fear of not being able to breathe and the potentially life-threatening nature of chest infections, and is likely to reflect the need to maintain good health in the short term.

The focus on maintaining short term health may reflect low general expectations of health, where some participants disclosed surprise at living beyond 60 years of age. In addition, due to fear of potentially hearing bad news, some participants expressed a preference to not ask questions during a consultation or yearly review with the nurse. Participants discussed prioritising day-by-day living over longer term planning, thereby focusing on health in the short term.

Denial or avoidance of long term health outcomes

Most participants discussed scepticism about the link between lung cancer and smoking. Conversely, participants thought that lung cancer was inevitable due to their current or former lifestyle, including smoking history, working conditions, their lung condition and the reported incidence of lung cancer in their community. For many participants, the topic of lung cancer arose spontaneously. Lung cancer was discussed in the context of perceived inevitability when reflecting on their general lung health and during completion of the symptom task when recalling friends/family with lung cancer. Beliefs about inevitability were often coupled with highly negative fearful and fatalistic beliefs about lung cancer, with no cure and eventual death. Such claims were evidenced by knowing a high proportion of friends and family who were diagnosed with lung cancer and often died. A few participants discussed that a cure for lung cancer involved luck or was 'some miracle' (male, 56, Wales, occasional smoker), reflecting a perceived lack of control over early detection and treatment. Consequently, actual or anticipated medical help seeking for lung cancer symptoms was motivated by pain, or to seek a diagnosis and prognosis to notify family members. However, some participants anticipated refusal of treatment or would even contemplate suicide.

We found differences in how participants with and without dependent family reported responding to symptoms of lung cancer. Female participants with dependent children or grandchildren discussed a motivation to visit the doctor with symptoms suggestive of lung cancer, in order to receive a prognosis to enable childcare arrangements after death. Women with dependent children who held more positive beliefs about lung cancer treatment reported the need to seek help for treatment to 'stay healthy' and prolong life. Participants with no dependent family were more likely to ignore lung cancer symptoms, or anticipate seeking medical help if in pain but refuse treatment.

For peer review only - http://bmjopen.bmj.com/site/about/guidelines.xhtml

The model patient

Participants discussed a sense of lack of entitlement to health services due to smoking habit, where respiratory-type symptoms of lung cancer were perceived as self-inflicted. For some, this was reinforced by an actual or expected 'smoking lecture' each time they sought help from healthcare professionals; the lecture made participants feel ostracised, particularly when smoking was used as a coping mechanism and contributed to not feeling worthy of seeking medical help. Some participants perceived that they may be treated differently by health professionals because they live in an area of deprivation, and discussed a potential power imbalance during consultations.

Conversely, participants reported high criticism towards people who were perceived to waste, exploit and overuse NHS resources. They cited drug addicts, illegitimate benefits claimers, older people wanting social interaction, and people with coughs and colds as over users of the health service. Such beliefs may reflect a downward comparison to other more stigmatised service users to legitimise their own help seeking. In order to be considered a model and non-problem patient, participants discussed legitimising their own help seeking by only consulting when absolutely necessary - and often after trying their 'own cures' i.e. cough medicine from the pharmacist - to not burden the doctors. Infrequent attenders or 'good service users' discussed feeling a sense of superiority for being a model patient.

Relationship with the healthcare professional

Some participants disclosed traumatic events in their lives including physical and sexual abuse, leading to tobacco dependence and alcohol addiction. In addition, more than half of the sample described symptoms of depression and anxiety. Therefore, the reported relationship with their healthcare professional was important when considering whether to present with lung symptoms. Participants discussed the need to feel understood and not judged by their healthcare professional, with their personal history taken into account in the context of health behaviour such as smoking.

Those who discussed feeling comfortable, safe and not judged by their chosen healthcare professional felt encouraged to present with symptoms. Some participants reported that they were prepared to wait up to three weeks for an appointment with their preferred healthcare professional to discuss worrisome and potentially serious symptoms that could indicate lung cancer. Many

For peer review only - http://bmjopen.bmj.com/site/about/guidelines.xhtml

participants reported problems with maintaining continuity of care, highlighting problems with the stretched National Health Service.

Table 2. Illustrative quotes (qualitative interviews)

Theme	Quote
**Symptom detection strategies and help seeking**	
Friends and family notice symptoms	*"My daughter might [notice changes to symptoms] cos she mentions it now and then…she'll give me a dig and she'll say 'your breathing's annoying me'. Cos it's heavy breathing so. Then again there's something wrong"* (Male, 48, Scotland, former smoker)
Sophisticated symptom detection strategies/ monitoring of chest infections	*"If [phlegm is] white and bubbly it's not a chest infection. It's only when it goes green so you can tell yourself exactly how close you are to getting an infection… There's just two different kinds of green spittle, if it's fluorescent green then you've got an infection, normal antibiotics won't work with me, if it's the lighter green I'm fine with that one… it's handy to look out for, because you can get the right medication at the right time…because if anything happens to me, there's no one for my kids."* (Female, 48, Scotland, current smoker)
Normalisation of haemoptysis	*"Coughing up blood, I do actually get some of that I don't know why, but it could be because of the ulcer thing and that…There again then well I do get like nosebleeds, and then I'm thinking the blood maybe coming inside and coming down, you swallow it see. So then that will come back up won't it"* (Male, 62, Wales, former smoker)
**Focus on maintaining health in the short term**	*"I get worried about having chest infection, I get more worried about today or tomorrow rather than the future. The future that's going ahead for us anyway. Lung cancer's not an issue really"* (male, 50, Scotland, former smoker)
Fear of bad news during a consultation	*"I'm very poor in asking questions cos I don't want to know the results. Simple as that…no I don't ask when they say the oxygen [saturation] is alright I just think well it's alright and it's one thing less I haven't got to worry about"* (Female, 69, Wales, former smoker)
**Denial and avoidance of long term health outcomes**	
Scepticism about the link between smoking and lung cancer	*"You hear occasions where people who don't smoke, who've never smoked. Well how do they get their lung cancer?…I've got [lung cancer] in my head, I'm probably going to get it, if I haven't already got it because of the lifestyle I've had. Where I've worked and everything else, what I've worked with."* (Male, 68, England, current smoker)
Perceived inevitability of lung cancer/ anticipate suicide	*"[Lung cancer] is really, really on the forefront on the mind…I just think 'oh god, please don't let me get cancer'…I think if I was to get cancer, I've sometimes said to myself, I'd commit suicide. I*

For peer review only - http://bmjopen.bmj.com/site/about/guidelines.xhtml

	would take a pill or something." (Female, 81, Scotland, current smoker)
Avoidance of lung cancer due to social and contextual factors	*"[Lung cancer] worries me but I've got proper problems to worry about [carer for disabled son, problems with social services and benefits claims, insecurity of current council owned housing and problems with area of residence with 'junkies']. I won't worry about it until it's actually here. If I started worrying about eventualities I'd never get anywhere"* (Female, 48, Scotland, current smoker)
Lung cancer fatalism/ anticipated refusal of treatment	*"Until anything happened and I'm actually told that I've got [lung cancer], there's nothing I can do about it. I'm really a believer of what's in your cards is already written. So I don't look at anything like that…But if they told me it was cancer, I would go ok then, but I wouldn't take any of the treatments... if it's my time, it's my time. It just doesn't, I don't think I've got any more fight in me for all that. I think that would be the last straw for me. So I just live every day as it comes now, I don't really plan much. So I'm just living in the day, you know. Cos whatever happens, happens anyway."* (Female, 49, Scotland, current smoker)
Response to lung cancer symptom/ female with dependent family	*"I don't think they can treat [lung cancer]. You've just got to accept it haven't you…I would go to the doctor [with a symptom], I think I would like to know how long I had. Not for me but for [my son] you know. For him…If it was just me I wouldn't want to know, but because I've got him, [I would] definitely…When I seen the blood I did think to myself, I flushed it away right away…I seen the blood and I thought no, and I thought I've got to, you know, because of [my son]. The only way I would want to know is because of him. If I was by myself I would just say, don't want to know…Can't just think about myself I've got to think about him as well."* (Female, 68, Scotland, current smoker)
**The model patient**<br>Perception of healthcare professionals attitude to smokers	*"You feel as though you're an alien because you smoke, you feel as so they just look at you and say 'urghh', you know"* (Female, 52, Scotland, current smoker)
Critical of people who waste NHS resources	*"I can guarantee if I went this Monday and go next Monday the same people are sitting there. I'm being honest, they're a drain on society on the NHS, but that's the way they live…these people that go there are not really ill, I think they're just seeking attention"* (Male, 78, England, current smoker).
**Relationship with healthcare professional**<br>Disclosure of highly sensitive personal problem	*"Some people are friendly and not stony faced…if [the HCP] can't even start a conversation with the simplest of ice breakers then how can people tell about pooping themselves when they're coughing up"* (Female, 48, Scotland, current smoker)

For peer review only - http://bmjopen.bmj.com/site/about/guidelines.xhtml

Good relationship with GP	*"I'm alright with [one GP], you could tell her anything, I've shocked her sometimes"* (Female, 51, England, current smoker)

Focus groups

Two public focus groups were conducted in Wales and England. Most participants were female and former smokers, and all participants were diagnosed with a lung condition. Two professional focus groups were conducted in Wales. Most participants were female, and were medical professionals (Table 3).

Table 3. Focus group characteristics

Members of the public	N participants	Healthcare professionals and community partners	N participants
**Group 1, England**	**total n=7**	**Group 3, Wales**	**total n=5**
Gender		*Gender*	
Female	6	Female	2
Male	1	Male	3
Smoking status		*Occupation*	
Current smoker	3	Community nurse	1
Former smoker	3	Support group facilitator	1
Never smoker	1	Community partner	1
Self-reported lung condition		Third sector representative	1
COPD	7	Public health representative	1
Recruited through Primary Care and community groups		Recruited through the Health Board	
**Group 2, Wales**	**total n=9**	**Group 4, Wales**	**total n=7**
Gender		*Gender*	
Female	5	Female	6
Male	4	Male	1
Smoking status		*Occupation*	
Current smoker	3	Practice manager	1
Former smoker	4	Pharmacist	1
Never smoker	2	GP	2
Self-reported lung condition		Practice nurse	2
COPD	9	Medical student	1
Recruited through community groups		Recruited through the Health Board/ Primary Care	

Key themes discussed were: barriers to early lung cancer detection, and preferences regarding the format and content of an intervention for the early detection of lung cancer. See Table 4 for illustrative quotes.

For peer review only - http://bmjopen.bmj.com/site/about/guidelines.xhtml
For peer review only

Barriers to lung cancer symptom presentation

The public and stakeholder focus groups confirmed our interview findings, where fear of wasting the doctor's time with trivial symptoms and fear of being judged or lectured about smoking was perceived to deter medical help seeking for potential lung cancer symptoms. In addition, the health professional group supported our findings that patients with lung conditions tend to be preoccupied by chest infections. However, we found potential disparity between the patient-reported experience of the GP's approach to smoking and the healthcare professional reported approach to smoking cessation. Healthcare professionals in Wales discussed new guidance that discourages health professionals from 'lecturing' patients, suggesting the patient reported experience may be based on previous healthcare interactions, and they consequently anticipate a lecture. Alternatively, healthcare professionals may be unaware of new guidance, or not adhere to new guidance and consequently continue to 'lecture' patients about smoking.

Potential format of an intervention to support earlier lung cancer detection

All groups discussed a preference for community based interventions, away from a traditional healthcare setting, for example a community event, talk in a community venue or health check bus, similar to breast screening mobile units. The anonymous and relaxed nature of such an intervention meant that intervention participants would feel they were not wasting GP time; rather it would act as a signal that their attendance at the event was desired. Participants compared this to a visit to the doctor, where they discussed a feeling of wasting the GP's time because they were not invited to attend. It was considered important that the intervention facilitator was knowledgeable or trained, non-judgemental, easy to talk to and approachable, highlighting the importance of relational aspects of a lung cancer intervention. Participants suggested a nurse, pharmacist, trained patient representative or community worker.

Intervention content

The public groups requested more information about the symptoms of lung cancer. However, the healthcare professional groups felt that current lung cancer symptom information was too broad, leading to dismissal and potential denial of lung cancer information because people with smoking history or comorbid lung conditions experience most of the symptoms daily. To overcome this problem, the healthcare professionals groups discussed the need for more specific symptom

For peer review only - http://bmjopen.bmj.com/site/about/guidelines.xhtml

information, emphasising changes to normal symptoms and coupled with information about risk factors for lung cancer.

To modify negative beliefs about lung cancer, the health professionals groups suggested using positive stories to communicate messages about the importance of lung cancer early diagnosis and highlight the potential for survival outcomes with early stage detection.

The inclusion of smoking cessation information in a lung cancer intervention was considered important by all groups. However, the manner in which smoking cessation could be approached was discussed as key to effective promotion of smoking cessation. Participants suggested highlighting the benefits of stopping smoking in a gentle and relaxed manner to encourage choice to quit.

Table 4. Illustrative quotes (focus groups)

Theme	Quote
**Barriers to lung cancer symptom presentation**<br>Fixation on chest infections	*"People tend to be fixated on a [chest] infection and they want their next rescue pack ready cos almost as if it's inevitable; it's going to happen in the next month or so."* (Focus group 4)
Difference in perception around healthcare professional approach to patients' smoking	*"I think there is a gulf between what people believe their GP would say to them if they do actually talk about [smoking] as opposed to what that conversation actually is in reality….But certainly as far as the formal training coming out of public health, if they are doing that then there is, that's not a lecture…But that's what people fear is going to be what they're going to be told"* (Focus group 3)
**Potential format of an intervention to support earlier lung cancer detection**	*Participant 2: "So what I'm saying is, you know them mobile buses… in the shopping area, where people go shops, or outside the hospital… So they set them up and people are walking past, and even though they can't be bothered to go to the doctors, and they look and they think I'll just pop in*<br>*Participant 1: Cos you wouldn't hesitate you know, you'd just go in.*<br>*Participant 2: You're just a person, they don't know and they're just seeing what's there, or what's there or what's the problem with you. If there's no problem*<br>*Participant 3: People think you don't want to think you're, feel as if you're wasting the doctor's time"* (Focus group 1)
**Intervention content**<br>More specific symptom advice	*Participant 1: "Yeah I think when you say 'cough' it's a bit broad and it's a bit…You know, you've had a cough for two weeks, off you go.*

For peer review only - http://bmjopen.bmj.com/site/about/guidelines.xhtml

	Participant 3: It'd be useful if it was a change in your regular cough" (Focus group 4)
Messages to combat negative beliefs	*"Positive messages, particularly around lung cancer because everybody, you know it's like a death knell isn't it? And actually it's not, it doesn't have to be. You know you're talking here about early diagnosis which is a big deal isn't it"* (Focus group 3)
Smoking cessation	*"You've got to include [smoking cessation information]…I think it's how you deliver the message…not in such a way you feel ashamed for smoking. I've noticed [the nurse] has got a way of telling patients how to stop smoking, she does it in a, not in a 'well you should stop smoking', that kind of way. She'll say 'have you ever thought about giving it up. You know it would improve your chest a bit'. And I've seen [the nurse do it] more in a non-lecturey basis, more of a, 'have you ever thought about it?' Relaxed, warmer manner. So I'm not lecturing you, it's your choice. You know it's bad for you."* (Focus group 4)

For peer review only

DISCUSSION

Our study was the first to explore the influences on lung cancer symptom presentation in high risk, highly deprived groups across three nations of the UK. Preferences for an intervention targeted at high-risk groups were ascertained through focus groups. We found evidence from the interviews and focus groups that individuals who are at high risk for lung cancer tend to be preoccupied by maintaining health in the short term. Prioritising the daily management of their lung condition led to avoidance of longer term health problems such as lung cancer, to gain a sense of control over health in the context of difficult personal circumstances. Health beliefs were found to underpin behaviour in relation to medical help seeking, where perceptions of 'inevitable but curable' chest infections led to immediate help seeking. However, 'inevitable but incurable' lung cancer led to inaction when faced with potentially serious symptoms and anticipated refusal of treatment. Interview participants felt that the relationship with the healthcare professional was key when considering medical help seeking. The importance of the relational interaction between provider and patient was mirrored in the focus groups, where participants felt that a non-judgemental intervention facilitator was important. Multi-faceted community-based interventions, away from the traditional healthcare setting, were preferred by participants.

Previous empirical studies report prolonged lung cancer symptom presentation due to misattribution[5,13,15-26,33, 42] and in our study, we found evidence that participants normalised their symptoms indicative of lung cancer to smoking habit, and lung and other comorbid conditions. In contrast to previous studies that report haemoptysis as a facilitator to prompt medical help seeking[13,25,27,43-45], participants with experience of haemoptysis reported described avoidant coping, and normalisation when blood was noticed. Denial and normalisation of haemoptysis may be specific to socioeconomically deprived groups. Our highly deprived sample reported daily struggles with complex physical and mental health needs, and with the challenges associated with living on no or limited income. Previous studies in socioeconomically deprived communities report that in the context of competing life demands, health was dealt with reactively and with low priority[46,47].

Fear of being ineligible for treatment due to lifestyle has not been well described in studies with lung cancer patients or those at high risk[44,48]. In contrast, participants in the current study described feeling disentitled to medical services in the context of their lifestyle and circumstances. The underlying concept of health service Candidacy[47] may explain why participants felt unworthy of seeking medical help and is likely to be of particular importance in our highly deprived sample. In addition to challenging life circumstances, interview and focus group participants reported fear of

For peer review only

being judged and ignored by health professionals due to their smoking habit or perceived social standing, contributing to feelings of unworthiness. Participants reported the desire to be a model patient and to not waste valuable GP time, which influenced medical help seeking. Although the desire to be a 'good citizen' has previously been reported[24,25], to our knowledge, the current study was the first to explore perceptions of appropriate consultation behaviour in a highly deprived sample. Our emerging findings related to Candidacy, combined with the desire to exhibit 'good' consultation behaviour, may contribute to normalisation of symptoms previously regarded as serious and therefore discourage help seeking. Consequently, disadvantaged populations are likely to focus on health in the short term, and ignore longer-term health issues which may lead to advanced stage lung cancer diagnosis.

We found that participants held seemingly contradictory views on their lung cancer susceptibility, reporting scepticism about the causal role of smoking in lung cancer alongside perceived inevitability of lung cancer. Beliefs about the link between smoking and lung cancer may reflect societal stigma towards smoking, where participants downplay the negative effects of smoking, possibly to legitimise medical help seeking for symptoms considered related to smoking. Perceived inevitability of lung cancer is likely to reflect high levels of exposure in social networks where there is high incidence and poor outcomes of lung cancer [10] and can to minimise normalisation of lung cancer symptoms and prompt help seeking[49]. However, contrary to previous studies, our sample reported feeling that lung cancer was inevitable, but normalised and ignored haemoptysis, possibly due to a combination of high fear and fatalism of lung cancer, difficult life circumstances and low perceived health service Candidacy. Furthermore, our findings raise questions of perceived social justice as influences on medical help seeking. High-risk individuals who believe that they cannot legitimately seek medical help because of their former or current lifestyle may therefore be resigned to the prospect of developing lung cancer.

A major strength of this study was the rigorous sampling procedure. We screened postcodes to ensure participants resided in the lowest quintile of deprivation, and measured multiple additional indicators of deprivation. Individual and area level indicators confirm that our sample was highly deprived, for instance most were unemployed and seeking benefits, and rented social housing. In addition, we recruited participants with no previous diagnosis of lung cancer, without mention of lung cancer until discussed by participants during the interview, or at the end of the interview. These recruitment and interview procedures meant we were able to explore previous and anticipated lung cancer symptom presentation in those who were symptomatic or asymptomatic. This strategy was

For peer review only - http://bmjopen.bmj.com/site/about/guidelines.xhtml

employed to overcome the methodological limitations associated with studying either retrospective or anticipated symptom presentation in isolation[15]. However, our qualitative study was unable to establish causal links between barriers and help seeking, nor can we generalise or compare the findings to high socioeconomic groups; instead, we conducted an in-depth study to explore how best to engage high risk, highly deprived individuals in early lung cancer detection. Although we carefully sampled participants and collected additional demographic measures to validate our sampling frame, some GP practices were asked to recruit by specific smoking status rather than the whole range of smoking status, potentially introducing bias to our sample. In addition, we were unable to conduct a focus group in Scotland due to low response, which is a potential limitation of the study. Finally, focus group participants were recruited opportunistically, with the potential that participants were more favourably disposed to an intervention.

Practice and policy implications

With a comorbid lung condition and smoking history, those who are high risk for lung cancer will, in the main, be symptomatic. To avoid normalisation, it is important to highlight the significance of changing and multiple symptoms. High risk individuals should be empowered to seek timely medical help and made to feel welcome, not judged or blamed for their current or former lifestyle. For instance, interventions targeted at disadvantaged populations could be conducted outside of the traditional healthcare setting. Our findings highlight the importance of an intervention where participants would be invited to attend, as opposed to presenting to the GP surgery, in order to eliminate concerns about wasting GP time and legitimise their attendance. Community based interventions have the potential to harness the relational aspects of help seeking, through interventions led by non-judgemental and welcoming facilitators. It is possible that previous mass media and social marketing lung cancer awareness interventions report low campaign reach to deprived groups [34,35] in part because they were not designed to motivate help seeking through intensive approaches to build trusting relationships and confidence. More research is required to understand how the relational aspects of help seeking could be operationalised in an intervention.

Over half of the current sample described mental health problems and/or difficult current or former life circumstances. Intervention developers and healthcare professionals in highly deprived communities should be aware of these wider social and contextual factors; they should receive training to recognise such circumstances and know how to appropriately signpost. Finally, we suggest that the current UK health system may encourage patients with a lung condition to focus on

For peer review only

short term management of their condition. GP prescribing of antibiotics and the use of rescue packs (prescribed antibiotics for storage at home in the event of an exacerbation) may inadvertently reinforce patients to detect and act on symptoms of a chest infection[50]. There is potential that this current standard of care could be adapted to educate and encourage patients with a lung condition to detect symptoms of lung cancer, thereby shifting the focus to longer term health. More research is required to understand how to motivate highly deprived groups to consider health in the long term, while recognising the wider social determinants of health[51].

Conclusion

The challenges of living in an area of deprivation with social exclusion issues, combined with fear of judgement by health professionals, contribute to denial and ignoring of lung cancer symptoms. Multi-faceted community based interventions are required to, highlight lung cancer symptoms, the importance of early diagnosis and empower people who are high risk for lung cancer to seek timely medical help.

For peer review only

For peer review only - http://bmjopen.bmj.com/site/about/guidelines.xhtml

Additional information

Ethics approval and consent to participate

The study received ethical approval from Southampton Central- Hampshire A Research Ethics Committee (16/SC/0589). Written informed consent was obtained from all participants who took part in the study.

Consent for publication

Not applicable

Data sharing

Qualitative data is not publicly available for this study to maintain participant confidentiality.

Competing interests

The authors confirm that there are no conflicts of interest.

Funding

This work was supported by Cancer Research UK (grant reference number: C16377/A22034).

Author contributorship

All authors (GMM, JH, KH, PM, RDN, GN, ST, AMT, KB) designed the study. GMM and JH conducted, coded and analysed the interviews and focus group data. All authors contributed to the interpretation of data. GMM drafted the manuscript and all authors contributed to the review and editing of the manuscript. All authors read and approved the final manuscript.

Acknowledgements

We would like to thank everyone who helped with recruitment for this study, and all the participants who took part in the study; without these individuals this study would not have been possible. We would like to thank the Advisory Group (Anthony Byrne, Gareth Collier, Adrian Edwards, Dyfed Huws, David Lewis, Maura Matthews and Fiona Walter) and the study Administrator Lucy Watkins, for their ongoing support and advice throughout the study.

For peer review only - http://bmjopen.bmj.com/site/about/guidelines.xhtml

References

1. Bray F, Ferlay J, Soerjomataram I, Siegel RL, Torre LA, Jemal A. Global cancer statistics 2018: GLOBOCAN estimates of incidence and mortality worldwide for 36 cancers in 185 countries. CA: A Cancer Journal for Clinicians.0(0).

2. Allemani C, Matsuda T, Di Carlo V, Harewood R, Matz M, Nikšić M, et al. Global surveillance of trends in cancer survival 2000–14 (CONCORD-3): analysis of individual records for 37 513 025 patients diagnosed with one of 18 cancers from 322 population-based registries in 71 countries. The Lancet. 2018;391(10125):1023-75.

3. McPhail S, Johnson S, Greenberg D, *et al.* Stage at diagnosis and early mortality from cancer in England. *BJC* 2015;**112**(1):108-15. doi: 10.1038/bjc.2015.49.

4. Barrett J, Hamilton W. Pathways to the diagnosis of lung cancer in the UK: a cohort study. *BMC Med Prac*. 2008;**9**(31). doi: 10.1186/1471-2296-9-31.

5. Mitchell ED, Rubin G, Macleod U. Understanding diagnosis of lung cancer in primary care: qualitative synthesis of significant event audit reports. *BJGP* 2013;**63**(606):37-46. doi: 10.3399/bjgp13X660760.

6. Lyratzopoulos G, Neal RD, Barbiere J, *et al*. Variation in number of general practitioner consultations before hospital referral for cancer: findings from the 2010 National Cancer Patient Experience Survey in England. *Lancet Oncol* 2012;**13**(4):353-65. doi: 10.1016/S1470-2045(12)70041-4.

7. Iyen-Omofoman B, Tata L, Baldwin DR, *et al*. Using socio-demographic and early clinical features in general practice to identify people with lung cancer earlier. *Thorax* 2012;**68**:451-9.

8. Hippsley-Cox J, Coupland C. Identifying patients with suspected lung cancer in primary care: derivation and validation of an algorithm. *BJGP* 2011;**61**(592):715-23. doi: 10.3399/bjgp11X606627.

9. NICE. NICE guidelines for lung cancer: diagnosis and management. *NICE*. 2015. Avaliable at: https://www.nice.org.uk/guidance/cg121 [accessed 04.06.2018].

10. National Cancer Registration and Analysis Service. Cancer by deprivation in England: 1996-2011. 2014. Avaliable at: http://www.ncin.org.uk/about_ncin/cancer_by_deprivation_in_england [accessed 04.06.2018].

11. Sekine Y, Katsura H, Koh E, *et al. Early* detection of COPD is important for lung cancer surveillance. *Eur Resp J* 2012;**39**(5):1230-40. doi: 10.1183/09031936.00126011.

12. Malhotra J, Malvezzi M, Negri E, *et al*. Risk factors for lung cancer worldwide. *Eur Resp J* 2016;**48**:899-902. doi: 10.1183/13993003.00359-2016.

For peer review only

13. Smith SM, Campbell NC, MacLeod U *et al*. Factors contributing to the time taken to consult with symptoms of lung cancer: a cross-sectional study. *Thorax* 2009;**64**(6):523-31.

14. Friedemann-Smith C, Whitaker K, Winstanley K, Wardle J. Smokers are less likely than non-smokers to seek help for a lung cancer 'alarm' symptom. *Thorax* 2016;**71**(7). doi.org/10.1136/thoraxjnl-2015-208063

15. Brouselle A, Breton M, Benhadj L *et al*. Explaining time elapsed prior to cancer diagnosis: patients' perspectives. *BMC Health Serv Res* 2017;**17**(1):448. doi: 10.1186/s12913-017-2390-1.

16. McLachlan S, Mansell G, Sanders T *et al*. Symptom perceptions and help-seeking behaviour prior to lung and colorectal cancer diagnoses: a qualitative study. *Fam Pract*. 2015;**32**(5):568-77. doi: 10.1093/fampra/cmv048.

17. Caswell G, Seymour J, Crosby V *et al*. Lung cancer diagnosed following an emergency admission: exploring patient and carer perspectives on delay in seeking help. *Sup Care Cancer* 2017;**25**(7):2259-66. doi: 10.1007/s00520-017-3633-8.

18. Brindle L, Pope C, Corner J, *et al*. Eliciting symptoms interpreted as normal by patients with early-stage lung cancer: could GP elicitation of normalised symptoms reduce delay in diagnosis? Cross-sectional interview study. *BMJ Open* 2012;**2**(6):1977. doi.org/10.1136/bmjopen-2012-001977.

19. Lyratzopoulos G, Vedsted P, Singh H. Understanding missed opportunities for more timely diagnosis of cancer in symptomatic patients after presentation. *BJC* 2015;**112**(1):84-91. doi: 10.1038/bjc.2015.47.

20. Emery JD, Walter FM, Gray V *et al.* Diagnosing cancer in the bush: a mixed-methods study of symptom appraisal and help-seeking behaviour in people with cancer from rural Western Australia. *Fam Pract* 2013;**30**(5):541-50. doi: 10.1093/fampra/cms087.

21. Birt L, Hall N, Emery J *et al*. Responding to symptoms suggestive of lung cancer: a qualitative interview study. *BMJ Open* 2014;**11**(1);doi: 10.1136/bmjresp-2014-000067.

22. Chatwin J, Sanders C. The influence of social factors on help-seeking for people with lung cancer. *Eur J Cancer Care*. 2013;**22**(6):709-13. doi: 10.1111/ecc.12078.

23. Carter-Harris L. Lung Cancer Stigma as a Barrier to Medical Help-Seeking Behavior: Practice Implications. *J Am Assoc Nurse Pract* 2015;**27**(5):240-5. doi: 10.1002/2327-6924.12227.

24. Tod A, Craven J, Allmark P. Diagnostic delay in lung cancer: a qualitative study. *J Adv Nurs* 2008;**61**(3):336-43. doi: 10.1111/j.1365-2648.2007.04542.x.

25. Tod A, Joanne R. Overcoming delay in the diagnosis of lung cancer: a qualitative study. *Nurs Standard* 2010;**24**(31):34-43. doi: 10.7748/ns2010.04.24.31.35.c7690.

26. Corner J, Hopkinson J, Roffe L. Experience of health changes and reasons for delay in seeking care: a UK study of the months prior to the diagnosis of lung cancer. *Thorax* 2006;**60**:1382-91.

For peer review only - http://bmjopen.bmj.com/site/about/guidelines.xhtml

27. Braybrook DE, Witty KR, Robertson S. Men and lung cancer: a review of the barriers and facilitators to male engagement in symptom reporting and screening. *J Men's Health* 2011;**8**(2):93-9. https://doi.org/10.1016/j.jomh.2011.03.002.

28. Chapple A, Ziebland S, McPherson A. Stigma, shame, and blame experienced by patients with lung cancer: qualitative study. *BMJ* 2004;328:1470. doi.org/10.1136/bmj.38111.639734.7C.

29. Murray SA, Kutzer Y, Habgood E *et al*. Reducing barriers to consulting a General Practitioner in patients at increased risk of lung cancer: a qualitative evaluation of the CHEST Australia intervention. *Fam Pract* 2017;**34**(6):740-6. doi.org/10.1093/fampra/cmx057.

30. Marlow LA, Waller J, Wardle J. Does lung cancer attract greater stigma than other cancer types? *Lung Cancer* 2015;88(1):104-7. doi: 10.1016/j.lungcan.2015.01.024.

31. Quaife S, McEwen A, James S, Wardle J. Smoking is associated with pessimistic and avoidant beliefs about cancer: results from the International Cancer Benchmarking Partnership. *BJC*. 2015 **112**(11):1799-804. doi: 10.1038/bjc.2015.148.

32. Quaife S, Marlow L, McEwen A, Wardle J. Attitudes towards lung cancer screening in socioeconomically deprived and heavy smoking communities: informing screening communication. *HEX* 2017;**20**(4):563-73. doi: 10.1111/hex.12481.

33. Mor V, Masterson-Allen S, Goldberg R, *et al*. Pre-diagnostic symptom recognition and help seeking among cancer patients. *J Community Health* 1990;**15**(4):253-66.

34. Moffat J, Bentley A, Ironmonger L, Boughey A, Radford G, Duffy S. The impact of national cancer awareness campaigns for bowel and lung cancer symptoms on sociodemographic inequalities in immediate key symptom awareness and GP attendances. British Journal Of Cancer. 2015;112:S14.

35. Kennedy MPT, Cheyne L, Darby M, Plant P, Milton R, Robson JM, et al. Lung cancer stage-shift following a symptom awareness campaign. Thorax. 2018.

36. Smith S, Fielding S, Murchie P, Johnston M, Wyke S, Powell R, et al. Reducing the time before consulting with symptoms of lung cancer: a randomised controlled trial in primary care. The British Journal of General Practice. 2013;63(606):e47-e54.

37. Tong A, Sainsbury P, Craig J. Consolidated criteria for reporting qualitative research (COREQ): a 32-item checklist for interviews and focus groups. *Int J Quality Health Care* 2007;**19**(6):349-57.

38. Leventhal H, Brissette I, Leventhal E. The common-sense model of self-regulation of health and illness. In: The Self-regulation of Health and Illness Behaviour. *Routledge*. 2003:42-65.

39. Francis JJ, Johnston M, Robertson C *et al*. What is an adequate sample size? Operationalising data saturation for theory-based interview studies. *Psych Health* 2010;**25**(10):1229-45. doi: 10.1080/08870440903194015.

40. Ritchie LD, Lewis J. Qualitative Research Practice: A Guide for Social Science Students and Researchers. Sage: London. 2003.

For peer review only - http://bmjopen.bmj.com/site/about/guidelines.xhtml

41. Ward D.J., Furber C., Tierney S., Swallow V. (2013) Using Framework Analysis in nursing research: a worked example. *Journal of Advanced Nursing* 69(11), 2423–2431. 10.1111/jan.12127

42. Anderson R, Paarup B, Vedsted P, *et al.* 'Containment' as an analytical framework for understanding patient delay: a qualitative study of cancer patients' symptom interpretation processes. *Soc Sci Med* 2010;**71**(2):378-85. doi: 10.1016/j.socscimed.2010.03.044.

43. Chan W, Clark A, Dernedde U *et al*. Symptoms, delay to presentation and survival in lung cancer. *Thorax* 2016;**71**(3):80-1.

44. Corner J, Hopkinson J, Fitzsimmons D, *et al*. Is late diagnosis of lung cancer inevitable? Interview study of patients' recollections of symptoms before diagnosis. *Thorax* 2005;**60**:314-9. doi.org/10.1136/thx.2004.029264.

45. Dobson C, Russell A, Brown S, Rubin G.The role of social context in symptom appraisal and help-seeking among people with lung or colorectal symptoms: A qualitative interview study. *Eur J Cancer Care* 2018;**27**(2):e12815. doi: 10.1111/ecc.12815.

46. Smits S, McCutchan G, Wood F *et al*. Development of a Behavior Change Intervention to Encourage Timely Cancer Symptom Presentation Among People Living in Deprived Communities Using the Behavior Change Wheel. *Annals Beh Med*. 2016;**52**(6):474-488. doi: 10.1007/s12160-016-9849-x.

47. Dixon-Woods M, Cavers D, Agarwal S *et al*. Conducting a critical interpretive synthesis of the literature on access to healthcare by vulnerable groups. *BMC Med Res Method* 2006;**6**(35). doi.org/10.1186/1471-2288-6-35.

48. Smith SM, Murchie P, Devereux G, Johnston M, Lee AJ, Macleod U, et al. Developing a complex intervention to reduce time to presentation with symptoms of lung cancer. The British Journal of General Practice. 2012;62(602):e605-e15.

49. Macdonald S, Watt G, Macleod U. In search of the cancer candidate: can lay epidemilogy help? *Soc Health Illness* 2013;**35**(4):575-91. doi: 10.1111/j.1467-9566.2012.01513.x.

50. NICE. NICE guidelines for lung cancer: diagnosis and management. *NICE*. 2015. Avaliable at: https://www.nice.org.uk/guidance/cg121 [accessed 04.06.2018].

51. Barton H, Grant M. A health map for the local human habitat. *J Royal Soc Promotion Health.* 2006;**126**(6):252-3.

For peer review only - http://bmjopen.bmj.com/site/about/guidelines.xhtml

Supplementary File 1. Consolidated criteria for reporting qualitative studies (COREQ): 32-item checklist with page numbers to indicate section of the article [34]

**Checklist item**	**Questions to consider**	**Page number in article**
**Domain 1: Research team and reflexivity**		
Personal Characteristics		
1. Interviewer/facilitator	Which author/s conducted the interview or focus group?	8
2. Credentials	What were the researcher's credentials? *E.g. PhD, MD*	8
3. Occupation	What was their occupation at the time of the study?	8
4. Gender	Was the researcher male or female?	8
5. Experience and training	What experience or training did the researcher have?	8
Relationship with participants		
6. Relationship established	Was a relationship established prior to study commencement?	7
7. Participant knowledge of the interviewer	What did the participants know about the researcher? e.*g. personal goals, reasons for doing the research*	7
8. Interviewer characteristics	What characteristics were reported about the interviewer/facilitator? e.g. *Bias, assumptions, reasons and interests in the research topic*	8
**Domain 2: study design**		
Theoretical framework		
9. Methodological orientation and Theory	What methodological orientation was stated to underpin the study? *e.g. grounded theory, discourse analysis, ethnography, phenomenology, content analysis*	8-9
Participant selection		
10. Sampling	How were participants selected? *e.g. purposive, convenience, consecutive, snowball*	5-6
11. Method of approach	How were participants approached? e.*g. face-to-face, telephone, mail, email*	6-7
12. Sample size	How many participants were in the study?	5, 6, 10, 16
13. Non-participation	How many people refused to participate or dropped out? Reasons?	9
Setting		
14. Setting of data collection	Where was the data collected? e.*g. home, clinic, workplace*	8
15. Presence of non-participants	Was anyone else present besides the participants and researchers?	8
16. Description of sample	What are the important characteristics of the sample? *e.g. demographic data, date*	9-10, 16
Data collection		
17. Interview guide	Were questions, prompts, guides provided by the authors? Was it pilot tested?	Appendix 2-4

For peer review only

18. Repeat interviews	Were repeat interviews carried out? If yes, how many?	6
19. Audio/visual recording	Did the research use audio or visual recording to collect the data?	6, 8
20. Field notes	Were field notes made during and/or after the interview or focus group?	8
21. Duration	What was the duration of the interviews or focus group?	8
22. Data saturation	Was data saturation discussed?	7
23. Transcripts returned	Were transcripts returned to participants for comment and/or correction?	
**Domain 3: analysis and findings**		
Data analysis		
24. Number of data coders	How many data coders coded the data?	8
25. Description of the coding tree	Did authors provide a description of the coding tree?	8-9
26. Derivation of themes	Were themes identified in advance or derived from the data?	8-9
27. Software	What software, if applicable, was used to manage the data?	8-9
28. Participant checking	Did participants provide feedback on the findings?	8-9
Reporting		
29. Quotations presented	Were participant quotations presented to illustrate the themes / findings? Was each quotation identified? e.g. *participant number*	14-16, 18-19
30. Data and findings consistent	Was there consistency between the data presented and the findings?	11-19
31. Clarity of major themes	Were major themes clearly presented in the findings?	11-19
32. Clarity of minor themes	Is there a description of diverse cases or discussion of minor themes?	11-19

For peer review only

Supplementary File 2: Interview topic guide

Interview prologue

- Introduce the researcher, ensure the participant is comfortable.
- Explain purpose of the interview:
  - *"We are interested in lung health.*
  - *We would like to know how people cope with lung symptoms and how they decide if they should go to the doctor or not.*
  - *Some of the questions I ask during the interview will be about smoking. I'm not here to tell you to stop smoking; I'm just interested if you have any experiences of smoking.*
  - *I'm not a clinician so I can't give you any advice on symptoms, but I can tell you where you can go to get help"*

- Check understanding of interview purpose, role of researcher, and what will happen in the interview. Give opportunity for questions.
- Partners or family members will be welcome to join the interviews to explore relational influences on lung symptom awareness and help seeking, and the interview topic guide will be adapted accordingly.
- After establishing what is understood about the study, and answering any questions, explain that the interview will be recorded. Obtain consent for the interview and for the recording. If not already done, set up and switch on the recording equipment while the participant signs the consent form.

Note to interviewer: the grey boxes signify the key topics to be explored during the interview. The questions listed below are examples of suggested prompts for each topic. You are not required to read these verbatim unless specified.

For peer review only

Overall aim of the qualitative interview:
-To explore how people with a history of smoking and respiratory conditions interpret and act on new or changing lung symptoms (how people cope with lung symptoms and how they decide to go to the doctor with symptoms)
-To explore the influences of perceived risk, fear, shame, stigma, family and friends on lung symptom presentation

1. Experiences of lung condition
Establish what lung condition the participant has, how the lung condition affects them, the types of symptoms they experience and how they are usually managed.

Rationale: introduce the participant to the format of a qualitative interview and make the participant feels comfortable. Establish details of their lung condition.

Potential prompts:

- Can you tell me about your lung condition?
- Tell me about your experiences with X lung condition.
    - How long have you had X condition?
    - What sort of symptoms do you experience?
    - How long do the symptoms usually last?
- What do you think causes these symptoms?
- How do you usually manage your symptoms?
- How are your symptoms usually managed/ treated by your healthcare professional?
    - How effective do you think this is?

For peer review only

2. Symptom attribution task

Use as a tool for prompting an in-depth understanding of symptom attributions and confidence to interpret new or changing symptoms.
Include discussion around previous symptom experiences including: what action was taken, if and who they sought medical help from. Explore how their lung condition and smoking history might influence symptom attributions (i.e. do these mask symptoms?) and symptom presentation.

Rationale: to explore lung symptom attribution; confidence in recognising and articulating symptoms; previous symptom experience; planning when/how to act on symptoms; influence of smoking history on perceptions of lung symptoms

Ask participant to order symptom cards from symptoms that they would seek medical help quickest for and those they would seek help slowest for (arrow and boxes below will be stretched to A4 size, see page 10). Ask participant to write numbers on the symptom cards. 1= first, 11=last. If they change the order of the symptom, ensure the previous number is crossed out and the new number written on card. Write PID on the back of each symptom card. Take photos of the task.

Say the following phrase verbatim: “We know that people decide go to the doctor at different times with symptoms. I’m going to show you some pieces of card with symptoms on. I’d like you to rank them from the ones that you would go to the doctor with first through to the last on this sheet of paper.”

Cut individual symptom cards (see page 12) for the following symptoms. Please rotate the order:

- Coughing up blood
- A cough that doesn’t go away
- A long standing cough that gets worse
- Pain in your chest or shoulder
- Persistent breathlessness
- Persistent chest infections
- Persistent tiredness or lack of energy
- Loss of appetite or unexplained weight loss
- Ache or pain when breathing or coughing
- A hoarse voice
- Changes in the appearance of your fingers, such as becoming more curved or their ends becoming larger

See the **glossary of terms at the end of the topic guide** with standardised definitions and additional explanation of symptoms. If the participant does not understand what each symptom means, ask them what they think it means, then refer to the glossary of terms.

First to go to the doctor

↑

Last to go to the doctor

If the participant asks the interviewer what these symptoms are, first **ask the participant what they think they are**. If they ask again either suggest to move on and discuss what they could be after the interview (if appropriate) or say the symptoms have been taken from the NHS website for lung cancer.

For peer review only

Note to interviewer: Aim to the complete the ordering of symptoms in the task first, and then ask questions in this section. However, if any of the following (2a, 2b, 2c, 2d) if bought up spontaneously by the participant whilst they are doing the card sorting exercise then explore these issues at that point. Then return to the card sorting exercise, followed by questions in the following section (2a, 2b, 2c, 2d).

Topics and prompts for symptom attribution task

Once the symptoms have been ordered, ask:

- Can you tell me why you put x first?
- Can you tell me why you put x last?

2a. Symptom experience

To understand if the participant has experienced the symptom previously- what did they attribute the symptom to? What did they do? If they have not experienced the symptom before, what would they hypothetically do if they were to experience symptom in the future?

- Which of these symptoms have you had before?
    - What did you think the symptom(s) was/were?
    - Which symptom(s) did you go to the doctor with?
        - Why did you decide to go to the doctor with these symptoms?
        - What did the doctor say?
    - Which symptoms did you decide to not go to the doctor with?
        - Why did you decide not to go to the doctor with these symptoms?
- For the other symptoms we have not talked about, if you developed any of them what would you do?
    - How long do you think it would take you to go to the doctor with these symptoms?
    - Can you tell me why it would take you this amount of time to go to your doctor with this symptom?
    - What you think these symptoms could be?
- Are there any symptoms you wouldn't go to the doctor with?
    - Can you tell me why you wouldn't go to the doctor with these symptoms?

For peer review only

2b. Detecting change

Explore how the participant has/would notice new or changing symptoms and their confidence to detect new and changing symptoms. Ask this section generally, but if the participant is struggling then ask them about confidence to detect new and changing symptoms in the context of previous symptoms experiences. i.e. earlier you mentioned you felt breathless for a long time, how confident did you feel to know that this was a new or changing symptom?

- How would you normally tell if any lung symptoms have changed/ are new/ or unusual for you?
    - What would you do if you think you noticed a change in a symptom?
    - How confident would you feel in recognising a change in your usual symptom(s)?
    - How confident would you feel recognising new symptoms?
    - How would you notice a change in any of these symptoms?

2c. Barriers

Explore the influences of barriers and social influences on lung symptom presentation behavior

- Can you think of anything that would influence your decision to go to the doctor with any of these symptoms?
    - Probe barriers/enablers to going to the GP with a symptom:
        - Transport
        - Long wait times/ appointment policies
        - Worry about wasting the doctors time i.e. Some people have told us that they don't go to the doctor as they worry about wasting the doctors time. What do you think about this?
        - The influence of partner/ social influences (who suggested you go/ don't go to the doctor?)
        - How confident would you feel talking to the doctor about these symptoms?
            - How do you feel when you are talking to the doctor?

2d. Smoking

Explore the influence of smoking habit on the ability to notice new or changing symptoms. If/how smoking habit acts as a barrier to seeking medical help. If vaping comes up say something like- that is really interesting, do you mind if I ask you some questions later about vaping if we have time? If they vape then still explore the influence of past smoking on symptom perceptions.

- Would you expect to have any of the symptoms we have talked about because you smoke or used to smoke?
    - Thinking about smoking, how do you think your [past] smoking would affect how you notice if a symptom has changed or is new/ unusual for you?
    - Does your doctor ever discuss smoking with you?
        - How does this make you feel?

For peer review only - http://bmjopen.bmj.com/site/about/guidelines.xhtml

▪

3. Lung cancer

If appropriate, explore lung cancer knowledge, beliefs about lung cancer and perceived risk here. If bought up spontaneously by the participant earlier in the interview, explore lung cancer then (if appropriate) Confidence to detect a lung cancer symptom. The influences of smoking and perceived risk, shame and associated with lung cancer.

Rationale: to explore lung cancer symptom awareness; to explore perceived risk of lung ca[illegible] emotional consequences such as fear, fatalism [illegible] blame; perceived causes and effectiveness of e[illegible] detection and treatment; the influence of smo[illegible] history on the formation of beliefs about lung [illegible]

If it is OK with you, I'm going to ask you some questions about lung cancer. If you feel uncomfortable with any of my questions, please let me know and we can move on.

Potential prompts:

- Earlier you mentioned some of the symptoms that could be lung cancer. Can you think of any other symptoms that you think might be lung cancer?
- What else do you know about lung cancer?
- Can you tell me a little more about what you think about lung cancer as a disease?
    - Where do you think that feeling comes from?
    - What do you think other people's views are on lung cancer?
- What do you think causes lung cancer? S
    - If yes and appropriate: How does that make you feel as a [past] smoker?
    - If appropriate: do you ever worry about lung cancer because you [used to] smoke?
    - How does that make you feel?
    - Is there anything in particular that makes you think your risk is high or low?
- How confident would you feel in recognising a symptom that could be lung cancer?
    - How does your [past] smoking affect your confidence in recognising a change in your body that could be lung cancer?
- What would you do if you had a symptom that you thought was lung cancer?

For peer review only

4. Social influences

Explore influences of social networks on help seeking behavior and basis of cancer beliefs.

Rationale: to explore social norms, influences and stigma around help seeking; people who they know who have had lung cancer and the effects on beliefs and perceptions; how social networks might influence help-seeking

- If you had a symptom you thought might be lung cancer, would you tell anyone about it?
  - Who would you tell?
  - Why would you tell that person?
  - What do you think they would say to you if you told them about a symptom?
  - Would they encourage/discourage you to visit your doctor?
- Has anyone ever noticed a symptom of yours and suggest that you go to the doctor?
  - Probe: who/ what happened.
- We know that lung cancer isn't as common as other types of cancer like breast cancer. Do you know anyone who has ever been diagnosed with lung cancer?
- Can you tell me about the effects that [person] having lung cancer has had on you?
  - How has this affected your views about lung cancer?

5. Closing questions

Final questions to end on a positive note

Rationale: to end the interview on a positive note

- If you were to offer anyone some advice on lung symptoms, what would it be?
- If the NHS were to make some changes to the services they offer to help people with lung symptoms get seen quicker, what would you suggest they change?
- Can you tell me about a positive experience of going to the doctor?

I know we've talked about a lot of things today, but would you like to tell me about anything else that we haven't talked about?

Debrief

- Summarise the interview and address any questions or concerns.
- Check whether it is ok to contact them if there is anything that needs to be clarified after listening back to the conversation.
- Ensure that they know how to contact us for further help/information/to add further information
- Thank them for their time and give them the gift voucher.
- If the participant discloses symptoms during the interview suggest they seek medical help from their GP. Offer lung cancer leaflet and site specific helpline numbers.
- Have stop smoking service details available if they request it
- Offer to provide a summary of study findings

Helpline numbers

Wales

- Tenovus Cancer Care support line on 0808 808 1010. The support line is open 8am-8pm, 7 days a week. Calls are free from a BT landline.

England and Scotland

- British Lung Foundation helpline on 03000 030555. This helpline is open 9am-5pm Monday to Friday. Calls cost the same as a local call.
- Roy Castle Lung Cancer Foundation helpline on 0333 323 7200. This helpline is open 9am-5pm Monday to Friday. Calls are free from a BT landline.

For peer review only

Symptoms in the symptom task:

**Coughing up blood**
**A cough that doesn't go away**
**A long standing cough that gets worse**
**Pain in your chest or shoulder**
**Persistent breathlessness**
**Persistent chest infections**
**Persistent tiredness or lack of energy**
**Loss of appetite or unexplained weight loss**
**Ache or pain when breathing or coughing**
**A hoarse voice**
**Changes in the appearance of your fingers, such as becoming more curved or their ends becoming larger**

For peer review only

Glossary of terms

Coughing up blood

If you notice that there is some blood coming up when you cough

A cough that doesn't go away

If you have a nagging cough that just doesn't seem to go away

A long standing cough that gets worse

You have had a cough for a long time but you think that it might have got worse

Pain in your chest or shoulder

A sharp or achy pain in either your chest or shoulder, or both

Persistent breathlessness

You feel like you can't catch your breath or become out of breath when doing tasks you used to be fine with

Persistent chest infections

If you have had a few chest infections in a row and they don't seem to be getting better or keep coming back

Persistent tiredness or lack of energy

If you have been feeling tired for a while or just feel like you don't have any energy

Loss of appetite or unexplained weight loss

If you have been loosing weight without trying to and can't explain why or you just haven't been feeling up to eating the amount you normally would for a while

Ache or pain when breathing or coughing

If you have a sharp pain or achy feeling when you cough or breathe

A hoarse voice

A croaky or gravelly voice

Changes in the appearance of your fingers, such as becoming more curved or their ends becoming larger

If you notice that the ends of your fingers have changed shape

Persistent definition

Something that you notice you have had for a while and won't go away.

For peer review only

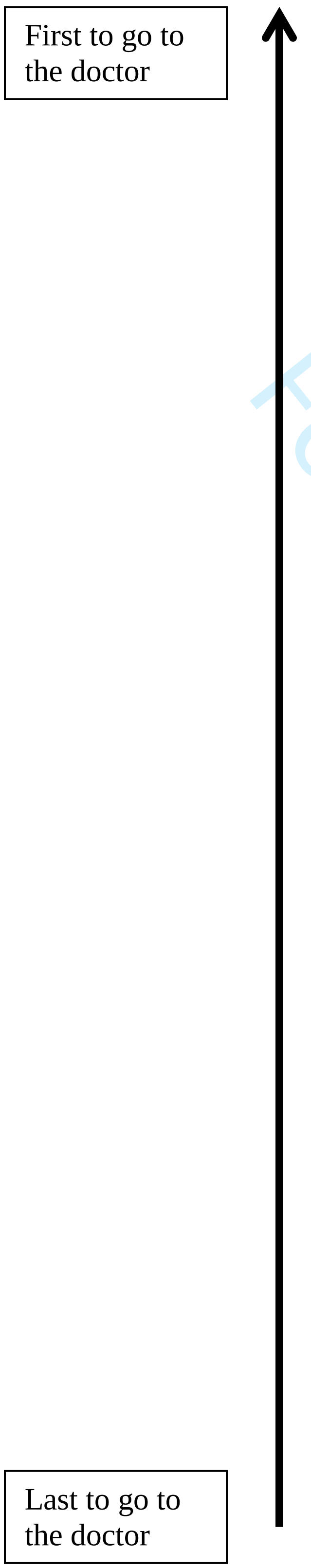

For peer review only

Supplementary File 3: HPCP focus group topic guide

Focus group aim: To explore the needs and preferences from members of the public and local stakeholders (healthcare professionals/ community partners) for an intervention to support earlier lung cancer detection and diagnosis, targeted at high risk, harder to reach groups (over 40's, who are current/former smokers, living in areas of deprivation with serious lung comorbidity i.e. COPD).

Introduction

- Explain the aims of the focus group discussion. Emphasise that the focus group is not a test; we are interested in participants' preferences for an intervention for "lung health" to prompt earlier lung cancer symptom presentation, and how we could access people to take part in an intervention. All comments are welcomed: positive and negative.
    - 'We know that people who are high risk for lung cancer (people over the age of 40, living in deprived areas, who currently smoke or used to smoke and have a lung condition like COPD) are often diagnosed at a late stage, where treatment options are limited and a cure is less likely. This might be because they delay seeking medical advice with symptoms suggestive of lung cancer.

      We are considering if we should develop something that will give people who are high risk for lung cancer information about symptoms and when/ where they should go and seek medical help from.

      As healthcare professionals and people who work in deprived communities, we would like to know what you think about if we should do this. If you think this is a good idea, we would also like to know how best we can access this target group and invite them to take part in an intervention about lung health. Your ideas and suggestions are really valuable to us because of your wealth of experience, so all comments are welcomed (both positive and negative)'.
- Explain the voluntary nature of the study and that the focus group will be recorded with permission. If not already done, set up and switch on the recording equipment while participants sign the consent form.
- Before starting the focus group, remind participants about confidentiality and ask participants not to talk over each other. Go around the circle and ask participants to introduce themselves for the transcription.

For peer review only

Rationale: to explore whether the findings resonate with patients and members of the public in their local community

Part1. Phase 1 interview findings

Discussion of Phase 1 interview findings

Provide a short overview of key findings from Phase 1 interviews regarding barriers and enablers to lung cancer awareness and early symptom presentation in the target group. Ask the group to share their thoughts on the interview findings, and whether they resonate with them.

'We have been interviewing people across the UK who are high risk for lung cancer- people over the age of 40 who have a lung condition like COPD, who currently smoke or used to smoke and live in deprived areas. We wanted to understand how people think about symptoms of lung cancer, how and when people decide to go to the doctor with lung symptoms and the barriers to going to the doctor with these symptoms.

We found that people are completely fixated on detecting chest infections and look out for symptoms of a chest infection most days. They were really good at knowing when they had a chest infection and going to the doctor quickly to get antibiotics because they know it can be treated.

We also found that people tend to deny or ignore health problems that might affect them in the future, like lung cancer so may not go to the doctor with some serious symptoms. We think this is because they think that although lung cancer is inevitable, they also think that it cannot be cured so don't go and see the doctor

We want to develop something that can help people get these important symptoms of lung cancer seen to quicker by a medical professional, but we are not really sure how to do this. We would like to know what you think.'

- How can we do this?
- What do you think of these findings?
- To what extent do these findings resonate with you?

Rationale: to seek views on how to access the target group for an intervention and explore preferences for an intervention to support earlier presentation, including mode of delivery, target group, content and stop smoking information

Preferences on intervention content

- What sorts of things do you think people would like to know / what skills would they like to learn?
  - What do you think would be most useful to people?
  - What information would be the highest priority/most important for them?
  - Should we avoid any information?
  - Do you think it is best to focus on symptoms or health beliefs?

Preferences on intervention format

- How can we support people to seek medical help quickly with lung cancer symptoms? [what format]
  - Probe: a group one-off educational session to promote "lung health" in the local community; a leaflet/DVD; a lung health check; event in the community; posters in the local community
  - Do you think people want education or to learn 'skills for health'?
    - What sort of skills do you think people would like to know?
- Who do you think would be best to lead or facilitate an intervention about lung health/ encourage people to seek medical help quickly with lung cancer symptoms?
  - Probe: patient representative, lay advisor, community partner, healthcare professional (GP, practice nurse, smoking cessation counsellor, respiratory nurse specialist…?)
- When would be best to support people to manage their lung health?
  - What do you think about using the point at which they are diagnosed with a lung condition as a 'teachable moment' to do an intervention about lung cancer?
  - What about during a regular check-up appointment with the nurse i.e. six monthly COPD clinic reviews?
- What do you think about a brief intervention with health care professionals in addition to a public facing intervention?
  - What would be useful to you as healthcare professionals?
- The intention is that this intervention would be implemented across the UK. How do you think everything we have talked about today might need to differ based on where someone lives (i.e. different countries) or the person in the intervention?
  - How could we incorporate an intervention into the different models of care across regions/countries?
- How do you think this intervention could fit with other current or planned health promotion activities?

Accessing intervention participants

- Where could we approach our target group in an intervention about lung cancer/lung health?
- Probe: through community pharmacies, primary care, community nurses, existing groups for people with lung conditions?
  - Probe: on utilising family / social networks to encourage participation of MoP?
- Are you aware of any existing groups for people who fall into our target group?
  - What do you think about "piggybacking" onto these existing community groups or existing care plans with the nurse?
  - How do you think people would react to this?

Smoking cessation

- ‘We know that people really don’t like it when the doctor tells them to stop smoking and some people felt like they were treated differently by the doctors because they smoked. Sometimes this put people off going to the doctor with lung symptoms. On the other hand some people feel that it is only fair to be told to stop smoking. When we design something about lung cancer, we don’t know if we should include something to help people to stop smoking or not.’
- What do you think about including stop smoking information in the intervention?
    - How do you think smokers might react to including stop-smoking information?
    - Can you think of ways we might be able to include stop-smoking information without putting smokers off?
    - What do you think about using signposting to stop smoking services instead of providing information about smoking cessation during the session?
- What do you think about using the intervention to cover things that are not related to lung health i.e. mental health or other factors that could be related to lung health?
    - How should we approach this?

Debrief

‘Thank you for taking part in this study. We hope to use the findings from this focus group to develop an intervention based on the findings from the interviews and your recommendations to encourage earlier lung symptom presentation in those who are high risk for lung cancer. Anything you said will be treated as confidential. The voice-recoding will be stored securely. Any quotes used in published research will not have your name or anything that could identify you. Do you have any questions? [answer any questions] Here are my contact details if you have any further questions.’

For peer review only

Supplementary File 4: Members of the public focus group topic guide

Focus group aim: To explore the needs and preferences from members of the public and local stakeholders for an intervention to support earlier lung cancer detection and diagnosis. The intervention will be targeted at high risk, harder to reach groups (over 40's, who are current/former smokers, living in areas of deprivation with serious lung comorbidity i.e. COPD).

Introduction

- Explain the aims of the focus group. Emphasise that the focus group is not a test; we are interested in participants' preferences for an intervention for "lung health" to prompt earlier lung symptom presentation, and how we could access people to take part in an intervention. All comments are welcomed: positive and negative.
  - *'We know that some people sometimes might take a bit longer to go to the doctor with important lung symptoms. I am thinking about developing something that will highlight important lung symptoms, and encourage people to go to speak to someone who is medically trained about their symptoms. I would like to know what you think about best ways to do this. I would also like to know how you think we can find people for this. Your ideas and suggestions are really valuable to us, so all comments are welcomed (both positive and negative)'*.
- Explain the voluntary nature of the study and that the focus group will be recorded with permission. If not already done, set up and switch on the recording equipment while participants sign the consent form.
- Before starting the focus group, remind participants about confidentiality and ask participants not to talk over each other. Go around the circle and ask participants to introduce themselves for the transcription.

Rationale: to explore whether the findings resonate with patients and members of the public in their local community

Discussion of Phase 1 interview findings

Provide a short overview of key findings from Phase 1 interviews regarding barriers and enablers to lung cancer awareness and early symptom presentation in the target group. Ask the group to share their thoughts on the interview findings, and whether they resonate with them.

'We did some interviews across the UK with people who smoke or used to smoke and have a lung condition like COPD. We found that people look out for symptoms of a chest infection most days. They were really good at knowing when they had a chest infection and going to the doctor to get antibiotics. We also found that people try not to think about health problems that might affect them in the future, so may not go to the doctor with some symptoms that could be serious.

People really didn't like it when the doctor told them to stop smoking and some people felt like they were treated differently by the doctors because they smoked. Sometimes this put people off going to the doctor with lung symptoms.

We also found that people often had a favourite doctor at their GP practice and would wait up to three weeks for an appointment even if they thought a symptom was important. People don't like

For peer review only

going to doctors that they don't know because they felt that they did listen as much as their favourite doctor. But sometimes it was necessary to go to a doctor they don't know to get an appointment the same day.

We want to develop something that can help people get important lung symptoms seen to quicker by a medical professional, but we are not really sure how to do this or who should do this. We would like to know what you think.'

- What do you think of these findings?
- In what ways do you feel the same as what we found?
- In what ways do you feel different to what we found?

Rationale: to seek views on how to access the target group for an intervention and explore preferences for an intervention to support earlier presentation, including mode of delivery, target group, content and stop smoking information

For peer review only

Part 2. Needs and preferences for an intervention

Preferences on intervention format

- In what ways could we support people to manage their lung health?
    - Probe: a group one-off educational session to promote "lung health" in the local community; a leaflet/DVD; a lung health check; event in the community; posters in the local community
- Who do you think would be best to lead an intervention about lung health?
    - Probe: lung cancer survivor, lay advisor, community partner, healthcare professional
- If a health care professional was to lead the intervention, who would be best to lead a lung health intervention?
- Who is your favourite healthcare professional?
- When would be best to support people to manage their lung health?
    - What about when someone is diagnosed with a lung condition?
    - What about in one of your regular check-up appointments with the nurse i.e. six-monthly COPD clinic review?

Preferences on intervention content

- **What would you like to know? OR what skills would you like to learn?**
    - What would be most useful or important for you?
    - Can you think of anything that we should avoid or anything that is not as important?
- What do you think about including information to help people to stop smoking?
    - How would you react to stop-smoking information?
    - Can you think of ways we might be able to include stop-smoking information without putting smokers off?
    - What do you think about telling people where they can get help to stop smoking if they want to, instead of giving people information about stop smoking?

Accessing intervention participants

- Where could we approach people who smoke/used to smoke and have lung symptoms?
    - Probe: through community pharmacies, primary care, community nurses, existing groups for people with lung conditions/ utilising family and social networks / snowballing approaches?
- Are you aware of any community groups for these types of people?
    - How do you think people from these groups would react to being approached for a lung health intervention?

Debrief

'Thank you for taking part in this study. We hope to use the findings from this focus group to develop something to encourage people to go to the doctor with important lung symptoms. Anything you said will be treated as confidential. The voice-recoding will be stored securely. Any quotes used in published research will not have your name or anything that could identify you. Do you have any questions? [*answer any questions*] Here are my contact details if you have any further questions.'

For peer review only

BMJ Open

Engaging high-risk groups in early lung cancer diagnosis: a qualitative study of symptom presentation and intervention preferences amongst the UK's most deprived communities

Journal:	*BMJ Open*
Manuscript ID	bmjopen-2018-025902.R2
Article Type:	Research
Date Submitted by the Author:	18-Jan-2019
Complete List of Authors:	McCutchan, GM; Cardiff University, Division of Population Medicine<br>Hiscock, Julia; Bangor University, North Wales Centre for Primary Care Research<br>Hood, Kerenza<br>Murchie, Peter; University of Aberdeen, Division of Applied Health Science<br>Neal, Richard; University of Leeds<br>Newton, Gareth; Cardiff University, Division of Population Medicine, Patient and Public Involvement<br>Thomas, Sara; Public Health Wales, Cwm Taf Public Health Team<br>Thomas, Ann Maria; Cardiff University, Division of Population Medicine, Patient and Public Involvement<br>Brain, Kate; Cardiff University, Division of Population Medicine, School of Medicine
**Primary Subject Heading**:	Oncology
Secondary Subject Heading:	Respiratory medicine, Qualitative research, Public health, Health policy, Smoking and tobacco
Keywords:	PRIMARY CARE, PUBLIC HEALTH, QUALITATIVE RESEARCH, RESPIRATORY MEDICINE (see Thoracic Medicine), lung cancer, socioeconomic status

SCHOLARONE™
Manuscripts

Title page

Title: Engaging high-risk groups in early lung cancer diagnosis: a qualitative study of symptom presentation and intervention preferences amongst the UK's most deprived communities

Authors: Dr Grace M McCutchan[1$], Dr Julia Hiscock[2], Prof Kerenza Hood[3], Prof Peter Murchie[4], Prof Richard D Neal[5], Mr Gareth Newton[1], Mrs Sara Thomas[6], Mrs Ann-Maria Thomas[1], Prof Kate Brain[1]

Affiliations:

[1]Division of Population Medicine, School of Medicine, Cardiff University, Cardiff, UK

[2]North Wales Centre for Primary Care Research, Bangor University, Wrexham, UK

[3]Centre for Trials Research, School of Medicine, Cardiff University, Cardiff, UK

[4]Division of Applied Health Sciences, Section of Academic Primary Care, University of Aberdeen, Aberdeen, UK

[5]Leeds Institute of Health Sciences, Faculty of Medicine and Health, University of Leeds, Leeds, UK

[6]Cwm Taf Public Health Team, Public Health Wales, Keir Hardy University Health Park, Merthyr Tydfil, UK.

[$]**Corresponding author:** Grace McCutchan. Postal address: Division of Population Medicine, Cardiff University, 1st Floor, Neuadd Meirionnydd, Heath Park, Cardiff, CF14 4YS. Email: mccutchanGM@cardiff.ac.uk. Telephone: +44 (0)2920 687639.

Word count: 5994

For peer review only

Abstract

Objectives

People at high-risk for lung cancer - current/former smokers, aged 40+ years, with serious lung comorbidity (i.e. Chronic Obstructive Pulmonary Disease) and living in highly deprived areas - are more likely to delay symptom presentation. This qualitative study aimed to understand the influences on early presentation with lung cancer symptoms in high-risk individuals and intervention preferences.

Methods

Semi-structured qualitative interviews with 37 high-risk individuals (without a cancer diagnosis), identified through seven GP practices in socioeconomically deprived areas of England, Scotland and Wales (most deprived 20%). A symptom attribution task was used to explore lung symptom perception and help-seeking, developed using Leventhal's Common Sense Model. Four focus groups with 18 high-risk individuals and 16 local stakeholders (healthcare professionals and community partners) were conducted to explore preferences for an intervention to promote early lung cancer symptom presentation. Data were synthesised using Framework analysis.

Results

Individual and area level indicators of deprivation confirmed that interview participants were highly deprived.

Interviews. Preoccupation with managing 'treatable' short-term conditions (chest infections), led to avoidance of acting on 'inevitable and incurable' longer-term conditions (lung cancer). Feeling judged and unworthy of medical help because of their perceived social standing or lifestyle deterred medical help seeking, particularly when difficult life circumstances and traumatic events led to tobacco and alcohol addiction.

Focus groups. Participants recommended multi-faceted interventions in community venues, with information about lung cancer symptoms and the benefits of early diagnosis, led by a trained and non-judgemental facilitator.

For peer review only - http://bmjopen.bmj.com/site/about/guidelines.xhtml

Conclusions

This study was novel in engaging a high-risk population to gain an in-depth understanding of the broader contextual influences on lung cancer symptom presentation. Perceived lack of health service entitlement and complex lives facilitated avoidance of recognising and presenting with lung cancer symptoms. Community-based interventions have the potential to empower disadvantaged populations to seek medical help for lung symptoms.

Key words: Lung cancer, deprived, qualitative, early diagnosis, medical help seeking, barriers.

Strengths and limitations of the study

- This was the first study to use in-depth qualitative methods to explore how to engage high risk individuals from socioeconomically deprived areas in early lung cancer diagnosis.
- A major strength of this study was the proactive and rigorous sampling procedures used to ensure that our sample was at high risk for lung cancer.
- Assessment of individual and area level indicators of deprivation confirmed that interview participants were highly deprived; all participants resided in the 20% most deprived areas of the three UK nations, and most participants were unemployed/seeking benefits and/or rented social housing.
- To overcome the methodological limitations associated with studying anticipated or retrospectively recalled cancer symptom presentation, we recruited participants with no previous diagnosis of lung cancer and did not mention lung cancer in the interview study materials or during completion of the symptom attribution task.
- Opportunistic recruitment of focus group participants who may have been more favourably disposed to an intervention was a potential study limitation.

For peer review only

INTRODUCTION

Lung cancer is the leading cause of cancer mortality worldwide [1]. Outcomes are among the poorest for all cancers, with only 13% of lung cancer patients surviving five or more years in the UK [2]. Diagnosis of lung cancer at an early stage can enable curative surgical resection, meaning over 80% of patients will survive one year or more when diagnosed at Stage I[3]. Delayed medical help seeking for symptoms and the high proportion of lung cancer diagnoses through emergency departments may partly explain why lung cancer is commonly diagnosed at an advanced, incurable stage[4]. Due to low specificity of lung cancer symptoms and similarity to other acute and comorbid respiratory conditions, patients face difficulty in knowing when to seek medical help[5,6].

Multiple symptoms and risk factors for lung cancer including older age, smoking, the presence of a lung comorbidity and socioeconomic deprivation increase the likelihood that a patient presenting to their GP with symptoms indicative of lung cancer will receive a cancer diagnosis[7-9]. Lung cancer is more common and mortality higher in areas of high socioeconomic deprivation; it has been estimated that each year, socioeconomic inequalities account for 11,700 excess cases of lung cancer and 9,900 potentially avoidable lung cancer deaths in England[10]. High prevalence of smoking, lung comorbid conditions and asbestos exposure, all of which are well documented risk factors for lung cancer, contribute to high lung cancer incidence and mortality in deprived communities[11, 12].

The presence of lung comorbidity such as chronic obstructive pulmonary disease (COPD) and history of smoking have been associated with a lower likelihood of presenting with lung cancer symptoms early[13,14]. In the lead up to lung cancer diagnosis, vague symptoms may go unnoticed or not considered a legitimate symptom to seek medical attention for, or be misattributed to smoking, aging or other comorbid conditions such as heart disease or COPD, thereby prolonging help seeking[5,13,15-26]. In addition, stigma attached to lung cancer[23,27-30] and fear of lung cancer diagnosis can deter medical help seeking for symptoms, particularly among smokers[23-25,29,31-33], leading to advanced stage disease at diagnosis[19]. To date, research has mainly been conducted with lung cancer patients from a range of socioeconomic groups with varying levels of lung cancer risk, retrospectively exploring the barriers to symptom presentation. Evidence is lacking about how individuals who are at high risk, and without a diagnosis of lung cancer, attribute potential lung cancer symptoms and decide to seek medical help.

Strategies to prompt earlier help seeking for lung cancer symptoms are required. However, evidence is limited regarding optimal methods for promoting earlier presentation through interventions

For peer review only - http://bmjopen.bmj.com/site/about/guidelines.xhtml

targeted at high risk, highly deprived groups. Mass media[34] and community based social marketing[35] lung cancer campaigns report limited reach to the most deprived groups. A nurse-led primary care intervention for older adults with a long smoking history or recent cessation reported increased and sustained intentions to seek help with lung cancer symptoms[36]. However, the intervention was not targeted at highly deprived groups. Novel methods to support high risk groups to engage in early lung cancer diagnosis are required.

The current study used a combination of interviews and focus groups to explore potential barriers to early lung cancer diagnosis and strategies to encourage early help seeking with individuals who are the high risk for lung cancer. Qualitative interviews were used to gain an in-depth understanding of the processes and motivations involved in symptom attribution and medical help seeking for potential lung cancer symptoms in high risk, highly deprived individuals. We targeted socioeconomically deprived areas across three nations of the UK to approach potential participants, and used rigorous sampling procedures to ensure that our sample were high risk for lung cancer. The focus groups were conducted in highly deprived areas with stakeholders who lived or worked in these communities. To our knowledge, this was the first study to explore the influences on early lung cancer diagnosis and intervention preferences targeted at high risk groups living in the most deprived areas of the UK.

METHODS

The Consolidated Criteria for Reporting Qualitative Research (COREQ)[37] criteria were used to guide reporting (Supplementary File 1). We used a combination of interviews and focus groups because the interviews were framed around lung health (not lung cancer), whereas the focus groups were framed around preferences for a lung cancer intervention. In addition, key interview findings were presented in the focus groups for consolidation and to facilitate discussion about intervention preferences.

Participant recruitment and sampling

Interviews. Thirty-seven interview participants were recruited through seven primary care general medical practices (GP) in South Wales (Cwm Taf: three practices), England (Liverpool: one practice) and Scotland (Aberdeen: three practices). Using routinely published index of multiple deprivation (IMD) data for England, Scotland and Wales, GP practices with the highest proportion of their patients that reside in the most deprived quintile were contacted. Practice managers were asked to screen GP practice databases purposively for eligible study participants: men and women over the

For peer review only - http://bmjopen.bmj.com/site/about/guidelines.xhtml

age of 40, who were current or former smokers, with a lung condition (COPD including chronic bronchitis and emphysema, interstitial lung disease or occupational lung disease). To overcome methodological limitations associated with retrospective recall, we recruited participants with no previous diagnosis of lung cancer. Participants were initially recruited from GP practices in Cwm Taf, where practice managers were asked to screen databases for current and former smokers, with no parameter set for number of years since quit attempt. Due to an initially high response rate from former smokers in Cwm Taf, subsequent participants in Aberdeen and Liverpool were sampled purposively according to smoking history. One GP practice in Aberdeen was asked to recruit current smokers and recent quitters (within ten years). Two GP practices in Aberdeen and one GP practice in Liverpool recruited current smokers only.

To ensure that participants from highly deprived areas were invited to take part in the study, individual postcodes were screened by the research team. Eligible patients from the initial database screen were assigned a pseudo-anonymised participant identifier (PID). PID and postcode were checked against IMD score, and those that resided in the most deprived IMD quintile were eligible for the study. The final list of potentially eligible participants was checked by the GP for ability to provide informed consent, considered by the GP to be a risk to the interviewer or themselves and general health status (i.e. very seriously ill). Participants were excluded if they were diagnosed with lung cancer, were terminally ill or did not have capacity to consent.

Focus groups. Eighteen participants for the focus groups with members of the public were recruited opportunistically through primary care or local community groups. Participant recruitment through primary care employed the same methods as those used to invite the interview participants. Pseudonymised participant identifiers were checked to ensure that those who took part in the focus groups had not already participated in the interviews. Additional participants were recruited opportunistically through local community respiratory support groups and non-health related groups in the local community centre. Local community group organisers in areas of high deprivation were contacted and asked for help to recruit members of the public in our target group. Local health service planning groups and health board staff facilitated recruitment of 12 participants for the healthcare professional and community partner (HPCP) focus groups.

For peer review only - http://bmjopen.bmj.com/site/about/guidelines.xhtml

Study procedures

Written consent and permission to audio-record were obtained on the day of the interviews and focus groups.

Interviews. Eligible participants were invited by letter with more detailed study information attached, with a reminder at two weeks to non-respondents. Those who returned the study reply slip via a FREEPOST envelope were contacted by the interviewer (GM or JH) to arrange a suitable time and date for the interview, to outline the study and answer any questions.

Interviews were conducted using a semi-structured topic guide to facilitate a discussion about illness perceptions and coping strategies; development was guided by the Common Sense Model[38] (Supplementary File 2). The interview was framed the interview around lung health, rather than lung cancer. The interview aimed to explore experiences of their lung condition, symptom attribution, symptom experience and help seeking behaviour, the influence of smoking history on new or changing symptoms, and if appropriate, lung cancer awareness and beliefs.

A symptom sorting task was used to provide participants with a concrete visual task to increase engagement with the interview in the context of potential low literacy. The task formed a basis for discussion about symptom attribution and experience, where participants were asked to order 11 symptoms from those they would go to the doctor with first, through to the last. The 11 symptoms were selected from the NICE guidance for referral of suspected lung cancer (https://www.nice.org.uk/guidance/cg121). The symptoms were re-worded to simplify the language in line with wording found on the NHS Choices website for lung cancer symptoms and any reference to time scale of symptoms was removed (Supplementary File 2, p.9). For example, a cough that lasts for 3 weeks or more was amended to ‘persistent cough’, and haemoptysis was amended to ‘coughing up blood’. The presentation order of the symptoms was rotated between interviews.

To explore potential lung cancer symptom attribution outside of a cancer context, there was no mention of cancer in the interview study information packs or when participants completed the symptom sorting task. If appropriate, participants were asked questions to explore lung cancer awareness and beliefs at the end of the interview or when participants discussed lung cancer unprompted.

For peer review only - http://bmjopen.bmj.com/site/about/guidelines.xhtml

Demographic data were collected using a short questionnaire, including three additional measures of socioeconomic group: age, gender, smoking status (quantity and duration), home ownership, occupation and educational attainment. Interviews were conducted until data saturation (no new themes emerging[39]).

Focus groups*.* High risk members of the public*,* and healthcare professionals (i.e. GP, nurse, community pharmacist/community partners working in areas of deprivation with people with smoking history and/or lung conditions were sent information about the study and invited to take part in focus groups. Focus group participants were explicitly informed that the study was about the development of an intervention about lung cancer. A mutually convenient time, date and location for the focus groups was agreed. The focus groups were conducted using a semi-structured topic guide to explore preferences for an intervention to promote earlier lung cancer diagnosis. Separate topic guides were used for the public and professional groups (Supplementary file 3 and 4). Participants were given a verbal summary of the key findings from the qualitative interviews, and asked to discuss preferences for a potential lung cancer intervention targeted at high risk, highly deprived individuals. Topics for discussion were: preferred format of an intervention, recommendations for intervention content, preferred location and facilitator for intervention delivery, and recommendations for the inclusion of smoking cessation advice.

Setting

Most interviews (n=34) took place face-to-face in participant's own homes, with three taking place in a café, local community centre or over the telephone, and lasted between 46 and 146 minutes (mean 83 minutes). Family members were present for three interviews but did not participate in the study. Focus groups took place in primary care settings (n=2) or local community centres (n=2). Members of the public who took part in the interviews or focus groups were compensated with a £10 shopping. Healthcare professionals and community partners were not reimbursed for their time.

Interviews and focus groups in England were conducted by JH (PhD), a trained and experienced female qualitative Research Fellow and Medical Sociologist. The Welsh and Scottish interviews and focus groups were conducted by GM (PhD), a female Health Psychologist and trained qualitative Research Associate.

For peer review only - http://bmjopen.bmj.com/site/about/guidelines.xhtml

Data analysis

Interviews and focus groups were audio-recorded and transcribed verbatim. Anonymised transcripts were analysed in detail using the Framework method[40]. Framework analysis is a well-respected and commonly used approach to qualitative data analysis. It was considered particularly suitable for this study due to its transparency and the team work involved[41]. Framework enabled the sharing of synthesised data charts among team members to facilitate participation in analysis and interpretation workshops.

The data were analysed in five stages: familiarisation, identification of a thematic framework, indexing, charting, and interpretation. A separate index was created on Microsoft Excel for the interview and focus group data; however, wherever possible, overlap was coded using the same indexing terms, for example 'barriers to symptom presentation' was commonly discussed in both the interview and focus groups. The index was developed by two researchers (GM and JH). Themes were generated independently and consolidated through discussion in nine interpretation workshops over a nine month period by GM and JH. The different perspectives of the researchers as noted above was a benefit during analysis and interpretation. Field notes were recorded for each interview and focus group, and incorporated into discussion during the analysis workshops. Although not formally incorporated into the analysis plan, the positioning of each symptom in the attribution task was considered during interpretive workshops. Interpretive themes were generated by JH and GM, and developed with all authors in monthly management meetings. Transcripts and study findings were not checked by participants; however, all participants were mailed a summary of the study findings.

Ethical approval

The study received ethical approval from Southampton Central- Hampshire A Research Ethics Committee (16/SC/0589).

Patient and public involvement. Patient and public representatives (AMT and GN) were involved in the design of the study and interpretation of study findings in monthly management group meetings. All study materials and topic guides were developed with lay input (AMT and GN) and written to a reading age of 10 years due to potentially low literacy. Reading age was calculated using the Automated Readability Index (www.readabilityformulas.com).

For peer review only

RESULTS

Interviews

Of the 397 invited to take part in the study, 78 people returned the study reply slip and declined to participate in the study; reasons for refusal were unknown. Thirty-seven participants agreed to take part in the study. The majority of the sample were female, current smokers, and with a mean age of 65 years (Table 1). Most had a diagnosis of COPD. All 37 participants resided in the lowest quintile of deprivation for their respective country, of whom 15 were in the most deprived decile. Most participants had left school before age 15 with no formal qualifications, lived in social housing, and claimed disability benefit or job seekers allowance.

Table 1. Qualitative interview sample characteristics

**Sample characteristics**	**Total n=37**
**Gender**	
Male	16
Female	21
**Age, years**	
Mean (range)	64.7 (48-84)
**Smoking status**	
Current smoker	18
Occasional smoker	3
Former smoker, recent quitter (within five years)	5
Former smoker (quit over five years ago)	11
**Deprivation decile**	
Welsh Index of Multiple Deprivation (WIMD)	
Decile 1 (most deprived 10%)	5
Decile 2 (most deprived 11-20%)	10
Scottish Index of Multiple Deprivation (SIMD)	
Decile 1 (most deprived 10%)	4
Decile 2 (most deprived 11-20%)	12
English Index of Multiple Deprivation (IMD)	
Decile 1 (most deprived 10%)	6
**Self-reported lung condition**	
COPD	26
Chronic bronchitis	2
Chronic emphysema	2
Occupational lung disease	1
Unsure of diagnosis	4
Missing	2
**Educational attainment**	
Left school at/before age 15	29
Completed CSEs, O-Levels or equivalent	5
Completed A levels or equivalent	1
Completed further education but not degree	1

For peer review only - http://bmjopen.bmj.com/site/about/guidelines.xhtml

Missing	1
**Employment**	
Employed full-time	2
Employed part-time	1
Casual work	1
Job seekers or disability benefit	17
Retired	16
**Home/living arrangement**	
Own flat/house	14
Rent from local authority/housing association	21
Rent privately	1
Missing	1

Key themes were: strategies involved in symptom detection and help seeking behaviour, maintaining short term health, avoidance of acting on longer term health, the desire to be a model patient, and the importance of the relationship with their healthcare professional. See Table 2 for illustrative quotes.

Symptom detection strategies and help seeking

Symptoms discussed during the task were viewed as "part and parcel" (male, 68, England, current smoker) of their lung condition, other pre-existing comorbidities or smoking habit, and were consequently normalised and perceived not to require medical help. Changes to vague or respiratory-type lung cancer symptoms were only taken seriously when remarked on by friends and family or when they impacted on daily life.

Symptoms that could indicate a chest infection were reportedly constantly monitored. Participants discussed using sophisticated strategies such as noticing changes in the colour and consistency of their phlegm or subtle audible changes in their cough to actively detect chest infections. Such strategies were considered important to facilitate early detection and treatment for chest infections through their primary care provider or with rescue packs (emergency packs of steroids and antibiotics that can be kept at home), due to lung condition.

Constant monitoring of phlegm for control of lung condition meant that participants could and would notice haemoptysis, but few reported actively looking for haemoptysis on a regular basis. Disparity between actual and anticipated medical help seeking was reported for haemoptysis. Most participants had not previously experienced haemoptysis, but would anticipate seeking medical help immediately due to the potentially serious nature of blood. However, some participants who had

For peer review only

previously or were currently experiencing haemoptysis attributed the presence of blood to non-cancer causes such as their stomach ulcer or a previous flu jab. One participant ascribed the blood in their cough to lung cancer. Some of the participants with experience of haemoptysis did not seek medical help.

Focus on maintaining short term health

Participants reported seeking medical help quickly when symptoms were easy to detect, were attributed to what was perceived as a treatable cause and represented an immediate health threat i.e. a chest infection due to lung condition. Participants could often request an appointment the same day as permitted by their GP surgery policies. Prompt help seeking was reportedly due to fear of not being able to breathe and the potentially life-threatening nature of chest infections, and is likely to reflect the need to maintain good health in the short term.

The focus on maintaining short term health may reflect low general expectations of health, where some participants disclosed surprise at living beyond 60 years of age. In addition, due to fear of potentially hearing bad news, some participants expressed a preference to not ask questions during a consultation or yearly review with the nurse. Participants discussed prioritising day-by-day living over longer term planning, thereby focusing on health in the short term.

Avoidance of acting on longer term health

Most participants discussed scepticism about the link between lung cancer and smoking. Conversely, participants thought that lung cancer was inevitable due to their current or former lifestyle, including smoking history, working conditions, their lung condition and the reported incidence of lung cancer in their community. For many participants, the topic of lung cancer arose spontaneously. Lung cancer was discussed in the context of perceived inevitability when reflecting on their general lung health and during completion of the symptom task when recalling friends/family with lung cancer. Beliefs about inevitability were often coupled with highly negative fearful and fatalistic beliefs about lung cancer, with no cure and eventual death. Such claims were evidenced by knowing a high proportion of friends and family who were diagnosed with lung cancer and often died. A few participants discussed that a cure for lung cancer involved luck or was ‘some miracle’ (male, 56, Wales, occasional smoker), reflecting a perceived lack of control over early diagnosis and treatment. Consequently, actual or anticipated medical help seeking for lung cancer symptoms was motivated

For peer review only - http://bmjopen.bmj.com/site/about/guidelines.xhtml

by pain, or to seek a diagnosis and prognosis to notify family members. However, some participants anticipated refusal of treatment or would even contemplate suicide.

We found differences in how participants with and without dependent family reported responding to symptoms of lung cancer. Female participants with dependent children or grandchildren discussed a motivation to visit the doctor with symptoms suggestive of lung cancer, in order to receive a prognosis to enable childcare arrangements after death. Women with dependent children who held more positive beliefs about lung cancer treatment reported the need to seek help for treatment to 'stay healthy' and prolong life. Participants with no dependent family were more likely to ignore lung cancer symptoms, or anticipate seeking medical help if in pain but refuse treatment.

The model patient

Participants discussed a sense of lack of entitlement to health services due to smoking habit, where respiratory-type symptoms of lung cancer were perceived as self-inflicted. For some, this was reinforced by an actual or expected 'smoking lecture' each time they sought help from healthcare professionals; the lecture made participants feel ostracised, particularly when smoking was used as a coping mechanism and contributed to not feeling worthy of seeking medical help. Some participants perceived that they may be treated differently by health professionals because they live in an area of deprivation, and discussed a potential power imbalance during consultations.

Conversely, participants reported high criticism towards people who were perceived to waste, exploit and overuse NHS resources. They cited drug addicts, illegitimate benefits claimers, older people wanting social interaction, and people with coughs and colds as over users of the health service. Such beliefs may reflect a downward comparison to other more stigmatised service users to legitimise their own help seeking. In order to be considered a model and non-problem patient, participants discussed legitimising their own help seeking by only consulting when absolutely necessary - and often after trying their 'own cures' i.e. cough medicine from the pharmacist - to not burden the doctors. Infrequent attenders or 'good service users' discussed feeling a sense of superiority for being a model patient.

For peer review only - http://bmjopen.bmj.com/site/about/guidelines.xhtml

Relationship with the healthcare professional

Some participants disclosed traumatic events in their lives including physical and sexual abuse, leading to tobacco dependence and alcohol addiction. In addition, more than half of the sample described symptoms of depression and anxiety. Therefore, the reported relationship with their healthcare professional was important when considering whether to present with lung symptoms. Participants discussed the need to feel understood and not judged by their healthcare professional, with their personal history taken into account in the context of health behaviour such as smoking.

Those who discussed feeling comfortable, safe and not judged by their chosen healthcare professional felt encouraged to present with symptoms. Some participants reported that they were prepared to wait up to three weeks for an appointment with their preferred healthcare professional to discuss worrisome and potentially serious symptoms that could indicate lung cancer. Many participants reported problems with maintaining continuity of care, highlighting problems with the stretched National Health Service.

Table 2. Illustrative quotes (qualitative interviews)

Theme	Quote
**Symptom detection strategies and help seeking**	
Friends and family notice symptoms	*"My daughter might [notice changes to symptoms] cos she mentions it now and then…she'll give me a dig and she'll say 'your breathing's annoying me'. Cos it's heavy breathing so. Then again there's something wrong"* (Male, 48, Scotland, former smoker)
Sophisticated symptom detection strategies/ monitoring of chest infections	*"If [phlegm is] white and bubbly it's not a chest infection. It's only when it goes green so you can tell yourself exactly how close you are to getting an infection… There's just two different kinds of green spittle, if it's fluorescent green then you've got an infection, normal antibiotics won't work with me, if it's the lighter green I'm fine with that one… it's handy to look out for, because you can get the right medication at the right time…because if anything happens to me, there's no one for my kids."* (Female, 48, Scotland, current smoker)
Normalisation of haemoptysis	*"Coughing up blood, I do actually get some of that I don't know why, but it could be because of the ulcer thing and that…There again then well I do get like nosebleeds, and then I'm thinking the blood maybe coming inside and coming down, you swallow it see. So then that will come back up won't it"* (Male, 62, Wales, former smoker)

For peer review only - http://bmjopen.bmj.com/site/about/guidelines.xhtml

**Focus on maintaining health in the short term**	*"I get worried about having chest infection, I get more worried about today or tomorrow rather than the future. The future that's going ahead for us anyway. Lung cancer's not an issue really"* (male, 50, Scotland, former smoker)
Fear of bad news during a consultation	*"I'm very poor in asking questions cos I don't want to know the results. Simple as that…no I don't ask when they say the oxygen [saturation] is alright I just think well it's alright and it's one thing less I haven't got to worry about"* (Female, 69, Wales, former smoker)
**Avoidance of long term health outcomes**	
Scepticism about the link between smoking and lung cancer	*"You hear occasions where people who don't smoke, who've never smoked. Well how do they get their lung cancer?…I've got [lung cancer] in my head, I'm probably going to get it, if I haven't already got it because of the lifestyle I've had. Where I've worked and everything else, what I've worked with."* (Male, 68, England, current smoker)
Perceived inevitability of lung cancer/ anticipate suicide	*"[Lung cancer] is really, really on the forefront on the mind…I just think 'oh god, please don't let me get cancer'…I think if I was to get cancer, I've sometimes said to myself, I'd commit suicide. I would take a pill or something."* (Female, 81, Scotland, current smoker)
Avoidance of lung cancer due to social and contextual factors	*"[Lung cancer] worries me but I've got proper problems to worry about [carer for disabled son, problems with social services and benefits claims, insecurity of current council owned housing and problems with area of residence with 'junkies']. I won't worry about it until it's actually here. If I started worrying about eventualities I'd never get anywhere"* (Female, 48, Scotland, current smoker)
Lung cancer fatalism/ anticipated refusal of treatment	*"Until anything happened and I'm actually told that I've got [lung cancer], there's nothing I can do about it. I'm really a believer of what's in your cards is already written. So I don't look at anything like that…But if they told me it was cancer, I would go ok then, but I wouldn't take any of the treatments… if it's my time, it's my time. It just doesn't, I don't think I've got any more fight in me for all that. I think that would be the last straw for me. So I just live every day as it comes now, I don't really plan much. So I'm just living in the day, you know. Cos whatever happens, happens anyway."* (Female, 49, Scotland, current smoker)
Response to lung cancer symptom/ female with dependent family	*"I don't think they can treat [lung cancer]. You've just got to accept it haven't you…I would go to the doctor [with a symptom], I think I would like to know how long I had. Not for me but for [my son] you know. For him…If it was just me I wouldn't want to know, but because I've got him, [I would] definitely…When I seen the blood I did think to myself, I flushed it away right away…I seen the blood and I thought no, and I thought I've got to, you know,*

For peer review only - http://bmjopen.bmj.com/site/about/guidelines.xhtml

	because of [my son]. The only way I would want to know is because of him. If I was by myself I would just say, don't want to know…Can't just think about myself I've got to think about him as well." (Female, 68, Scotland, current smoker)
**The model patient**<br>Perception of healthcare professionals attitude to smokers<br><br>Critical of people who waste NHS resources	*"You feel as though you're an alien because you smoke, you feel as so they just look at you and say 'urghh', you know"* (Female, 52, Scotland, current smoker)<br><br>*"I can guarantee if I went this Monday and go next Monday the same people are sitting there. I'm being honest, they're a drain on society on the NHS, but that's the way they live…these people that go there are not really ill, I think they're just seeking attention"* (Male, 78, England, current smoker).
**Relationship with healthcare professional**<br>Disclosure of highly sensitive personal problem<br><br>Good relationship with GP	*"Some people are friendly and not stony faced…if [the HCP] can't even start a conversation with the simplest of ice breakers then how can people tell about pooping themselves when they're coughing up"* (Female, 48, Scotland, current smoker)<br><br>*"I'm alright with [one GP], you could tell her anything, I've shocked her sometimes"* (Female, 51, England, current smoker)

Focus groups

Two public focus groups were conducted in Wales and England. Most participants were female and former smokers, and all participants were diagnosed with a lung condition. Two professional focus groups were conducted in Wales. Most participants were female, and were medical professionals (Table 3).

Table 3. Focus group characteristics

**Members of the public**	**N participants**	**Healthcare professionals and community partners**	**N participants**
**Group 1, England**	**total n=7**	**Group 3, Wales**	**total n=5**
Gender		*Gender*	
Female	6	Female	2
Male	1	Male	3
Smoking status		*Occupation*	
Current smoker	3	Community nurse	1
Former smoker	3	Support group facilitator	1
Never smoker	1	Community partner	1
Self-reported lung condition		Third sector representative	1
COPD	7	Public health representative	1
Recruited through Primary Care and community groups		Recruited through the Health Board	

For peer review only - http://bmjopen.bmj.com/site/about/guidelines.xhtml

Group 2, Wales	total n=9	Group 4, Wales	total n=7
Gender		*Gender*	
Female	5	Female	6
Male	4	Male	1
Smoking status		*Occupation*	
Current smoker	3	Practice manager	1
Former smoker	4	Pharmacist	1
Never smoker	2	GP	2
Self-reported lung condition		Practice nurse	2
COPD	9	Medical student	1
Recruited through community groups		Recruited through the Health Board/ Primary Care	

Key themes discussed were: barriers to early lung cancer diagnosis, and preferences regarding the format and content of an intervention for the early detection of lung cancer. See Table 4 for illustrative quotes.

Barriers to lung cancer symptom presentation

The public and stakeholder focus groups confirmed our interview findings, where fear of wasting the doctor's time with trivial symptoms and fear of being judged or lectured about smoking was perceived to deter medical help seeking for potential lung cancer symptoms. In addition, the health professional group supported our findings that patients with lung conditions tend to be preoccupied by chest infections. However, we found potential disparity between the patient-reported experience of the GP's approach to smoking and the healthcare professional reported approach to smoking cessation. Healthcare professionals in Wales discussed new guidance that discourages health professionals from 'lecturing' patients, suggesting the patient reported experience may be based on previous healthcare interactions, and they consequently anticipate a lecture. Alternatively, healthcare professionals may be unaware of new guidance, or not adhere to new guidance and consequently continue to 'lecture' patients about smoking.

Potential format of an intervention to support earlier lung cancer diagnosis

All groups discussed a preference for community based interventions, away from a traditional healthcare setting, for example a community event, talk in a community venue or health check bus, similar to breast screening mobile units. The anonymous and relaxed nature of such an intervention meant that intervention participants would feel they were not wasting GP time; rather it would act as a signal that their attendance at the event was desired. Participants compared this to a visit to the

For peer review only - http://bmjopen.bmj.com/site/about/guidelines.xhtml

doctor, where they discussed a feeling of wasting the GP's time because they were not invited to attend. It was considered important that the intervention facilitator was knowledgeable or trained, non-judgemental, easy to talk to and approachable, highlighting the importance of relational aspects of a lung cancer intervention. Participants suggested a nurse, pharmacist, trained patient representative or community worker.

Intervention content

The public groups requested more information about the symptoms of lung cancer. However, the healthcare professional groups felt that current lung cancer symptom information was too broad, leading to dismissal and potential avoidance of lung cancer information because people with smoking history or comorbid lung conditions experience most of the symptoms daily. To overcome this problem, the healthcare professionals groups discussed the need for more specific symptom information, emphasising changes to normal symptoms and coupled with information about risk factors for lung cancer.

To modify negative beliefs about lung cancer, the health professionals groups suggested using positive stories to communicate messages about the importance of lung cancer early diagnosis and highlight the potential for survival outcomes with early stage detection.

The inclusion of smoking cessation information in a lung cancer intervention was considered important by all groups. However, the manner in which smoking cessation could be approached was discussed as key to effective promotion of smoking cessation. Participants suggested highlighting the benefits of stopping smoking in a gentle and relaxed manner to encourage choice to quit.

Table 4. Illustrative quotes (focus groups)

Theme	Quote
**Barriers to lung cancer symptom presentation**	
Fixation on chest infections	*"People tend to be fixated on a [chest] infection and they want their next rescue pack ready cos almost as if it's inevitable; it's going to happen in the next month or so."* (Focus group 4)
Difference in perception around healthcare professional approach to patients' smoking	*"I think there is a gulf between what people believe their GP would say to them if they do actually talk about [smoking] as opposed to what that conversation actually is in reality….But certainly as far as the formal training coming out of public health, if they are doing that then there is, that's not a lecture…But that's what people fear is going to be what they're going to be told"* (Focus group 3)

For peer review only - http://bmjopen.bmj.com/site/about/guidelines.xhtml

**Potential format of an intervention to support earlier lung cancer diagnosis**	*Participant 2: "So what I'm saying is, you know them mobile buses… in the shopping area, where people go shops, or outside the hospital… So they set them up and people are walking past, and even though they can't be bothered to go to the doctors, and they look and they think I'll just pop in*<br>*Participant 1: Cos you wouldn't hesitate you know, you'd just go in.*<br>*Participant 2: You're just a person, they don't know and they're just seeing what's there, or what's there or what's the problem with you. If there's no problem*<br>*Participant 3: People think you don't want to think you're, feel as if you're wasting the doctor's time"* (Focus group 1)
**Intervention content**<br>More specific symptom advice	*Participant 1: "Yeah I think when you say 'cough' it's a bit broad and it's a bit…You know, you've had a cough for two weeks, off you go.*<br>*Participant 3: It'd be useful if it was a change in your regular cough"* (Focus group 4)
Messages to combat negative beliefs	*"Positive messages, particularly around lung cancer because everybody, you know it's like a death knell isn't it? And actually it's not, it doesn't have to be. You know you're talking here about early diagnosis which is a big deal isn't it"* (Focus group 3)
Smoking cessation	*"You've got to include [smoking cessation information]…I think it's how you deliver the message…not in such a way you feel ashamed for smoking. I've noticed [the nurse] has got a way of telling patients how to stop smoking, she does it in a, not in a 'well you should stop smoking', that kind of way. She'll say 'have you ever thought about giving it up. You know it would improve your chest a bit'. And I've seen [the nurse do it] more in a non-lecturey basis, more of a, 'have you ever thought about it?' Relaxed, warmer manner. So I'm not lecturing you, it's your choice. You know it's bad for you."* (Focus group 4)

For peer review only - http://bmjopen.bmj.com/site/about/guidelines.xhtml

DISCUSSION

Our study was the first to explore the influences on lung cancer symptom presentation in high risk, highly deprived groups across three nations of the UK. Preferences for an intervention targeted at high-risk groups were ascertained through focus groups. We found evidence from the interviews and focus groups that individuals who are at high risk for lung cancer tend to be preoccupied by maintaining health in the short term. Prioritising the daily management of their lung condition led to avoiding consideration of longer term health problems such as lung cancer, to gain a sense of control over health in the context of difficult personal circumstances. Health beliefs were found to underpin behaviour in relation to medical help seeking, where perceptions of 'inevitable but curable' chest infections led to immediate help seeking. However, 'inevitable but incurable' lung cancer led to inaction when faced with potentially serious symptoms and anticipated refusal of treatment. Interview participants felt that the relationship with the healthcare professional was key when considering medical help seeking. The importance of the relational interaction between provider and patient was mirrored in the focus groups, where participants felt that a non-judgemental intervention facilitator was important. Multi-faceted community-based interventions, away from the traditional healthcare setting, were preferred by participants.

Previous empirical studies report prolonged lung cancer symptom presentation due to misattribution[5,13,15-26,33, 42] and in our study, we found evidence that participants normalised their symptoms indicative of lung cancer to smoking habit, and lung and other comorbid conditions. In contrast to previous studies that report haemoptysis as a facilitator to prompt medical help seeking[13,25,27,43-45], current participants with experience of haemoptysis reported described avoidant coping, and normalisation when blood was noticed. Dismissal and normalisation of haemoptysis may be specific to socioeconomically deprived groups. Our highly deprived sample reported daily struggles with complex physical and mental health needs, and with the challenges associated with living on no or limited income. Previous studies in socioeconomically deprived communities report that in the context of competing life demands, health was dealt with reactively and with low priority[46,47].

Fear of being ineligible for treatment due to lifestyle has not been well described in studies with lung cancer patients or those at high risk[44,48]. In contrast, participants in the current study described feeling disentitled to medical services in the context of their lifestyle and circumstances. The underlying concept of health service candidacy (perceived eligibility for healthcare)[47] may explain why participants felt unworthy of seeking medical help and is likely to be of particular importance in

For peer review only - http://bmjopen.bmj.com/site/about/guidelines.xhtml

our highly deprived sample. In addition to challenging life circumstances, interview and focus group participants reported fear of being judged and ignored by health professionals due to their smoking habit or perceived social standing, contributing to feelings of unworthiness. Participants reported the desire to be a model patient and to not waste valuable GP time, which influenced medical help seeking. Although the desire to be a 'good citizen' has previously been reported[24,25], to our knowledge, the current study was the first to explore perceptions of appropriate consultation behaviour in a highly deprived sample. Our emerging findings related to candidacy, combined with the desire to exhibit 'good' consultation behaviour, may contribute to normalisation of symptoms previously regarded as serious and therefore discourage help seeking. Consequently, disadvantaged populations are likely to focus on health in the short term, and ignore longer-term health issues which may lead to advanced stage lung cancer diagnosis.

We found that participants held seemingly contradictory views on their lung cancer susceptibility, reporting scepticism about the causal role of smoking in lung cancer alongside perceived inevitability of lung cancer. Beliefs about the link between smoking and lung cancer may reflect societal stigma towards smoking, where participants downplay the negative effects of smoking, possibly to legitimise medical help seeking for symptoms considered related to smoking. Perceived inevitability of lung cancer is likely to reflect high levels of exposure in social networks where there is high incidence and poor outcomes of lung cancer [10], which should minimise normalisation of lung cancer symptoms and prompt help seeking[49]. Contrary to previous studies, current participants reported feeling that lung cancer was inevitable while simultaneously normalising and ignoring haemoptysis, possibly due to a combination of high fear and fatalism about lung cancer, difficult life circumstances and low perceived health service candidacy. High-risk individuals who believe that they cannot legitimately seek medical help because of their former or current lifestyle may therefore be resigned to the prospect of developing lung cancer.

A major strength of this study was the rigorous sampling procedure. We screened postcodes to ensure participants resided in the lowest quintile of deprivation, and measured multiple additional indicators of deprivation. Individual and area level indicators confirm that our sample was highly deprived, for instance most were unemployed and seeking benefits, and rented social housing. In addition, we recruited participants with no previous diagnosis of lung cancer, without mention of lung cancer until discussed by participants during the interview, or at the end of the interview. These recruitment and interview procedures meant we were able to explore previous and anticipated lung cancer symptom presentation in those who were symptomatic or asymptomatic. This strategy was

For peer review only

employed to overcome the methodological limitations associated with studying either retrospective or anticipated symptom presentation in isolation[15]. However, our qualitative study was unable to establish causal links between barriers and help seeking, nor can we generalise or compare the findings to high socioeconomic groups; instead, we conducted an in-depth study to explore how best to engage high risk, highly deprived individuals in early lung cancer diagnosis. Although we carefully sampled participants and collected additional demographic measures to validate our sampling frame, some GP practices were asked to recruit by specific smoking status rather than the whole range of smoking status, potentially introducing bias to our sample. In addition, we were unable to conduct a focus group in Scotland due to low response, which is a potential limitation of the study. Finally, focus group participants were recruited opportunistically, with the potential that participants were more favourably disposed to an intervention.

Practice and policy implications

With a comorbid lung condition and smoking history, those who are high risk for lung cancer will, in the main, be symptomatic. To avoid normalisation of symptoms, it is important to highlight the significance of changing and multiple symptoms. High risk individuals should be empowered to seek timely medical help and made to feel welcome, not judged or blamed for their current or former lifestyle. For instance, interventions targeted at disadvantaged populations could be conducted outside of the traditional healthcare setting. Our findings highlight the importance of an intervention where participants would be invited to attend, as opposed to presenting to the GP surgery, in order to eliminate concerns about wasting GP time and legitimise their attendance. Community based interventions have the potential to harness the relational aspects of help seeking, through interventions led by non-judgemental and welcoming facilitators. It is possible that previous mass media and social marketing lung cancer awareness interventions report low campaign reach to deprived groups [34,35] in part because they were not designed to motivate help seeking through intensive approaches to build trusting relationships and confidence. More research is required to understand how the relational aspects of help seeking could be operationalised in an intervention.

Over half of the current sample described mental health problems and/or difficult current or former life circumstances. Intervention developers and healthcare professionals in highly deprived communities should be aware of these wider social and contextual factors; they should receive training to recognise such circumstances and know how to appropriately signpost. Finally, we suggest that the current UK health system may encourage patients with a lung condition to focus on

For peer review only - http://bmjopen.bmj.com/site/about/guidelines.xhtml

short term management of their condition. GP prescribing of antibiotics and the use of rescue packs (prescribed antibiotics for storage at home in the event of an exacerbation) may inadvertently reinforce patients to detect and act on symptoms of a chest infection[50]. There is potential that this current standard of care could be adapted to educate and encourage patients with a lung condition to detect symptoms of lung cancer, thereby shifting the focus to longer term health. More research is required to understand how to motivate highly deprived groups to consider health in the long term, while recognising the wider social determinants of health[51].

Conclusion

The challenges of living in an area of deprivation with social exclusion issues, combined with fear of judgement by health professionals, contribute to avoidance and ignoring of lung cancer symptoms. Multi-faceted community based interventions are required to highlight lung cancer symptoms, the importance of early diagnosis and empower people who are high risk for lung cancer to seek timely medical help.

For peer review only

For peer review only - http://bmjopen.bmj.com/site/about/guidelines.xhtml

Additional information

Ethics approval and consent to participate

The study received ethical approval from Southampton Central- Hampshire A Research Ethics Committee (16/SC/0589). Written informed consent was obtained from all participants who took part in the study.

Consent for publication

Not applicable

Data sharing

Qualitative data is not publicly available for this study to maintain participant confidentiality.

Competing interests

The authors confirm that there are no conflicts of interest.

Funding

This work was supported by Cancer Research UK (grant reference number: C16377/A22034).

Author contributorship

All authors (GMM, JH, KH, PM, RDN, GN, ST, AMT, KB) designed the study. GMM and JH conducted, coded and analysed the interviews and focus group data. All authors contributed to the interpretation of data. GMM drafted the manuscript and all authors contributed to the review and editing of the manuscript. All authors read and approved the final manuscript.

Acknowledgements

We would like to thank everyone who helped with recruitment for this study, and all the participants who took part in the study; without these individuals this study would not have been possible. We would like to thank the Advisory Group (Anthony Byrne, Gareth Collier, Adrian Edwards, Dyfed Huws, David Lewis, Maura Matthews and Fiona Walter) and the study Administrator Lucy Watkins, for their ongoing support and advice throughout the study.

For peer review only - http://bmjopen.bmj.com/site/about/guidelines.xhtml

For peer review only

References

1. Bray F, Ferlay J, Soerjomataram I, Siegel RL, Torre LA, Jemal A. Global cancer statistics 2018: GLOBOCAN estimates of incidence and mortality worldwide for 36 cancers in 185 countries. CA: A Cancer Journal for Clinicians.0(0).

2. Allemani C, Matsuda T, Di Carlo V, Harewood R, Matz M, Nikšić M, et al. Global surveillance of trends in cancer survival 2000–14 (CONCORD-3): analysis of individual records for 37 513 025 patients diagnosed with one of 18 cancers from 322 population-based registries in 71 countries. The Lancet. 2018;391(10125):1023-75.

3. McPhail S, Johnson S, Greenberg D, *et al.* Stage at diagnosis and early mortality from cancer in England. *BJC* 2015;**112**(1):108-15. doi: 10.1038/bjc.2015.49.

4. Barrett J, Hamilton W. Pathways to the diagnosis of lung cancer in the UK: a cohort study. *BMC Med Prac*. 2008;**9**(31). doi: 10.1186/1471-2296-9-31.

5. Mitchell ED, Rubin G, Macleod U. Understanding diagnosis of lung cancer in primary care: qualitative synthesis of significant event audit reports. *BJGP* 2013;**63**(606):37-46. doi: 10.3399/bjgp13X660760.

6. Lyratzopoulos G, Neal RD, Barbiere J, *et al*. Variation in number of general practitioner consultations before hospital referral for cancer: findings from the 2010 National Cancer Patient Experience Survey in England. *Lancet Oncol* 2012;**13**(4):353-65. doi: 10.1016/S1470-2045(12)70041-4.

7. Iyen-Omofoman B, Tata L, Baldwin DR, *et al*. Using socio-demographic and early clinical features in general practice to identify people with lung cancer earlier. *Thorax* 2012;**68**:451-9.

8. Hippsley-Cox J, Coupland C. Identifying patients with suspected lung cancer in primary care: derivation and validation of an algorithm. *BJGP* 2011;**61**(592):715-23. doi: 10.3399/bjgp11X606627.

9. NICE. NICE guidelines for lung cancer: diagnosis and management. *NICE*. 2015. Avaliable at: https://www.nice.org.uk/guidance/cg121 [accessed 04.06.2018].

10. National Cancer Registration and Analysis Service. Cancer by deprivation in England: 1996-2011. 2014. Avaliable at: http://www.ncin.org.uk/about_ncin/cancer_by_deprivation_in_england [accessed 04.06.2018].

11. Sekine Y, Katsura H, Koh E, *et al. Early* detection of COPD is important for lung cancer surveillance. *Eur Resp J* 2012;**39**(5):1230-40. doi: 10.1183/09031936.00126011.

12. Malhotra J, Malvezzi M, Negri E, *et al*. Risk factors for lung cancer worldwide. *Eur Resp J* 2016;**48**:899-902. doi: 10.1183/13993003.00359-2016.

For peer review only

For peer review only - http://bmjopen.bmj.com/site/about/guidelines.xhtml

13. Smith SM, Campbell NC, MacLeod U *et al*. Factors contributing to the time taken to consult with symptoms of lung cancer: a cross-sectional study. *Thorax* 2009;**64**(6):523-31.

14. Friedemann-Smith C, Whitaker K, Winstanley K, Wardle J. Smokers are less likely than non-smokers to seek help for a lung cancer 'alarm' symptom. *Thorax* 2016;**71**(7). doi.org/10.1136/thoraxjnl-2015-208063

15. Brouselle A, Breton M, Benhadj L *et al*. Explaining time elapsed prior to cancer diagnosis: patients' perspectives. *BMC Health Serv Res* 2017;**17**(1):448. doi: 10.1186/s12913-017-2390-1.

16. McLachlan S, Mansell G, Sanders T *et al*. Symptom perceptions and help-seeking behaviour prior to lung and colorectal cancer diagnoses: a qualitative study. *Fam Pract*. 2015;**32**(5):568-77. doi: 10.1093/fampra/cmv048.

17. Caswell G, Seymour J, Crosby V *et al*. Lung cancer diagnosed following an emergency admission: exploring patient and carer perspectives on delay in seeking help. *Sup Care Cancer* 2017;**25**(7):2259-66. doi: 10.1007/s00520-017-3633-8.

18. Brindle L, Pope C, Corner J, *et al*. Eliciting symptoms interpreted as normal by patients with early-stage lung cancer: could GP elicitation of normalised symptoms reduce delay in diagnosis? Cross-sectional interview study. *BMJ Open* 2012;**2**(6):1977. doi.org/10.1136/bmjopen-2012-001977.

19. Lyratzopoulos G, Vedsted P, Singh H. Understanding missed opportunities for more timely diagnosis of cancer in symptomatic patients after presentation. *BJC* 2015;**112**(1):84-91. doi: 10.1038/bjc.2015.47.

20. Emery JD, Walter FM, Gray V *et al.* Diagnosing cancer in the bush: a mixed-methods study of symptom appraisal and help-seeking behaviour in people with cancer from rural Western Australia. *Fam Pract* 2013;**30**(5):541-50. doi: 10.1093/fampra/cms087.

21. Birt L, Hall N, Emery J *et al*. Responding to symptoms suggestive of lung cancer: a qualitative interview study. *BMJ Open* 2014;**11**(1);doi: 10.1136/bmjresp-2014-000067.

22. Chatwin J, Sanders C. The influence of social factors on help-seeking for people with lung cancer. *Eur J Cancer Care*. 2013;**22**(6):709-13. doi: 10.1111/ecc.12078.

23. Carter-Harris L. Lung Cancer Stigma as a Barrier to Medical Help-Seeking Behavior: Practice Implications. *J Am Assoc Nurse Pract* 2015;**27**(5):240-5. doi: 10.1002/2327-6924.12227.

24. Tod A, Craven J, Allmark P. Diagnostic delay in lung cancer: a qualitative study. *J Adv Nurs* 2008;**61**(3):336-43. doi: 10.1111/j.1365-2648.2007.04542.x.

25. Tod A, Joanne R. Overcoming delay in the diagnosis of lung cancer: a qualitative study. *Nurs Standard* 2010;**24**(31):34-43. doi: 10.7748/ns2010.04.24.31.35.c7690.

26. Corner J, Hopkinson J, Roffe L. Experience of health changes and reasons for delay in seeking care: a UK study of the months prior to the diagnosis of lung cancer. *Thorax* 2006;**60**:1382-91.

For peer review only

27. Braybrook DE, Witty KR, Robertson S. Men and lung cancer: a review of the barriers and facilitators to male engagement in symptom reporting and screening. *J Men's Health* 2011;**8**(2):93-9. https://doi.org/10.1016/j.jomh.2011.03.002.

28. Chapple A, Ziebland S, McPherson A. Stigma, shame, and blame experienced by patients with lung cancer: qualitative study. *BMJ* 2004;328:1470. doi.org/10.1136/bmj.38111.639734.7C.

29. Murray SA, Kutzer Y, Habgood E *et al*. Reducing barriers to consulting a General Practitioner in patients at increased risk of lung cancer: a qualitative evaluation of the CHEST Australia intervention. *Fam Pract* 2017;**34**(6):740-6. doi.org/10.1093/fampra/cmx057.

30. Marlow LA, Waller J, Wardle J. Does lung cancer attract greater stigma than other cancer types? *Lung Cancer* 2015;88(1):104-7. doi: 10.1016/j.lungcan.2015.01.024.

31. Quaife S, McEwen A, James S, Wardle J. Smoking is associated with pessimistic and avoidant beliefs about cancer: results from the International Cancer Benchmarking Partnership. *BJC*. 2015 **112**(11):1799-804. doi: 10.1038/bjc.2015.148.

32. Quaife S, Marlow L, McEwen A, Wardle J. Attitudes towards lung cancer screening in socioeconomically deprived and heavy smoking communities: informing screening communication. *HEX* 2017;**20**(4):563-73. doi: 10.1111/hex.12481.

33. Mor V, Masterson-Allen S, Goldberg R, *et al*. Pre-diagnostic symptom recognition and help seeking among cancer patients. *J Community Health* 1990;**15**(4):253-66.

34. Moffat J, Bentley A, Ironmonger L, Boughey A, Radford G, Duffy S. The impact of national cancer awareness campaigns for bowel and lung cancer symptoms on sociodemographic inequalities in immediate key symptom awareness and GP attendances. British Journal Of Cancer. 2015;112:S14.

35. Kennedy MPT, Cheyne L, Darby M, Plant P, Milton R, Robson JM, et al. Lung cancer stage-shift following a symptom awareness campaign. Thorax. 2018.

36. Smith S, Fielding S, Murchie P, Johnston M, Wyke S, Powell R, et al. Reducing the time before consulting with symptoms of lung cancer: a randomised controlled trial in primary care. The British Journal of General Practice. 2013;63(606):e47-e54.

37. Tong A, Sainsbury P, Craig J. Consolidated criteria for reporting qualitative research (COREQ): a 32-item checklist for interviews and focus groups. *Int J Quality Health Care* 2007;**19**(6):349-57.

38. Leventhal H, Brissette I, Leventhal E. The common-sense model of self-regulation of health and illness. In: The Self-regulation of Health and Illness Behaviour. *Routledge*. 2003:42-65.

39. Francis JJ, Johnston M, Robertson C *et al*. What is an adequate sample size? Operationalising data saturation for theory-based interview studies. *Psych Health* 2010;**25**(10):1229-45. doi: 10.1080/08870440903194015.

40. Ritchie LD, Lewis J. Qualitative Research Practice: A Guide for Social Science Students and Researchers. Sage: London. 2003.

For peer review only - http://bmjopen.bmj.com/site/about/guidelines.xhtml

41. Ward D.J., Furber C., Tierney S., Swallow V. (2013) Using Framework Analysis in nursing research: a worked example. *Journal of Advanced Nursing* 69(11), 2423–2431. 10.1111/jan.12127

42. Anderson R, Paarup B, Vedsted P, *et al.* 'Containment' as an analytical framework for understanding patient delay: a qualitative study of cancer patients' symptom interpretation processes. *Soc Sci Med* 2010;**71**(2):378-85. doi: 10.1016/j.socscimed.2010.03.044.

43. Chan W, Clark A, Dernedde U *et al*. Symptoms, delay to presentation and survival in lung cancer. *Thorax* 2016;**71**(3):80-1.

44. Corner J, Hopkinson J, Fitzsimmons D, *et al*. Is late diagnosis of lung cancer inevitable? Interview study of patients' recollections of symptoms before diagnosis. *Thorax* 2005;**60**:314-9. doi.org/10.1136/thx.2004.029264.

45. Dobson C, Russell A, Brown S, Rubin G.The role of social context in symptom appraisal and help-seeking among people with lung or colorectal symptoms: A qualitative interview study. *Eur J Cancer Care* 2018;**27**(2):e12815. doi: 10.1111/ecc.12815.

46. Smits S, McCutchan G, Wood F *et al*. Development of a Behavior Change Intervention to Encourage Timely Cancer Symptom Presentation Among People Living in Deprived Communities Using the Behavior Change Wheel. *Annals Beh Med*. 2016;**52**(6):474-488. doi: 10.1007/s12160-016-9849-x.

47. Dixon-Woods M, Cavers D, Agarwal S *et al*. Conducting a critical interpretive synthesis of the literature on access to healthcare by vulnerable groups. *BMC Med Res Method* 2006;**6**(35). doi.org/10.1186/1471-2288-6-35.

48. Smith SM, Murchie P, Devereux G, Johnston M, Lee AJ, Macleod U, et al. Developing a complex intervention to reduce time to presentation with symptoms of lung cancer. The British Journal of General Practice. 2012;62(602):e605-e15.

49. Macdonald S, Watt G, Macleod U. In search of the cancer candidate: can lay epidemilogy help? *Soc Health Illness* 2013;**35**(4):575-91. doi: 10.1111/j.1467-9566.2012.01513.x.

50. NICE. NICE guidelines for lung cancer: diagnosis and management. *NICE*. 2015. Avaliable at: https://www.nice.org.uk/guidance/cg121 [accessed 04.06.2018].

51. Barton H, Grant M. A health map for the local human habitat. *J Royal Soc Promotion Health.* 2006;**126**(6):252-3.

For peer review only - http://bmjopen.bmj.com/site/about/guidelines.xhtml

Supplementary File 1. Consolidated criteria for reporting qualitative studies (COREQ): 32-item checklist with page numbers to indicate section of the article [34]

**Checklist item**	**Questions to consider**	**Page number in article**
**Domain 1: Research team and reflexivity**		
Personal Characteristics		
1. Interviewer/facilitator	Which author/s conducted the interview or focus group?	8
2. Credentials	What were the researcher's credentials? *E.g. PhD, MD*	8
3. Occupation	What was their occupation at the time of the study?	8
4. Gender	Was the researcher male or female?	8
5. Experience and training	What experience or training did the researcher have?	8
Relationship with participants		
6. Relationship established	Was a relationship established prior to study commencement?	7
7. Participant knowledge of the interviewer	What did the participants know about the researcher? e.*g. personal goals, reasons for doing the research*	7
8. Interviewer characteristics	What characteristics were reported about the interviewer/facilitator? e.g. *Bias, assumptions, reasons and interests in the research topic*	8
**Domain 2: study design**		
Theoretical framework		
9. Methodological orientation and Theory	What methodological orientation was stated to underpin the study? *e.g. grounded theory, discourse analysis, ethnography, phenomenology, content analysis*	8-9
Participant selection		
10. Sampling	How were participants selected? *e.g. purposive, convenience, consecutive, snowball*	5-6
11. Method of approach	How were participants approached? e.*g. face-to-face, telephone, mail, email*	6-7
12. Sample size	How many participants were in the study?	5, 6, 10, 16
13. Non-participation	How many people refused to participate or dropped out? Reasons?	9
Setting		
14. Setting of data collection	Where was the data collected? e.*g. home, clinic, workplace*	8
15. Presence of non-participants	Was anyone else present besides the participants and researchers?	8
16. Description of sample	What are the important characteristics of the sample? *e.g. demographic data, date*	9-10, 16
Data collection		
17. Interview guide	Were questions, prompts, guides provided by the authors? Was it pilot tested?	Appendix 2-4

For peer review only

18. Repeat interviews	Were repeat interviews carried out? If yes, how many?	6
19. Audio/visual recording	Did the research use audio or visual recording to collect the data?	6, 8
20. Field notes	Were field notes made during and/or after the interview or focus group?	8
21. Duration	What was the duration of the interviews or focus group?	8
22. Data saturation	Was data saturation discussed?	7
23. Transcripts returned	Were transcripts returned to participants for comment and/or correction?	
**Domain 3: analysis and findings**		
Data analysis		
24. Number of data coders	How many data coders coded the data?	8
25. Description of the coding tree	Did authors provide a description of the coding tree?	8-9
26. Derivation of themes	Were themes identified in advance or derived from the data?	8-9
27. Software	What software, if applicable, was used to manage the data?	8-9
28. Participant checking	Did participants provide feedback on the findings?	8-9
Reporting		
29. Quotations presented	Were participant quotations presented to illustrate the themes / findings? Was each quotation identified? e.g. *participant number*	14-16, 18-19
30. Data and findings consistent	Was there consistency between the data presented and the findings?	11-19
31. Clarity of major themes	Were major themes clearly presented in the findings?	11-19
32. Clarity of minor themes	Is there a description of diverse cases or discussion of minor themes?	11-19

For peer review only

Supplementary File 2: Interview topic guide

Interview prologue

- Introduce the researcher, ensure the participant is comfortable.
- Explain purpose of the interview:
    - *"We are interested in lung health.*
    - *We would like to know how people cope with lung symptoms and how they decide if they should go to the doctor or not.*
    - *Some of the questions I ask during the interview will be about smoking. I'm not here to tell you to stop smoking; I'm just interested if you have any experiences of smoking.*
    - *I'm not a clinician so I can't give you any advice on symptoms, but I can tell you where you can go to get help"*

- Check understanding of interview purpose, role of researcher, and what will happen in the interview. Give opportunity for questions.
- Partners or family members will be welcome to join the interviews to explore relational influences on lung symptom awareness and help seeking, and the interview topic guide will be adapted accordingly.
- After establishing what is understood about the study, and answering any questions, explain that the interview will be recorded. Obtain consent for the interview and for the recording. If not already done, set up and switch on the recording equipment while the participant signs the consent form.

Note to interviewer: the grey boxes signify the key topics to be explored during the interview. The questions listed below are examples of suggested prompts for each topic. You are not required to read these verbatim unless specified.

For peer review only

Overall aim of the qualitative interview:
-To explore how people with a history of smoking and respiratory conditions interpret and act on new or changing lung symptoms (how people cope with lung symptoms and how they decide to go to the doctor with symptoms)
-To explore the influences of perceived risk, fear, shame, stigma, family and friends on lung symptom presentation

1. Experiences of lung condition

Establish what lung condition the participant has, how the lung condition affects them, the types of symptoms they experience and how they are usually managed.

Rationale: introduce the participant to the format of a qualitative interview and make the participant feels comfortable. Establish details of their lung condition.

Potential prompts:

- Can you tell me about your lung condition?
- Tell me about your experiences with X lung condition.
  - How long have you had X condition?
  - What sort of symptoms do you experience?
  - How long do the symptoms usually last?
- What do you think causes these symptoms?
- How do you usually manage your symptoms?
- How are your symptoms usually managed/ treated by your healthcare professional?
  - How effective do you think this is?

For peer review only

2. Symptom attribution task

Use as a tool for prompting an in-depth understanding of symptom attributions and confidence to interpret new or changing symptoms.
Include discussion around previous symptom experiences including: what action was taken, if and who they sought medical help from. Explore how their lung condition and smoking history might influence symptom attributions (i.e. do these mask symptoms?) and symptom presentation.

Rationale: to explore lung symptom attribution; confidence in recognising and articulating symptoms; previous symptom experience; planning when/how to act on symptoms; influence of smoking history on perceptions of lung symptoms

Ask participant to order symptom cards from symptoms that they would seek medical help quickest for and those they would seek help slowest for (arrow and boxes below will be stretched to A4 size, see page 10). Ask participant to write numbers on the symptom cards. 1= first, 11=last. If they change the order of the symptom, ensure the previous number is crossed out and the new number written on card. Write PID on the back of each symptom card. Take photos of the task.

Say the following phrase verbatim: "We know that people decide go to the doctor at different times with symptoms. I'm going to show you some pieces of card with symptoms on. I'd like you to rank them from the ones that you would go to the doctor with first through to the last on this sheet of paper."

Cut individual symptom cards (see page 12) for the following symptoms. Please rotate the order:

- Coughing up blood
- A cough that doesn't go away
- A long standing cough that gets worse
- Pain in your chest or shoulder
- Persistent breathlessness
- Persistent chest infections
- Persistent tiredness or lack of energy
- Loss of appetite or unexplained weight loss
- Ache or pain when breathing or coughing
- A hoarse voice
- Changes in the appearance of your fingers, such as becoming more curved or their ends becoming larger

See the **glossary of terms at the end of the topic guide** with standardised definitions and additional explanation of symptoms. If the participant does not understand what each symptom means, ask them what they think it means, then refer to the glossary of terms.

First to go to the doctor

↑

Last to go to the doctor

If the participant asks the interviewer what these symptoms are, first **ask the participant what they think they are**. If they ask again either suggest to move on and discuss what they could be after the interview (if appropriate) or say the symptoms have been taken from the NHS website for lung cancer.

For peer review only

Note to interviewer: Aim to the complete the ordering of symptoms in the task first, and then ask questions in this section. However, if any of the following (2a, 2b, 2c, 2d) if bought up spontaneously by the participant whilst they are doing the card sorting exercise then explore these issues at that point. Then return to the card sorting exercise, followed by questions in the following section (2a, 2b, 2c, 2d).

Topics and prompts for symptom attribution task

Once the symptoms have been ordered, ask:

- Can you tell me why you put x first?
- Can you tell me why you put x last?

For peer review only

2a. Symptom experience

To understand if the participant has experienced the symptom previously- what did they attribute the symptom to? What did they do? If they have not experienced the symptom before, what would they hypothetically do if they were to experience symptom in the future?

- Which of these symptoms have you had before?
    - What did you think the symptom(s) was/were?
    - Which symptom(s) did you go to the doctor with?
        - Why did you decide to go to the doctor with these symptoms?
        - What did the doctor say?
    - Which symptoms did you decide to not go to the doctor with?
        - Why did you decide not to go to the doctor with these symptoms?
- For the other symptoms we have not talked about, if you developed any of them what would you do?
    - How long do you think it would take you to go to the doctor with these symptoms?
    - Can you tell me why it would take you this amount of time to go to your doctor with this symptom?
    - What you think these symptoms could be?
- Are there any symptoms you wouldn't go to the doctor with?
    - Can you tell me why you wouldn't go to the doctor with these symptoms?

2b. Detecting change

Explore how the participant has/would notice new or changing symptoms and their confidence to detect new and changing symptoms. Ask this section generally, but if the participant is struggling then ask them about confidence to detect new and changing symptoms in the context of previous symptoms experiences. i.e. earlier you mentioned you felt breathless for a long time, how confident did you feel to know that this was a new or changing symptom?

- How would you normally tell if any lung symptoms have changed/ are new/ or unusual for you?
    - What would you do if you think you noticed a change in a symptom?
    - How confident would you feel in recognising a change in your usual symptom(s)?
    - How confident would you feel recognising new symptoms?
    - How would you notice a change in any of these symptoms?

2c. Barriers

Explore the influences of barriers and social influences on lung symptom presentation behavior

- Can you think of anything that would influence your decision to go to the doctor with any of these symptoms?
    - Probe barriers/enablers to going to the GP with a symptom:
        - Transport
        - Long wait times/ appointment policies
        - Worry about wasting the doctors time i.e. Some people have told us that they don't go to the doctor as they worry about wasting the doctors time. What do you think about this?
        - The influence of partner/ social influences (who suggested you go/ don't go to the doctor?)
        - How confident would you feel talking to the doctor about these symptoms?
            - How do you feel when you are talking to the doctor?

2d. Smoking

Explore the influence of smoking habit on the ability to notice new or changing symptoms. If/how smoking habit acts as a barrier to seeking medical help. If vaping comes up say something like- that is really interesting, do you mind if I ask you some questions later about vaping if we have time? If they vape then still explore the influence of past smoking on symptom perceptions.

- Would you expect to have any of the symptoms we have talked about because you smoke or used to smoke?
    - Thinking about smoking, how do you think your [past] smoking would affect how you notice if a symptom has changed or is new/ unusual for you?
    - Does your doctor ever discuss smoking with you?
        - How does this make you feel?

For peer review only

▪

3. Lung cancer

If appropriate, explore lung cancer knowledge, beliefs about lung cancer and perceived risk here. If bought up spontaneously by the participant earlier in the interview, explore lung cancer then (if appropriate) Confidence to detect a lung cancer symptom. The influences of smoking and perceived risk, shame and associated with lung cancer.

Rationale: to explore lung cancer symptom awareness; to explore perceived risk of lung ca emotional consequences such as fear, fatalism blame; perceived causes and effectiveness of e detection and treatment; the influence of smol history on the formation of beliefs about lung c

If it is OK with you, I'm going to ask you some questions about lung cancer. If you feel uncomfortable with any of my questions, please let me know and we can move on.

Potential prompts:

- Earlier you mentioned some of the symptoms that could be lung cancer. Can you think of any other symptoms that you think might be lung cancer?
- What else do you know about lung cancer?
- Can you tell me a little more about what you think about lung cancer as a disease?
    - Where do you think that feeling comes from?
    - What do you think other people's views are on lung cancer?
- What do you think causes lung cancer? S
    - If yes and appropriate: How does that make you feel as a [past] smoker?
    - If appropriate: do you ever worry about lung cancer because you [used to] smoke?
    - How does that make you feel?
    - Is there anything in particular that makes you think your risk is high or low?
- How confident would you feel in recognising a symptom that could be lung cancer?
    - How does your [past] smoking affect your confidence in recognising a change in your body that could be lung cancer?
- What would you do if you had a symptom that you thought was lung cancer?

4. Social influences

Explore influences of social networks on help seeking behavior and basis of cancer beliefs.

Rationale: to explore social norms, influences and stigma around help seeking; people who they know who have had lung cancer and the effects on beliefs and perceptions; how social networks might influence help-seeking

- If you had a symptom you thought might be lung cancer, would you tell anyone about it?
  - Who would you tell?
  - Why would you tell that person?
  - What do you think they would say to you if you told them about a symptom?
  - Would they encourage/discourage you to visit your doctor?
- Has anyone ever noticed a symptom of yours and suggest that you go to the doctor?
  - Probe: who/ what happened.
- We know that lung cancer isn't as common as other types of cancer like breast cancer. Do you know anyone who has ever been diagnosed with lung cancer?
- Can you tell me about the effects that [person] having lung cancer has had on you?
  - How has this affected your views about lung cancer?

5. Closing questions

Final questions to end on a positive note

Rationale: to end the interview on a positive note

- If you were to offer anyone some advice on lung symptoms, what would it be?
- If the NHS were to make some changes to the services they offer to help people with lung symptoms get seen quicker, what would you suggest they change?
- Can you tell me about a positive experience of going to the doctor?

I know we've talked about a lot of things today, but would you like to tell me about anything else that we haven't talked about?

For peer review only

Debrief

- Summarise the interview and address any questions or concerns.
- Check whether it is ok to contact them if there is anything that needs to be clarified after listening back to the conversation.
- Ensure that they know how to contact us for further help/information/to add further information
- Thank them for their time and give them the gift voucher.
- If the participant discloses symptoms during the interview suggest they seek medical help from their GP. Offer lung cancer leaflet and site specific helpline numbers.
- Have stop smoking service details available if they request it
- Offer to provide a summary of study findings

Helpline numbers

Wales

- Tenovus Cancer Care support line on 0808 808 1010. The support line is open 8am-8pm, 7 days a week. Calls are free from a BT landline.

England and Scotland

- British Lung Foundation helpline on 03000 030555. This helpline is open 9am-5pm Monday to Friday. Calls cost the same as a local call.
- Roy Castle Lung Cancer Foundation helpline on 0333 323 7200. This helpline is open 9am-5pm Monday to Friday. Calls are free from a BT landline.

For peer review only

Symptoms in the symptom task:

**Coughing up blood**
**A cough that doesn't go away**
**A long standing cough that gets worse**
**Pain in your chest or shoulder**
**Persistent breathlessness**
**Persistent chest infections**
**Persistent tiredness or lack of energy**
**Loss of appetite or unexplained weight loss**
**Ache or pain when breathing or coughing**
**A hoarse voice**
**Changes in the appearance of your fingers, such as becoming more curved or their ends becoming larger**

For peer review only - http://bmjopen.bmj.com/site/about/guidelines.xhtml

Glossary of terms

Coughing up blood

If you notice that there is some blood coming up when you cough

A cough that doesn't go away

If you have a nagging cough that just doesn't seem to go away

A long standing cough that gets worse

You have had a cough for a long time but you think that it might have got worse

Pain in your chest or shoulder

A sharp or achy pain in either your chest or shoulder, or both

Persistent breathlessness

You feel like you can't catch your breath or become out of breath when doing tasks you used to be fine with

Persistent chest infections

If you have had a few chest infections in a row and they don't seem to be getting better or keep coming back

Persistent tiredness or lack of energy

If you have been feeling tired for a while or just feel like you don't have any energy

Loss of appetite or unexplained weight loss

If you have been loosing weight without trying to and can't explain why or you just haven't been feeling up to eating the amount you normally would for a while

Ache or pain when breathing or coughing

If you have a sharp pain or achy feeling when you cough or breathe

A hoarse voice

A croaky or gravelly voice

Changes in the appearance of your fingers, such as becoming more curved or their ends becoming larger

If you notice that the ends of your fingers have changed shape

Persistent definition

Something that you notice you have had for a while and won't go away.

For peer review only

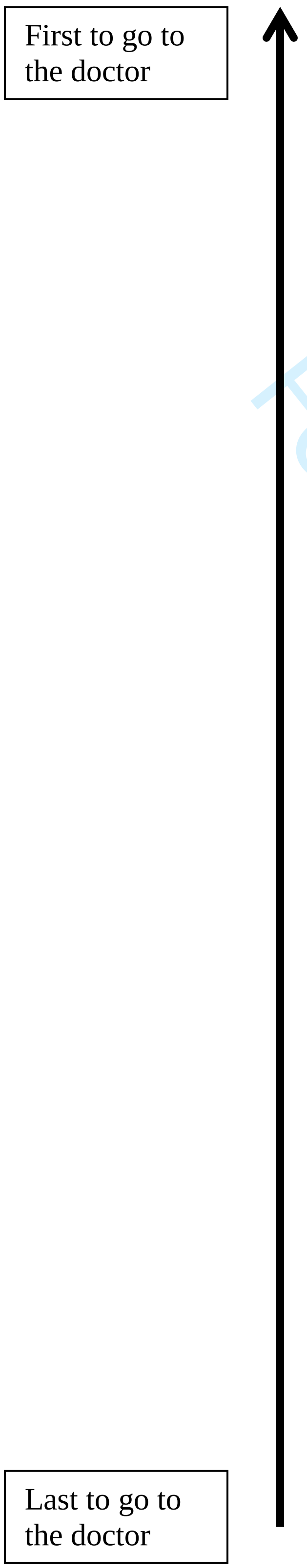

For peer review only

Supplementary File 3: HPCP focus group topic guide

Focus group aim: To explore the needs and preferences from members of the public and local stakeholders (healthcare professionals/ community partners) for an intervention to support earlier lung cancer detection and diagnosis, targeted at high risk, harder to reach groups (over 40's, who are current/former smokers, living in areas of deprivation with serious lung comorbidity i.e. COPD).

Introduction

- Explain the aims of the focus group discussion. Emphasise that the focus group is not a test; we are interested in participants' preferences for an intervention for "lung health" to prompt earlier lung cancer symptom presentation, and how we could access people to take part in an intervention. All comments are welcomed: positive and negative.
    - 'We know that people who are high risk for lung cancer (people over the age of 40, living in deprived areas, who currently smoke or used to smoke and have a lung condition like COPD) are often diagnosed at a late stage, where treatment options are limited and a cure is less likely. This might be because they delay seeking medical advice with symptoms suggestive of lung cancer.
      We are considering if we should develop something that will give people who are high risk for lung cancer information about symptoms and when/ where they should go and seek medical help from.
      As healthcare professionals and people who work in deprived communities, we would like to know what you think about if we should do this. If you think this is a good idea, we would also like to know how best we can access this target group and invite them to take part in an intervention about lung health. Your ideas and suggestions are really valuable to us because of your wealth of experience, so all comments are welcomed (both positive and negative)'.
- Explain the voluntary nature of the study and that the focus group will be recorded with permission. If not already done, set up and switch on the recording equipment while participants sign the consent form.
- Before starting the focus group, remind participants about confidentiality and ask participants not to talk over each other. Go around the circle and ask participants to introduce themselves for the transcription.

For peer review only

Rationale: to explore whether the findings resonate with patients and members of the public in their local communitv

Part1. Phase 1 interview findings

Discussion of Phase 1 interview findings

Provide a short overview of key findings from Phase 1 interviews regarding barriers and enablers to lung cancer awareness and early symptom presentation in the target group. Ask the group to share their thoughts on the interview findings, and whether they resonate with them.

'We have been interviewing people across the UK who are high risk for lung cancer- people over the age of 40 who have a lung condition like COPD, who currently smoke or used to smoke and live in deprived areas. We wanted to understand how people think about symptoms of lung cancer, how and when people decide to go to the doctor with lung symptoms and the barriers to going to the doctor with these symptoms.

We found that people are completely fixated on detecting chest infections and look out for symptoms of a chest infection most days. They were really good at knowing when they had a chest infection and going to the doctor quickly to get antibiotics because they know it can be treated.

We also found that people tend to deny or ignore health problems that might affect them in the future, like lung cancer so may not go to the doctor with some serious symptoms. We think this is because they think that although lung cancer is inevitable, they also think that it cannot be cured so don't go and see the doctor

We want to develop something that can help people get these important symptoms of lung cancer seen to quicker by a medical professional, but we are not really sure how to do this. We would like to know what you think.'

- How can we do this?
- What do you think of these findings?
- To what extent do these findings resonate with you?

Rationale: to seek views on how to access the target group for an intervention and explore preferences for an intervention to support earlier presentation, including mode of delivery, target group, content and stop smoking information

Preferences on intervention content

- What sorts of things do you think people would like to know / what skills would they like to learn?
  - What do you think would be most useful to people?
  - What information would be the highest priority/most important for them?
  - Should we avoid any information?
  - Do you think it is best to focus on symptoms or health beliefs?

Preferences on intervention format

- How can we support people to seek medical help quickly with lung cancer symptoms? [what format]
    - Probe: a group one-off educational session to promote "lung health" in the local community; a leaflet/DVD; a lung health check; event in the community; posters in the local community
    - Do you think people want education or to learn 'skills for health'?
        - What sort of skills do you think people would like to know?
- Who do you think would be best to lead or facilitate an intervention about lung health/ encourage people to seek medical help quickly with lung cancer symptoms?
    - Probe: patient representative, lay advisor, community partner, healthcare professional (GP, practice nurse, smoking cessation counsellor, respiratory nurse specialist…?)
- When would be best to support people to manage their lung health?
    - What do you think about using the point at which they are diagnosed with a lung condition as a 'teachable moment' to do an intervention about lung cancer?
    - What about during a regular check-up appointment with the nurse i.e. six monthly COPD clinic reviews?
- What do you think about a brief intervention with health care professionals in addition to a public facing intervention?
    - What would be useful to you as healthcare professionals?
- The intention is that this intervention would be implemented across the UK. How do you think everything we have talked about today might need to differ based on where someone lives (i.e. different countries) or the person in the intervention?
    - How could we incorporate an intervention into the different models of care across regions/countries?
- How do you think this intervention could fit with other current or planned health promotion activities?

Accessing intervention participants

- Where could we approach our target group in an intervention about lung cancer/lung health?
- Probe: through community pharmacies, primary care, community nurses, existing groups for people with lung conditions?
    - Probe: on utilising family / social networks to encourage participation of MoP?
- Are you aware of any existing groups for people who fall into our target group?
    - What do you think about "piggybacking" onto these existing community groups or existing care plans with the nurse?
    - How do you think people would react to this?

Smoking cessation

- 'We know that people really don't like it when the doctor tells them to stop smoking and some people felt like they were treated differently by the doctors because they smoked. Sometimes this put people off going to the doctor with lung symptoms. On the other hand some people feel that it is only fair to be told to stop smoking. When we design something about lung cancer, we don't know if we should include something to help people to stop smoking or not.'
- What do you think about including stop smoking information in the intervention?
    - How do you think smokers might react to including stop-smoking information?
    - Can you think of ways we might be able to include stop-smoking information without putting smokers off?
    - What do you think about using signposting to stop smoking services instead of providing information about smoking cessation during the session?
- What do you think about using the intervention to cover things that are not related to lung health i.e. mental health or other factors that could be related to lung health?
    - How should we approach this?

Debrief

'Thank you for taking part in this study. We hope to use the findings from this focus group to develop an intervention based on the findings from the interviews and your recommendations to encourage earlier lung symptom presentation in those who are high risk for lung cancer. Anything you said will be treated as confidential. The voice-recoding will be stored securely. Any quotes used in published research will not have your name or anything that could identify you. Do you have any questions? [answer any questions] Here are my contact details if you have any further questions.'

For peer review only

Supplementary File 4: Members of the public focus group topic guide

Focus group aim: To explore the needs and preferences from members of the public and local stakeholders for an intervention to support earlier lung cancer detection and diagnosis. The intervention will be targeted at high risk, harder to reach groups (over 40's, who are current/former smokers, living in areas of deprivation with serious lung comorbidity i.e. COPD).

Introduction

- Explain the aims of the focus group. Emphasise that the focus group is not a test; we are interested in participants' preferences for an intervention for "lung health" to prompt earlier lung symptom presentation, and how we could access people to take part in an intervention. All comments are welcomed: positive and negative.
  - '*We know that some people sometimes might take a bit longer to go to the doctor with important lung symptoms. I am thinking about developing something that will highlight important lung symptoms, and encourage people to go to speak to someone who is medically trained about their symptoms. I would like to know what you think about best ways to do this. I would also like to know how you think we can find people for this. Your ideas and suggestions are really valuable to us, so all comments are welcomed (both positive and negative)*'.
- Explain the voluntary nature of the study and that the focus group will be recorded with permission. If not already done, set up and switch on the recording equipment while participants sign the consent form.
- Before starting the focus group, remind participants about confidentiality and ask participants not to talk over each other. Go around the circle and ask participants to introduce themselves for the transcription.

Discussion of Phase 1 interview findings

Rationale: to explore whether the findings resonate with patients and members of the public in their local community

Provide a short overview of key findings from Phase 1 interviews regarding barriers and enablers to lung cancer awareness and early symptom presentation in the target group. Ask the group to share their thoughts on the interview findings, and whether they resonate with them.

'We did some interviews across the UK with people who smoke or used to smoke and have a lung condition like COPD. We found that people look out for symptoms of a chest infection most days. They were really good at knowing when they had a chest infection and going to the doctor to get antibiotics. We also found that people try not to think about health problems that might affect them in the future, so may not go to the doctor with some symptoms that could be serious.

People really didn't like it when the doctor told them to stop smoking and some people felt like they were treated differently by the doctors because they smoked. Sometimes this put people off going to the doctor with lung symptoms.

We also found that people often had a favourite doctor at their GP practice and would wait up to three weeks for an appointment even if they thought a symptom was important. People don't like

For peer review only

going to doctors that they don't know because they felt that they did listen as much as their favourite doctor. But sometimes it was necessary to go to a doctor they don't know to get an appointment the same day.

We want to develop something that can help people get important lung symptoms seen to quicker by a medical professional, but we are not really sure how to do this or who should do this. We would like to know what you think.'

- What do you think of these findings?
- In what ways do you feel the same as what we found?
- In what ways do you feel different to what we found?

> ***Rationale:*** to seek views on how to access the target group for an intervention and explore preferences for an intervention to support earlier presentation, including mode of delivery, target group, content and stop smoking information

For peer review only

<u>Part 2. Needs and preferences for an intervention</u>

Preferences on intervention format

- In what ways could we support people to manage their lung health?
    - Probe: a group one-off educational session to promote "lung health" in the local community; a leaflet/DVD; a lung health check; event in the community; posters in the local community
- Who do you think would be best to lead an intervention about lung health?
    - Probe: lung cancer survivor, lay advisor, community partner, healthcare professional
- If a health care professional was to lead the intervention, who would be best to lead a lung health intervention?
- Who is your favourite healthcare professional?
- When would be best to support people to manage their lung health?
    - What about when someone is diagnosed with a lung condition?
    - What about in one of your regular check-up appointments with the nurse i.e. six-monthly COPD clinic review?

Preferences on intervention content

- **What would you like to know? OR what skills would you like to learn?**
    - What would be most useful or important for you?
    - Can you think of anything that we should avoid or anything that is not as important?
- What do you think about including information to help people to stop smoking?
    - How would you react to stop-smoking information?
    - Can you think of ways we might be able to include stop-smoking information without putting smokers off?
    - What do you think about telling people where they can get help to stop smoking if they want to, instead of giving people information about stop smoking?

Accessing intervention participants

- Where could we approach people who smoke/used to smoke and have lung symptoms?
  - Probe: through community pharmacies, primary care, community nurses, existing groups for people with lung conditions/ utilising family and social networks / snowballing approaches?
- Are you aware of any community groups for these types of people?
  - How do you think people from these groups would react to being approached for a lung health intervention?

Debrief

'Thank you for taking part in this study. We hope to use the findings from this focus group to develop something to encourage people to go to the doctor with important lung symptoms. Anything you said will be treated as confidential. The voice-recoding will be stored securely. Any quotes used in published research will not have your name or anything that could identify you. Do you have any questions? [*answer any questions*] Here are my contact details if you have any further questions.'

For peer review only
